# Integrative computational epigenomics to build data-driven gene regulation hypotheses

**DOI:** 10.1093/gigascience/giaa064

**Published:** 2020-06-16

**Authors:** Tyrone Chen, Sonika Tyagi

**Affiliations:** 25 Rainforest Walk, School of Biological Sciences, Monash University, Clayton, VIC 3800, Australia

**Keywords:** bioinformatics, computational biology, data integration, deep learning, epigenetics, epigenomics, gene regulation, genomics, high-throughput sequencing, machine learning

## Abstract

**Background:**

Diseases are complex phenotypes often arising as an emergent property of a non-linear network of genetic and epigenetic interactions. To translate this resulting state into a causal relationship with a subset of regulatory features, many experiments deploy an array of laboratory assays from multiple modalities. Often, each of these resulting datasets is large, heterogeneous, and noisy. Thus, it is non-trivial to unify these complex datasets into an interpretable phenotype. Although recent methods address this problem with varying degrees of success, they are constrained by their scopes or limitations. Therefore, an important gap in the field is the lack of a universal data harmonizer with the capability to arbitrarily integrate multi-modal datasets.

**Results:**

In this review, we perform a critical analysis of methods with the explicit aim of harmonizing data, as opposed to case-specific integration. This revealed that matrix factorization, latent variable analysis, and deep learning are potent strategies. Finally, we describe the properties of an ideal universal data harmonization framework.

**Conclusions:**

A sufficiently advanced universal harmonizer has major medical implications, such as (i) identifying dysregulated biological pathways responsible for a disease is a powerful diagnostic tool; (2) investigating these pathways further allows the biological community to better understand a disease’s mechanisms; and (3) precision medicine also benefits from developments in this area, particularly in the context of the growing field of selective epigenome editing, which can suppress or induce a desired phenotype.

## Background

### Importance of data harmonization

**Figure 1:**
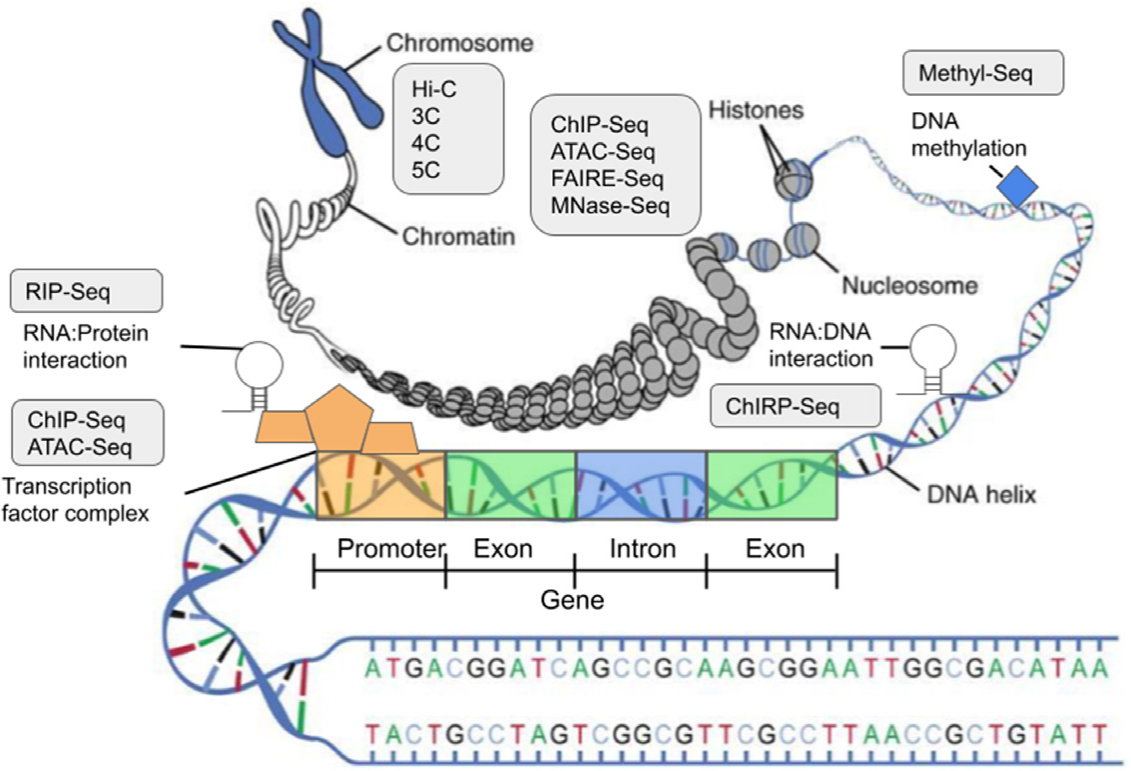
Genomic features affecting gene regulation are shown, along with the corresponding assays used to infer the state of the regulatory feature. We note that this is not an exhaustive list of assays available to profile regulatory features.

**Figure 2:**
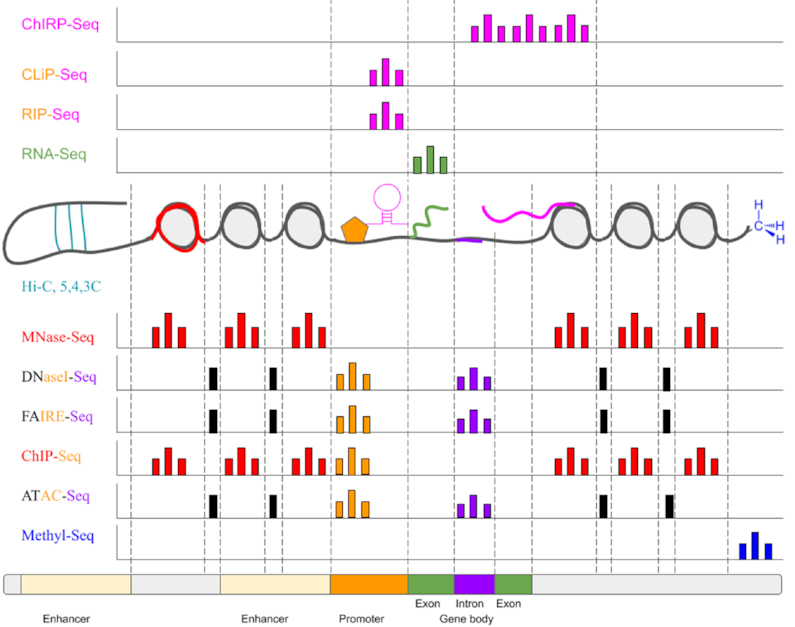
Information returned by types of functional assays targeting RNA-DNA interactions, RNA-protein interactions (including histones), quantifying RNA abundance, DNA-DNA interactions, DNA-protein interactions (including histones), and direct biochemical modifications to DNA. By probing the association of DNA with these regulatory factors, we can observe the activity fingerprint of a genome. Combined with functional outcome information such as gene expression, we can infer the flow of signals that result in a phenotype. (Methyl-Seq normally covers the entire genome, but for simplicity we show coverage at a single methylated site in the hypothetical genome. We note that the performance of some of these assays may vary depending on the experimental design and region targeted [1].)

Answers to gene regulation of disease and normal development lie encrypted in the epigenome. In this context, we define the epigenome as the chromatin state map of the genome and other gene expression–controlling factors (Fig. 1). To capture this state, we require genome-wide measurement of combinations of epigenetic marks occurring in different cell types under various conditions (Fig. 2). Epigenetic systems contributing to this resulting epigenomic state are highly complex and are often the result of multi-layered and combinatorial interactions between different regulatory components of an epigenome [2]. In addition, these interactions are highly dynamic and can vary under different conditions. Therefore, any individual omics assay or data modality results in an incomplete view of a biological system. Recently, the community has been moving towards adopting data-driven approaches to determine gene-specific regulatory pathways of complex phenotypes in cases such as disease progression. This is due to the increasing availability of large-scale high-throughput epigenomic datasets. Therefore, coherently integrating and identifying gene regulatory information across multiple datasets, especially across different types of omics experiments as well as data modalities (such as assay for transposase-accessible chromatin sequencing [ATAC-Seq] [3], chromatin immunoprecipitation sequencing [ChIP-Seq] [4], high-throughput chromosome conformation capture [Hi-C] [5], methylation sequencing [Methyl-Seq] [6–8], and RNA immunoprecipitation sequencing [RIP-Seq] [9] as bulk or single-cell sequencing data), is now an essential and challenging task (Fig. 1). Current attempts are usually limited to a restricted set of modalities, and there are many methods that seem to be integrative at first glance but upon closer investigation have different scopes [10–14]. Unfortunately, no method currently meets this need of capturing a complete cell or tissue state in an accurate and comprehensive way.

Later in this review, we discuss in detail opportunities involved in harmonizing different types of omics data within and across experiments to unlock deeper layers of information present within a biological process involved in disease or complex traits. This holistic genomics approach has been recently gaining momentum [15–19]. Simultaneously harmonizing data, e.g., gene expression with chromatin accessibility, provides an extra layer of validation for the obtained results and reduces the false discovery rate while increasing reproducibility. The user will have a higher degree of confidence in the results owing to their concordance on separate data categories. Together, these complementary methods provide a higher-resolution view into the data, improving our understanding of epigenetic mechanisms of complex diseases as well as traits and subsequently enabling new treatment opportunities.

#### Challenges in data harmonization

Although multi-modal data integration seems attractive, it comes with exponential technical, statistical, and computational challenges. For example, (i) biological data are generated from a wide range of dimensions and from an equally large variety of sources. Dataset heterogeneity results in significant computational issues during analysis, as technical artefacts, dataset complexity, and small sample sizes all contribute to noise in the data. (ii) Furthermore, domain-specific knowledge is required to interpret the results of computational tools, and this requirement is particularly important when considering the specific assumptions that these tools usually make. (3) Analysing data from even a single modality, whether from a single experiment or multiple experiments, is cumbersome because thousands of genes can now be assayed in parallel, generating an equal number of hypotheses. In the case of analysing multimodal data, this challenge is amplified by the non-linear relationship between different omics datasets.

#### Strategies to harmonize data

Integrating information from different resources in a single, unified view can be performed in a variety of ways. While many existing workflows do not explicitly model data modality harmonization, they nevertheless use information from different layers of omics data. Integrating these data can happen at different stages of data processing: primary, intermediate states, or fully processed data. Commonly, integration happens at the final step by repeatedly summarizing primary data from each modality and collapsing them into gene lists, removing a significant amount of quantitative and other forms of valuable information.

**Figure 3:**
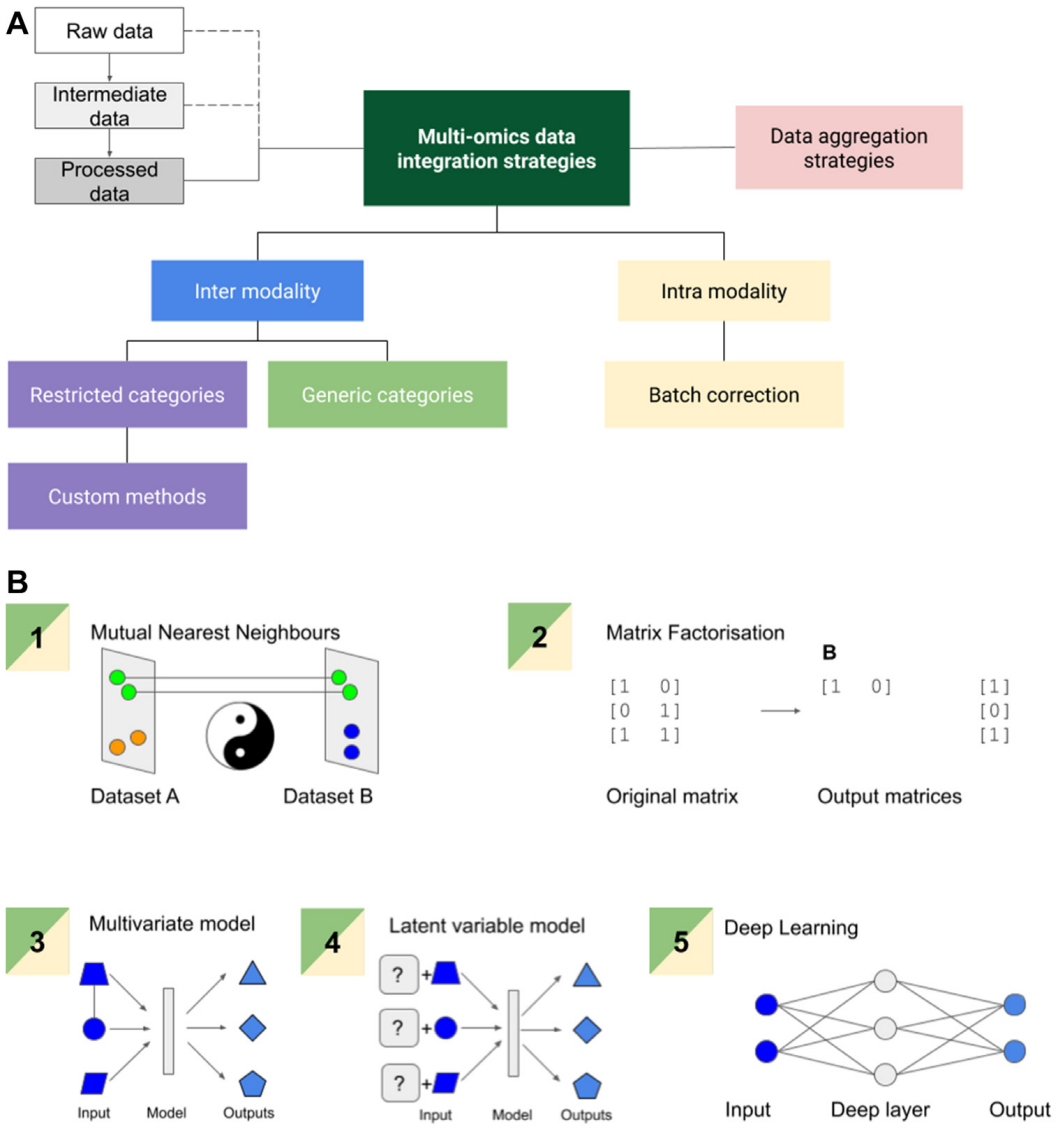
Our proposed functional taxonomy of data integration methods. (A) Inter-modality and intra-modality harmonization methods exist. Data aggregation tools are separate from these methods, which are not data integrators in the context of our review. With inter-modality restricted methods, custom strategies are common. (B) For inter-modality generic methods, 5 approaches are common. Mutual nearest neighbours exploits common points between single-cell datasets as references, matrix factorization operates on abundance measures to categorize data and is agnostic to data type, multivariate models attempt to account for dependent and independent variable contribution to the output, latent variable models attempt to model an unobserved factor’s contribution to the output, and deep learning optimizes a series of regressions to yield a categorical variable or generate an output. Intra-modality harmonization methods share these strategies but apply them specifically to reduce unwanted technical variation. We note that current methods use data in a processed state, and for a better harmonization raw or intermediate data (as shown by the dotted lines in panel A) can be used.

This need for a greater level of biological understanding has given rise to methods that attempt to take a more holistic, inter-omics approach, in contrast to the reductionist approach where hypotheses are modular. These fall into 2 broad categories, (i) targeted data integration (inter-modality restricted) and (ii) general data harmonization (inter-modality free) (Fig. 3). Targeted data integration focuses on integrating 2 or 3 specific data modalities with clear correlations, e.g., the relationship between chromatin occupancy and transcription. Recently, more agnostic data harmonization methods aiming to unify information from an arbitrary number of categories have been emerging [20,21]. In the latter case, there are few such methods available owing to the previously discussed challenges of unifying different data modalities and the relative novelty of this class of approaches. In the rest of the article we examine different types of epigenomic regulatory features, epigenomic data available for regulatory feature detection, and computational methods for building correlation-to-causal gene regulation hypotheses. A critical comprehensive review of these methods has not previously been attempted.

#### Epigenetic regulatory features driving gene activity

Before reviewing these methods, it is first necessary to consider the breadth and mechanisms of epigenetic regulatory features present in the genome. We show that 2 common themes exist among these regulatory features. First, they induce a transcriptionally permissive or repressive environment by altering steric hindrance in DNA towards other regulatory elements. Second, regulatory features rarely act alone, and a signal transduction cascade mediated by multiple regulatory elements is necessary to shape a cell state [22] (see Supplementary Figure S1). Each feature is highly nuanced, and they are not always directly correlated, making direct comparisons difficult.

Gene expression is a tightly modulated process, and perturbations at any step can have negative consequences. Many human diseases are associated with dysregulation of gene expression, including many cancers [23]. Understanding this epigenomic regulatory machinery is therefore crucial to both understanding the biology of any system and applying this information to treat human diseases. Known epigenetic regulatory features are diverse but can be classified into several general categories. At a high level, 3D chromosome structure can be measured with Hi-C [5]. ATAC-Seq captures nucleosome occupancy to reveal the accessibility state of chromatin (open or closed) [3] whereas ChIP-Seq [4] and RIP-Seq [24] capture DNA-protein and RNA-protein binding sites, respectively. Both can probe the occupancy of nucleic acid by regulatory proteins.

It is also possible to capture interactions between as well as within nucleic acids and proteins by means of other immunoprecipitation techniques such as RIP-Seq [9], along with direct biochemical modifications such as methylation to any of these [6–8]. A detailed list of epigenomic features and laboratory assays to study them are provided in Supplementary Table S1.

Many of these features alter the level of steric hindrance in DNA towards other regulatory features. Disruptive DNA loops, chromatin formation, and DNA methylation prevent gene transcription by physically excluding facilitative protein complexes from binding. Conversely, permissive DNA loops, chromatin-free regions, and transcription factors boost the probability of regulatory element binding events by removing or circumventing this barrier.

Although some epigenomic regulatory features have a direct effect on gene expression, gene regulation is often achieved through a web of cause and effect, of which there are abundant examples. Protein biochemical modifications such as histone methylation stabilize associated chromatin to strengthen transcriptional silencing while histone deacetylation has the opposite effect [25–27]. *Cis*-acting long non-coding RNA (lncRNA) can act as a targeted scaffold to bind DNA and proteins to modulate transcriptional permissiveness and are often associated with enhancers [28,29]. (These are distinct from enhancer RNAs, which are shorter and unstable [29]). In some cases, lncRNAs can even encode functional proteins [30]. Other forms of RNA regulation are co-transcribed circular RNA and microRNA (miRNA), which control messenger RNA levels [31–34]. lncRNA can act as an miRNA sponge, thus working in a feedback loop to release or sequester RNA or even protein [35]. This effect extends to transcription factors in the case of lncRNAs GAS5 [36] and RMST [37]. Meanwhile, transcription factors can work in combination with each other, as well as with activator and repressor proteins, to further tune gene expression levels [38]. Metabolite abundance levels in or around the cell may trigger signal cascades through the activation or deactivation of receptor proteins, which result in an upregulation or downregulation of transcription.

After transcription, it is still possible for a cell to selectively calibrate transcript quantity. *Trans*-acting lncRNA stabilizer proteins and functionally equivalent RNA may bind to transcripts to increase their half-life in the cell, or tag them for degradation by enzymes [29]. Extending this ability, *trans*-acting circular RNA can sequester or release transcripts, acting as an RNA battery within the cell [31,39,40].

Genome sequence is by definition not epigenomic, but it is relevant to note that changes in DNA sequence that alter steric hindrance or 3D chromosome structure can directly affect the epigenome. An initial dysregulated binding event may trigger a chain reaction with significant positive or negative effects. At the same time, this highlights the limitations of a reference genome [41]. Despite being a powerful resource, it remains incomplete and is affected by both biological [42] and technical [43] variation just as with any other epigenetic data modality. Thus, informative variation within organisms even of the same species can be masked, decreasing inference accuracy.

### Outline

In this article, we critically review methods that aim to resolve the issue of multi-modal data integration, and classify them into categories on the basis of their scope. In each category, as well as across the field as a whole, we identify common features shared by these strategies and highlight those that show the most potential. At the same time, we suggest a list of epigenomic databases containing the previously discussed gene regulatory features for use by researchers interested in developing or refining such methods, and note the properties of these databases that may positively or negatively affect this process. From this analysis of strategies and databases, we then envision the attributes of an ideal universal data harmonization framework and demonstrate some powerful applications.

## Existing data integration approaches

Many attempts have been made to address the challenges of epigenomic data harmonization both across and within modalities (Tables 1,2,3). These methods are diverse, and we developed a functional taxonomy for the methods surveyed. We filtered out data aggregation methods that group data for viewing, e.g., as Venn diagrams of gene lists, and do not directly integrate data. We then classified integrative methods into 2 categories: intra-modality and inter-modality approaches. Intra-modality approaches focus on removing technical variation caused by batch effects, while inter-modality approaches aim to combine different data modalities thatt may not be directly comparable. Inter-modality data harmonization can be further subclassified into 2 categories, modality restricted and modality free (Fig.   3). The former has a highly restricted scope and is configured to work with a specific experimental design, while the latter is theoretically generalizable to any experimental design.

### Inter-modality restricted

Modality-restricted approaches target a specific set of modalities, often applying modality-specific constraints or assumptions to 2 or 3 data modalities only (Tables 1 and 4). Often, they exploit the strong correlation present between certain data modalities, e.g., in the case of relating chromatin occupancy to transcript expression in EPIP [44] and TEPIC [45]. Furthermore, many methods including LemonTree [46], ELMER [47], and PARADIGM [48] are configured specifically for or tested only on a target category of biological systems, in particular human cancers, sometimes to the extent of being less generalizable to other systems.

**Table 1:** Inter-modality data harmonization approaches with a restricted modality scope

Method name	Strategy	Main advantages	Main limitations	Citation
MDI	Bayesian Consensus Clustering	Identifies gene clusters across datasets with specific shared characteristics. Can model time-series data	Limited to querying a small subset of genes. Trained only on array data	[49]
RIMBANET	Bayesian MCMC	Integrates many data types simultaneously	Requires large quantities of multimodal data. Method was specifically designed for experiment	[50]
EPIP	Ensemble boosting	Effective in unbalanced datasets	Limitations of training data reduce model effectiveness in small datasets	[44]
EAGLE	Ensemble boosting	Uses higher-level features to buffer against overfitting	Custom genome-specific features need to be calculated for classification	[51]
PreSTIGE	Information theory	Outputs different specificity thresholds	Biased to cell type	[52]
TEPIC	Machine learning	Feature space improves result interpretability	Limited performance in gene-dense regions or with small sample sizes	[45]
iOmicsPASS	Network analysis	Produces a sparse set of easily interpretable biological interactions. Effective in heterogeneous datasets	Important markers that are poorly represented in biological networks can be lost in the analysis	[53]
LemonTree	Network analysis; Gibbs sampler; decision tree	Modular model parts for different cases	Trained on cancer data	[46]
PANDA	Network analysis; message passing	Accounts for lack of direct regulatory element interaction	Choice of convergence parameter affects results. Results may be difficult to interpret	[54]
PARADIGM	Network analysis; Probabilistic Graph Model	Robust to false-positive results	Training was performed on microarray data. Effectiveness in sequencing data unknown. Trained on cancer data	[48]
IM-PET	Random forest classifier	Expected to generalize to other species	Requires assembly of 4 manually derived scores	[55]
JEME	Random forest classifier; regression	Easily retrainable on different systems if sufficient data are available	At least 4 input data types are required	[56]
RIPPLE	Random forest classifier; regression	Generalizable to other biological conditions and cell types	Assumes balanced data categories	[57]
SVM-MAP	Support Vector Machine	Expected to generalize to multiple cancer types	Limited enhancer coverage in training data	[58]
ELMER	Wilcoxon rank-sum test	Identifies upstream master regulators	Restricted to methylation arrays in cancer	[47]
TENET	Wilcoxon rank-sum test	Expected to generalize to other biological systems	Targets group expression differences only	[59]
RegNetDriver	Wilcoxon rank-sum test	Provides a framework to construct tissue-specific regulatory networks	Requires assembly of multiple manually derived scores from system-specific steps	[60]

Names, strategies, advantages, and limitations of each method is provided. Regarding advantages and limitations, a few major points were highlighted, and it is important to note that many of these methods are highly nuanced. A citation for reference to the original manuscript of each method is provided where full details can be obtained.

Among these modality-restricted methods, a common theme is the use of ensemble learning, such as random forests [61], on quantitative omics data. Examples of such methods are EPIP [44], EAGLE [51], IM-PET [55], JEME [56], and RIPPLE [57]. Their popularity and success may be attributable to the nature of the algorithm, where an ensemble of clusters representing independent biological signals results in convergence even in heterogeneous data. Notably, these methods appear to be more generalizable than the other methods reviewed (Table 1). Another group of methods approach the problem from a different perspective by applying network analysis to leverage biomolecular interaction information instead of molecule abundance. These methods include LemonTree [46], PANDA [54], and PARADIGM [48]. Owing to the unique angles of each method, formulations of the problem, and applications of the strategy, the advantages and disadvantages of each method vary significantly. These are evaluated in detail in Table 1. One method worth highlighting is RIMBANET [50], which is interesting owing to its ability to integrate data from 6 different modalities including proteomics and metabolomics data, but it is important to note that it was tailored to a specific experiment.

### Inter-modality free

In contrast to modality-restricted approaches, modality-free methods (Tables 2 and 5) are omics-agnostic, to the point of accepting medical imaging data in a few cases. Many of these methods, such as DIABLO [20], iCluster [62], GFA [63], and MOFA [64], use latent variable analysis, and others like NMF [65], iNMF [66], and LIGER [67] use non-negative matrix factorization to harmonize multi-omics data (Fig. 3, Table 2). These categories of methods are particularly viable and flexible because any data can be ingested as long as they can be represented as a generic matrix of values (Fig. 3). DIABLO [20] in particular stands out as a method that was successfully applied to 4 categories of multimodal data, including proteomics and metabolomics. We compare and contrast these methods in more detail in Table 2. At least 1 recent method has successfully combined non-negative matrix factorization with deep learning [21], and this trend of coupling of deep learning to conventional integration strategies is expected to continue given deep learning’s applicability in deconvoluting non-linear relationships in large datasets [68]. We also note that 2 single-cell methods, LIGER [67] as well as seurat [69], are present and observe that bulk RNA-Seq methods and single-cell methods are mutually exclusive.

**Table 2:** Inter-modality data harmonization approaches with a free modality scope

Method name	Strategy	Main advantages	Main limitations	Citation
DeepMF	Deep learning and non-negative matrix factorization	Robust to noise and missing data	Manual parameter tuning and prior information may be required	[70]
JIVE	Dimensionality reduction	Identifies the global modes of variation that drive associations across and within data types	Not robust to outliers, missing values, or class imbalance	[71]
GCCA	Generalized canonical correlation analysis	Identifies blocks of variables within datasets for correlation across datasets	Less effective if the number of observations is smaller than the number of variables or if multiple linear correlations are present between datasets. Biases towards strong variation in the data	[72]
NetICS	Graph diffusion	Robust to frequency of aberrant genes in sample	Can only examine effects of known genes present in a defined interaction network	[73]
DIABLO	Multivariate model and latent variable model	Captures quantitative information. Visual outputs aid interpretation	Assumes a linear relationship between the selected omics features. Parameter tuning is required	[20]
iCluster	Latent variable model	Captures both concordant and unique alterations across data types	Sensitive to initial subset selection. Trained only on array data	[62]
GFA	Latent variable model	Accepts data with missing values	Manual parameter tuning. Prior information may be required	[63]
MOFA	Latent variable model and probabilistic Bayesian	Leverages multiomics to impute missing values. Single-cell version available	Assumes a linear relationship between the selected omics features. Manual parameter tuning required	[74]
seurat	Mutual nearest neighbours	Effective in intra-modality as well as inter-modality integration. Robust to parameter changes	Restricted to single cell. Requires robust reference data	[69]
SNF	Network analysis	Effective in small heterogeneous samples. Captures quantitative information	Does not yield quantitative data. Trained only on array data	[75]
NMF	Non-negative matrix factorization	Accounts for complex modular structures in multimodal data	Trained only on array data	[65]
iNMF	Non-negative matrix factorization	Stable even in heterogeneous conditions	Trained only on array data	[66]
LIGER	Non-negative matrix factorization	Effective in intra-modality as well as inter-modality integration; effective in highly divergent datasets	Restricted to single cell	[67]
sMBPLS	Sparse multi-block partial least-squares regression	Derives weights for modalities indicating contributions to expression	Performance is reduced with lower data dimensions	[76]

Note that seurat and LIGER are specific to single-cell data and the others are intended for bulk data. Names, strategies, advantages, and limitations of each method are provided. Regarding advantages and limitations, a few major points are highlighted. A citation for reference to the original publication of each method is provided where full details can be obtained.

### Intra-modality

A special subset of data harmonization approaches is focused on unifying intra-modality data, and more of these details are available in Table 3. This arose as a result of the common problem in biology of handling unwanted technical variation in data, which can easily be caused by performing experiments on different instruments or on different days [77]. While this class of methods is not directly associated with the broader problem of inter-modality data harmonization, mathematical approaches used to address this problem tend to overlap, such as mutual nearest neighbours in mnnCorrect [78] and seurat [69] (Figure S2). It is highly likely that we can exploit properties of relevant strategies and methods to achieve a better model of data harmonization, e.g., by adding mutual nearest neighbours as an intermediate or refining step to the existing combination of latent variable analysis, matrix factorization, and deep learning.

**Table 3:** Intra-modality data harmonization approaches

Method name	Strategy	Main advantages	Main limitations	Citation
ComBat	Bayesian empirical	Removes batch effect in most cases	Removes biological signal in most cases	[4]
RUV	Linear model	Effective with spike-in controls	Individual variants make specific assumptions about the data	[79]
removeBatchEffect	Linear model	Generalizable to most transcriptomic data types	May be less effective in complex experimental designs	[80]
SVN	Linear model	Generalizable to many cases	Assumes that feature similarities between datasets are due to biology	[81]
mnnCorrect	Mutual nearest neighbours	Accounts for heterogeneity within sample groups	Restricted to single-cell data	[78]
MINT	Multivariate model	Robust to overfitting and strong multidimensional technical variation	Minimum sample count requirement	[82]
Scanorama	Mutual nearest neighbours	Scales to very large sample sizes. Robust to overcorrection	Restricted to single-cell data	[83]
MultiCluster	Tensor decomposition	Accounts for multiple batch variables simultaneously	Restricted to 3-way variable comparisons	[84]
zeroSum	Zero sum regression	Generalizable across different technologies and platforms	Weak or non-linear features may be masked by strong features	[85]

Batch is a special case of intra-modality harmonization and is included for completeness because many underlying strategies used are applicable to broader data integration. All methods are restricted to a single data modality of transcriptomics. Names, strategies, advantages, and limitations of each method are provided. Regarding advantages and limitations, a few major points are highlighted. A citation for reference to the original publication of each method is provided where full details can be obtained.

### Common themes across all methods

Five interesting patterns emerge from this aggregated comparison of methods (Tables 4 and 5). First, gene expression data seem to be universal to all approaches and are often assigned a bridging role across the data modalities. This is likely due to their direct correlation to gene activity, as well as their relative interpretability compared to other omics data, being a straightforward readout of gene activity. Second, quantitative omics data such as gene expression and proteomics are often easier to merge because all data involved are representable as matrices of discrete or continuous values, as opposed to qualitative omics data such as chromosome conformation or chromatin accessibility. Thus, methods merging quantitative omics data such as DIABLO [20] are more generalizable than those merging qualitative and quantitative omics data. Third, data harmonization can be performed at any stage but is commonly performed as a final step with fully processed data. Fourth, single-cell multi-omics harmonization approaches are separate from bulk-cell multi-omics harmonization approaches. This is mainly attributable to the distinct statistical properties between single-cell and bulk cell data, which are not straightforward to reconcile. Fifth, it is not uncommon to observe combinations of strategies within a single method, e.g., the coupling of network analysis to decision trees or regression. The main reason for this is that the complementary nature of different methods working in tandem usually results in a higher-resolution view of a dataset.

**Table 4:** Type and number of data modalities supported by each inter-modality data harmonization approach (restricted modality scope).

Method name	No. of modalities compatible	3D chromosome structure	DNA methylation	Epigenetic peak data	DNA-Protein binding	DNA-RNA interactions	RNA-Protein interactions	Protein-Protein interactions	Genomics	Transcriptomics	Citation
MDI	3	X	X	O	X	X	X	O	X	O	[49]
RIMBANET	4	X	X	X	O	X	X	O	O	O	[50]
EPIP	4	O	X	O	O	X	X	X	X	O	[44]
EAGLE	2	X	X	X	O	X	X	X	X	O	[51]
PreSTIGE	2	X	X	O	X	X	X	X	X	O	[52]
TEPIC	3	O	X	O	X	X	X	X	X	O	[45]
iOmicsPASS	2	X	X	X	X	X	X	X	O	O	[53]
LemonTree	2	X	X	X	X	X	X	X	O	O	[46]
PANDA	3	X	X	X	O	X	X	O	X	O	[54]
PARADIGM	2	X	X	X	X	X	X	X	O	O	[48]
IM-PET	2	X	X	O	X	X	X	X	X	O	[55]
JEME	2	X	X	O	X	X	X	X	X	O	[56]
RIPPLE	3	X	X	O	O	X	X	X	X	O	[57]
SVM-MAP	2	X	O	X	X	X	X	X	X	O	[58]
ELMER	2	X	O	X	X	X	X	X	X	O	[47]
TENET	2	X	O	X	X	X	X	X	X	O	[59]
RegNetDriver	5	X	O	O	O	X	X	X	O	O	[60]

"DNA methylation" in this context refers specifically to the ratio of signal between methylated and unmethylated alleles. For simplicity, some modalities have been aggregated, e.g., transcriptomics data include both gene expression and small RNA data. Some methods are capable of handling proteomics, metabolomics, or medical images, but these are excluded because they are not a focus of this review. A link to each method is provided for easy reference.

**Table 5:** Type and number of data modalities tested by each inter-modality data harmonization approach (free modality scope)

Method name	No. of modalities trained	3D chromosome structure	DNA methylation	Epigenetic peak data	DNA-Protein binding	DNA-RNA interactions	RNA-Protein interactions	Protein-Protein interactions	Genomics	Transcriptomics	Citation
DeepMF	1	X	X	X	X	X	X	X	X	O	[70]
JIVE	1	X	X	X	X	X	X	X	X	O	[71]
GCCA	2	X	X	X	X	X	X	X	O	O	[72]
NetICS	3	X	O	X	X	X	X	X	O	O	[73]
DIABLO	2	X	O	X	X	X	X	X	X	O	[20]
iCluster	3	X	O	X	X	X	X	X	O	O	[62]
GFA	2	X	O	X	X	X	X	X	X	O	[63]
MOFA	2	X	O	X	X	X	X	X	X	O	[74]
seurat*	2	X	X	O	X	X	X	X	X	O	[69]
SNF	2	X	O	X	X	X	X	X	X	O	[75]
NMF	2	X	O	X	X	X	X	X	X	O	[65]
iNMF	2	X	O	X	X	X	X	X	X	O	[66]
LIGER*	2	X	O	X	X	X	X	X	X	O	[67]
sMBPLS	3	X	O	X	X	X	X	X	O	O	[76]

Note that GCCA [72], seurat [69], and LIGER [67] are specific to single-cell data and the others are intended for bulk data. “DNA methylation” in this context refers specifically to the ratio of signal between methylated and unmethylated alleles. In contrast to Table 4, the quantity of modalities represents the quantity of modalities on which the algorithm was tested and does not reflect the modalities with which the algorithm is compatible. For simplicity, some modalities have been aggregated, e.g., transcriptomics data include both gene expression and small RNA data, which gives the illusion that DeepMF [21] and JIVE [71] were trained on unimodal data. Some methods are capable of handling proteomics, metabolomics, or medical images, but these are excluded because they are not a focus of this review. A link to each method is provided for easy reference.

We also emphasize the fact that while some methods may seem to be integrative at first glance, this may not necessarily be the case depending on the application and nuances of the method. For example, a method may combine different data modalities during method development but apply it specifically to signal detection in unimodal data. Deep learning–based methods such as Deepbind [10], BP-Net [12], EP-DNN [11], RE-VAE [13], x-CNN [14], and others commonly fall into this category because models are often trained on multi-modal data but with a restricted goal of identifying motifs in nucleic acids.

### Epigenomic data resources

Therefore, we demonstrate the requirement for a universal data harmonizer. However, before proposing and describing a suitable framework, we first need to discuss suitable training and validation data because this is the most important component in any biological framework and will be the main factor in the resulting viability of a method.

### Data standardization

To develop appropriate models or methods, it is necessary to have a well-curated set of high-quality epigenomic data (see Supplementary Fig. S4B). Currently there is a wealth of biological data available, but not all publicly accessible biological data are standardized or curated to the extent needed by some types of experiments. Standardized laboratory protocols and standardized software pipelines are necessary to limit the effect of unwanted technical variation in the data, which can contribute to significant noise in the data or lead to unintentionally flawed conclusions [77].

### Data accessibility

Furthermore, databases can have specific scopes or restrict access to data, especially pertaining to sensitive patient information (see Supplementary Table S4B). IHEC (International Human Epigenome Consortium) [86] and TCGA (The Cancer Genome Atlas) [87] are the primary examples of this (see Supplementary Table S2). In such cases, users may be limited to non-primary data sources or a restricted subset of samples, which may yield sufficient information depending on the purposes and design of the integrative experiment.

### Data modality

There are a wide variety of epigenomics data modalities present in each database (see Supplementary Table S4A). The choice of database from which to obtain training data for a method should be made on the basis of their individual scopes while taking data standardization and accessibility into account. For example, users interested in human development or disease can select the ENCODE (Encyclopedia of DNA Elements) [88] or Roadmap [89] databases because they contain relevant, standardized, and publicly accessible curated datasets (see Supplementary Table S2). In contrast, users seeking less common datasets associated with rare diseases may not necessarily find the information in a standardized or accessible database and can broaden their search to include ArrayExpress [90], GEO (Gene Expression Omnibus) [91] or INSDC (International Nucleotide Sequence Database Collaboration) [92–94], which stores the data of independent experiments. Quality of data in such cases is not guaranteed, and this is best illustrated with a recent example showing that among several hundred stem cell datasets from these databases, one-third were irreproducible owing to inappropriate experimental design or sample mislabellings [95]. Care should be taken to detect and account for unwanted technical variation in such cases.

## Towards a Universal Data Harmonizer

With such suitable data, we propose and outline the ideal universal data harmonizer, which would be agnostic to input omics type and scale to an arbitrary cardinality of modalities. In addition, it should be easy to use, yield interpretable results, and be robust to noise (see Supplementary Table S3).

### Functionality

A universal data harmonizer has to resolve the previously discussed challenges of distinct omics types and arbitrary cardinality. We re-emphasize the necessity of accounting for non-linear relationships across multimodal datasets. While many existing methods yield results by exploiting strong correlations between specific omics data, e.g., between gene expression and chromatin accessibility [55–57], this does not hold true in all cases. One scenario where this is particularly visible is the relationship between DNA methylation and chromosome conformation, where simple linear correlation is unlikely to be effective in predicting a state given information about the other. Therefore, a universal data harmonizer will not be able to take advantage of linear correlations in all situations and will have to be designed to be agnostic in this context.

To address the equally challenging problem of cardinality, the universal harmonizer needs to infer properties directly from the data instead of imposing broad conditions or constraints. Intuitively, it may first appear that flooding a method with multiple layers of information may allow easier signal detection. However, increasing the quantity of modalities surveyed may further amplify the non-linear relationship between omics datasets, adding noise to the data. Furthermore, different features may be either sparse or enriched in different omics datasets and combinations of datasets.

### Usability

In the context of usability, data should have to undergo minimal preprocessing. This is advantageous for 2 reasons; preserving method input data in a state as close to primary data as possible, e.g., in the form of raw sequence data, allows the user to promptly and easily analyse their data. More importantly, assumptions associated with preprocessing or intermediate data analyses are avoided. For the same reason, the method should require minimal parameters. This both lessens user confusion while allowing signals to rise organically from the data. Furthermore, the method should be reasonably generalizable or at least sufficiently flexible to account for unconventional cases. Because it is unlikely that any single method is applicable to every possible combination of highly nuanced biological datasets, a method should be reconfigurable depending on a biological domain of interest to account for such cases. For example, deep learning models should be designed to be easily re-trainable on data as long as they are formatted correctly, with possible minor adjustments to model architecture.

To further maximize efficiency, the model should be as computationally efficient as possible. Many workflows can consume significant quantities of data storage, memory, and compute time [96–98], to the extent that handling these issues can require a greater resource investment than the actual experiment [99]. In these scenarios, high-performance computing clusters may be required to implement methods, which may not be easily accessible to all users.

For easy installation and reproducibility in line with FAIR (findable, accessible, interoperable, reusable) data principles [100], the software should contain only required software libraries. A lower degree of portability forces a user to unnecessarily invest resources into managing multiple versions of potentially clashing dependencies and sub-dependencies of software. This is prevalent even among high-quality and widely used programs, e.g., in the R [101] and Bioconductor [102,103] ecosystem of biological software. Although virtual environment management libraries exist to address this problem [104], they may not be readily accessible or known to new users. Including this software in well-maintained biological software channels with mature dependency management systems like conda [105] or providing them as a virtual machine environment such as Docker [106] or singularity [107] removes a large barrier to user adoption of software.

### Interpretability

Results of an ideal universal harmonizer should also be easily interpretable. For instance, it is more intuitive for a biologist to understand a method that highlights a gene pathway of interest, instead of a matrix of values that may require additional processing. Where practical, visualizations should be provided to assist interpretation and supplement other results, and presented in easily accessible and portable formats such as an html report or pdf file [108]. However, objective metrics are equally important to judge the performance of an algorithm on a dataset, such as true- and false-positive rate. This protects the user from jumping to misleading conclusions.

### Robustness

All methods are vulnerable to technical and biological noise, and a universal harmonizer should be robust against these. From a biological perspective, missing values may occur in omics datasets, which results in data that are difficult to compare directly without imputation or other rescue steps.

Further complicating this are technical factors such as class imbalance and sample sizes. An imbalance in sample categories may limit the effectiveness of biological datasets. We take the case of a recent SARS-Cov-2 patient study as an example [109]. In this, 2 imbalanced sample classes were contrasted with a 3-fold difference in sample representation across classes. While this was likely unavoidable owing to the disruptive effects of the ongoing COVID-19 (coronavirus disease 2019) pandemic, this may skew a fragile algorithm towards features in the overrepresented category. This is especially true in cases where 2 conditions may be closely related or may have a similar signal fingerprint to begin with, such as SARS (severe acute respiratory syndrome) and COVID-19 [110,111]. Conclusions drawn from such studies can potentially have global effects on diagnostic tests and health policies, with downstream effects on public health. Meanwhile, an insufficient sample size may reduce an algorithm’s effectiveness or lead to false-positive signals from its limited feature set. Finally, technical variation in the data has the potential to significantly contaminate results with non-biological noise and should be avoided where possible [77]. These 3 technical problems can be avoided with careful experimental design, but in some cases this may not be possible, especially in situations where sample mass is limited or special biological conditions are under study (e.g., rare phenotypes or geographical, social, economical, and political barriers). Intra-modality harmonization methods can buffer some of this irrelevant variation (Table 3) but have their limitations.

### Applications

There is a wide range of potential applications for a sufficiently advanced universal harmonizer across all fields of biology. On a general level, identifying biological pathways contributing to a phenotype allows a user to establish a molecular fingerprint for an organism’s phenotype or cell state. Given one piece of information, the user can then infer the state of the other. In medicine, applying this technique to a patient will improve the speed and accuracy of clinical diagnoses.

With this knowledge, an opportunity to achieve an intended phenotype by targeting the appropriate biomolecular switches exists. Epigenomic editing is still in its infancy but is an active area of research with profound clinical implications across all diseases [112,113]. CRISPR-Cas9 [114] or small RNA-mediated methods have potential in treating complex diseases such as cystic fibrosis by suppressing the mucin production machinery or by inducing the production of functional CFTR variants [115]. A similar approach reversed an intellectual disability phenotype in mice [116]. The field of cancer research and treatment is likely to benefit from this as well because it involves the dysregulation of many pathways and is challenging to treat with conventional therapies [113]. From the opposite perspective, it will be possible to also identify drug adverse effects by examining the biological pathways they will affect. Clinicians can then design mitigating strategies.

Overall, knowing the biological pathways involved in a complex phenotype at the very least highlights them for further investigation. A deeper understanding of biology will result, which feeds back positively into all possible applications.

## Conclusion

To minimize confusion around method scope, we developed a classification system for data integrative strategies. We define ”data harmonization” as the unification of low-level features across different data modalities and distinguish this from the broader, inconsistent usage of "data integration" in the literature.

Although substantial barriers to universal multimodal data harmonization exist, we highlight several points and strategies of interest, which some existing harmonization methods already account for and implement. To resolve the heterogeneity across different epigenomics datasets, transcriptomics data are used in all state-of-the-art methods as a reference point because their properties are relatively well understood and they are commonly used in experiments. A hypothetical universal harmonizer can take advantage of this property by making the reasonable assumption that transcriptomics data will be present and using this to anchor a method. This can further act as a bridge between epigenomic and functional omics data, allowing protein and metabolite information to be included. Combining these epigenomic and functional signatures allows a system-level view to be obtained.

A fundamental problem in data harmonization is to consistently represent heterogeneous epigenomics datasets. A possible solution is reformulating data as generic matrices matched on samples, allowing the use of flexible techniques such as matrix factorization. In addition, the rising field of deep learning is capable of resolving non-linear relationships in large complex datasets and is therefore well suited to this task. Substantial advancements in multimodal data harmonization are expected by applying a combination of these strategies, and applying this to unlock the full power of both existing and future biological datasets will remove a major bottleneck of systems biology, unlocking a new paradigm of medical applications.

## Additional Files

Supplementary Figure S1. Gene expression is the result of a combination of regulatory feature interactions.

Supplementary Figure S2.

Supplementary Figure S3.

Supplementary Figure S4.

Supplementary Table S1. Epigenomic regulatory features and their corresponding assays.

Supplementary Table S2. Epigenomic data resources and their scope.

Supplementary Table S3. Properties of an ideal universal dataset harmoniser.

Supplementary Table S4. Data modalities, accessibility and standardisation present in epigenomic databases.

## Abbreviations

ATAC-Seq: assay for transposase-accessible chromatin; ChIP-Seq: chromatin immunoprecipitation sequencing; ChIRP-Seq: chromatin isolation by RNA purification sequencing; CLiP-Seq: cross-linking immunoprecipitation sequencing; COVID-19: coronavirus disease 2019; CTFR: cystic fibrosis transmembrane conductance regulator; CRISPR-Cas9: Clustered Regularly Interspaced Short Palindromic Repeats and CRISPR protein 9; DDBJ: DNA Databank of Japan; DIABLO: Data Integration Analysis for Biomarker Discovery Using Latent Components; DNase-Seq: DNase I hypersensitive site sequencing; EBI: European Bioinformatics Institute; EPIP: enhancer-promoter interaction prediction; FAIR: findable, accessible, interoperable, reusable; FAIRE-Seq: formaldehyde-assisted isolation of regulatory elements; GCCA: generalized canonical correlation analysis; GFA: group factor analysis; Hi-C: high-throughput chromosome conformation capture; IHEC: International Human Epigenomic Consortium; INSDC: International Nucleotide Sequence Database Collaboration; JIVE: joint and individual variation explained; LIGER: Linked Inference of Genomic Experimental Relationships; lncRNA: long non-coding RNA; MCMC: Markov chain Monte Carlo; Methyl-Seq: methylation sequencing; miRNA: microRNA; MNase-Seq: micrococcal nuclease sequencing; MOFA: Multi-Omics Factor Analysis; NMF: non-negative matrix factorization; RE-VAE: Roadmap-ENCODE Variational Auto-Encoder; RIP-Seq: RNA immunoprecipitation sequencing; RNA-Seq: RNA sequencing; RUV: Remove Unwanted Variation; SARS: severe acute respiratory syndrome; sMBPLS: sparse multi-block partial least-squares regression; SRA: Sequence Read Archive; TAD: topologically associating domain; TCGA: The Cancer Genome Atlas; TF: transcription factor; TFBS: transcription factor binding site.

## Competing Interests

The authors declare that they have no competing interests.

## Funding

S.T. acknowledges funding from the Faculty Initiative Fund and Australian Women Research Success Grant at Monash University. T.C. received funding from the Australian Government Research Training Program Scholarship and Monash Faculty of Science Dean’s Postgraduate Research Scholarship.

## Authors' Contributions

Conceptualization, S.T.; formal analysis, both authors; funding acquisition, S.T.; investigation, both authors; resources, S.T.; supervision, S.T.; validation, both authors; visualization, both authors; writing—original draft, both authors; writing—review and editing, both authors.

## Authors’ Information

S.T. is head of the computational biology research group and a research affiliate with the eResearch Centre at Monash University, Australia, with >15 years of experience in bioinformatics. T.C. is a Ph.D. candidate in computational biology with the computational biology research group at Monash University, Australia, with over >5 years of experience in computational biology. Both authors have an interest in developing tools to uncover knowledge from biological data.

## Acknowledgements

We thank Dianne Cook and Elizabeth Mason for helpful feedback. We acknowledge and pay respects to the Elders and Traditional Owners of the land on which our 4 Australian campuses stand.

## Supplementary Material

giaa064_GIGA-D-20-00089_Original_Submission

giaa064_GIGA-D-20-00089_Revision_1

giaa064_Response_to_Reviewer_Comments_Original_Submission

giaa064_Reviewer_1_Report_Original_Submission Marcel Holger Schulz -- 5/17/2020 Reviewed

giaa064_Supplemental_File

## References

[1] Nordström KJV, Schmidt F, Gasparoni N, et al. Unique and assay specific features of NOMe-, ATAC- and DNase I-seq data. Nucleic Acids Res. 2019;47(20):10580–96. 31584093 10.1093/nar/gkz799 PMC6847574

[2] Stricker SH, Köferle A, Beck S From profiles to function in epigenomics. Nat Rev Genet. 2016;18(1):51–66. 27867193 10.1038/nrg.2016.138

[3] Buenrostro JD, Giresi PG, Zaba LC, et al. Transposition of native chromatin for fast and sensitive epigenomic profiling of open chromatin, DNA-binding proteins and nucleosome position. Nat Methods. 2013;10(12):1213–8. 24097267 10.1038/nmeth.2688 PMC3959825

[4] Johnson DS, Mortazavi A, Myers RM, et al. Genome-wide mapping of in vivo protein-DNA interactions. Science. 2007;316(5830):1497–502. 17540862 10.1126/science.1141319

[5] Lieberman-Aiden E, Berkum NLV, Williams L, et al. Comprehensive mapping of long-range interactions reveals folding principles of the human genome. Science. 2009;33292:289–94. 10.1126/science.1181369 PMC2858594 19815776

[6] Frommer M, McDonald LE, Millar DS, et al. A genomic sequencing protocol that yields a positive display of 5-methylcytosine residues in individual DNA strands. Proc Natl Acad Sci U S A. 1992;89(5):1827–31. 1542678 10.1073/pnas.89.5.1827 PMC48546

[7] Meissner A, Gnirke A, Bell GW, et al. Reduced representation bisulfite sequencing for comparative high-resolution DNA methylation analysis. Nucleic Acids Res. 2005;33(18):5868–77. 16224102 10.1093/nar/gki901 PMC1258174

[8] Meissner A, Mikkelsen TS, Gu H, et al. Genome-scale DNA methylation maps of pluripotent and differentiated cells. Nature. 2008;454(7205):766–70. 18600261 10.1038/nature07107 PMC2896277

[9] Chu C, Qu K, Zhong F, et al. Genomic maps of long noncoding RNA occupancy reveal principles of RNA-chromatin interactions. Mol Cell. 2011;44(4):667–78. 21963238 10.1016/j.molcel.2011.08.027 PMC3249421

[10] Alipanahi B, Delong A, Weirauch MT, et al. Predicting the sequence specificities of DNA- and RNA-binding proteins by deep learning. Nat Biotechnol. 2015;33(8):831–8. 26213851 10.1038/nbt.3300

[11] Kim SG, Harwani M, Grama A, et al. EP-DNN: A deep neural network-based global enhancer prediction algorithm. Sci Rep. 2016;6, doi:10.1038/srep38433. PMC5144062 27929098

[12] Avsec Ž, Weilert M, Shrikumar A, et al. Deep learning at base-resolution reveals motif syntax of the cis-regulatory code. bioRxiv. 2019, doi:10.1101/737981.

[13] Hu R, Pei G, Jia P, et al. Decoding regulatory structures and features from epigenomics profiles: A Roadmap-ENCODE Variational Auto-Encoder (RE-VAE) model. Methods. 2019, doi:10.1016/j.ymeth.2019.10.012. PMC7431277 31672653

[14] Jaroszewicz A, Ernst J An integrative approach for fine-mapping chromatin interactions. Bioinformatics. 2020;36(6):1704–11. 31742318 10.1093/bioinformatics/btz843 PMC7425030

[15] Hussein SMI, Puri MC, Tonge PD, et al. Genome-wide characterization of the routes to pluripotency. Nature. 2014;516(7530):198–206. 25503233 10.1038/nature14046

[16] Moor AE, Golan M, Massasa EE, et al. Global mRNA polarization regulates translation efficiency in the intestinal epithelium. Science. 2017;357(6357):1299–303. 28798045 10.1126/science.aan2399 PMC5955215

[17] Shah S, Takei Y, Zhou W, et al. Dynamics and spatial genomics of the nascent transcriptome by intron seqFISH. Cell. 2018;174(2):363–76. 29887381 10.1016/j.cell.2018.05.035 PMC6046268

[18] Wan Y, Wei Z, Looger LL, et al. Single-cell reconstruction of emerging population activity in an entire developing circuit. Cell. 2019;179(2):355–72. 31564455 10.1016/j.cell.2019.08.039 PMC7055533

[19] Schier AF Single-cell biology: Beyond the sum of its parts. Nat Methods. 2020;17:17–20. 31907464 10.1038/s41592-019-0693-3

[20] Singh A, Shannon CP, Gautier B, et al. DIABLO: An integrative approach for identifying key molecular drivers from multi-omics assays. Bioinformatics. 2019;35(17):3055–62. 30657866 10.1093/bioinformatics/bty1054 PMC6735831

[21] Chen L, Xu J, Li SC DeepMF: Deciphering the latent patterns in omics profiles with a deep learning method. BMC Bioinformatics. 2019;20(Suppl 23):1–13. 31881818 10.1186/s12859-019-3291-6 PMC6933662

[22] Vogelstein B, Kinzler KW Cancer genes and the pathways they control. Nat Med. 2004;10(8):789–99. 15286780 10.1038/nm1087

[23] Flavahan WA, Drier Y, Johnstone SE, et al. Altered chromosomal topology drives oncogenic programs in SDH-deficient GISTs. Nature. 2019;575(7781):229–33. 31666694 10.1038/s41586-019-1668-3 PMC6913936

[24] Zhao J, Ohsumi TK, Kung JT et al. Genome-wide identification of polycomb-associated RNAs by RIP-seq. Mol Cell. 2010;40(6):939–953. 21172659 10.1016/j.molcel.2010.12.011 PMC3021903

[25] Brower-Toland B, Wacker DA, Fulbright RM, et al. Specific contributions of histone tails and their acetylation to the mechanical stability of nucleosomes. J Mol Biol. 2005;346(1):135–46. 15663933 10.1016/j.jmb.2004.11.056

[26] Collings CK, Waddell PJ, Anderson JN Effects of DNA methylation on nucleosome stability. Nucleic Acids Res. 2013;41(5):2918–31. 23355616 10.1093/nar/gks893 PMC3597673

[27] Lorch Y, Maier-Davis B, Kornberg RD Histone acetylation inhibits RSC and stabilizes the +1 nucleosome. Mol Cell. 2018;72(3):594–600. 30401433 10.1016/j.molcel.2018.09.030 PMC6290470

[28] Qian Z, Zhurkin VB, Adhya S DNA–RNA interactions are critical for chromosome condensation in *Escherichia coli*. Proc Natl Acad Sci U S A. 2017;114(46):12225–30. 29087325 10.1073/pnas.1711285114 PMC5699063

[29] Gil N, Ulitsky I Regulation of gene expression by cis-acting long non-coding RNAs. Nat Rev Genet. 2020;21(2):102–17. 31729473 10.1038/s41576-019-0184-5

[30] Stein CS, Jadiya P, Zhang X, et al. Mitoregulin: A lncRNA-encoded microprotein that supports mitochondrial supercomplexes and respiratory efficiency. Cell Rep. 2018;23(13):3710–20. 29949756 10.1016/j.celrep.2018.06.002 PMC6091870

[31] Mongelli A, Martelli F, Farsetti A, et al. The dark that matters: Long noncoding RNAs as master regulators of cellular metabolism in noncommunicable diseases. Front Physiol. 2019;10:369. 31191327 10.3389/fphys.2019.00369 PMC6539782

[32] Ashwal-Fluss R, Meyer M, Pamudurti NR, et al. CircRNA Biogenesis competes with pre-mRNA splicing. Mol Cell. 2014;56(1):55–66. 25242144 10.1016/j.molcel.2014.08.019

[33] Fire A, Xu S, Montgomery MK, et al. Potent and specific genetic interference by double-stranded RNA in *Caenorhabditis elegans*. Nature. 1998;391:806–11. 9486653 10.1038/35888

[34] Waterhouse PM, Graham MW, Wang MB Virus resistance and gene silencing in plants can be induced by simultaneous expression of sense and antisense RNA. Proc Natl Acad Sci U S A. 1998;95(23):13959–64. 9811908 10.1073/pnas.95.23.13959 PMC24986

[35] Zhang X, Zhou Y, Chen S, et al. LncRNA MACC1-AS1 sponges multiple miRNAs and RNA-binding protein PTBP1. Oncogenesis. 2019;8(12), doi:10.1038/s41389-019-0182-7. PMC6904680 31822653

[36] Hou XX, Cheng H Long non-coding RNA RMST silencing protects against middle cerebral artery occlusion (MCAO)-induced ischemic stroke. Biochem Biophys Res Commun. 2018;495(4):2602–8. 29258823 10.1016/j.bbrc.2017.12.087

[37] Schmitt AM, Garcia JT, Hung T, et al. An inducible long noncoding RNA amplifies DNA damage signaling. Nat Genet. 2016;48(11):1370–6. 27668660 10.1038/ng.3673 PMC5083181

[38] Jacob F, Monod J Genetic regulatory mechanisms in the synthesis of proteins. J Mol Biol. 1961;3(3):318–56. 13718526 10.1016/s0022-2836(61)80072-7

[39] Hansen TB, Jensen TI, Clausen BH, et al. Natural RNA circles function as efficient microRNA sponges. Nature. 2013;495(7441):384–8. 23446346 10.1038/nature11993

[40] Barrett SP, Salzman J Circular RNAs: Analysis, expression and potential functions. Development. 2016;143(11):1838–47. 27246710 10.1242/dev.128074 PMC4920157

[41] Ballouz S, Dobin A, Gillis JA, Is it time to change the reference genome?. Genome Biol. 2019;20(1):159. 31399121 10.1186/s13059-019-1774-4 PMC6688217

[42] Cho YS, Kim H, Kim HM, et al. An ethnically relevant consensus Korean reference genome is a step towards personal reference genomes. Nat Commun. 2016;7:13637. 27882922 10.1038/ncomms13637 PMC5123046

[43] Alkan C, Sajjadian S, Eichler EE Limitations of next-generation genome sequence assembly. Nat Methods. 2011;8(1):61–5. 21102452 10.1038/nmeth.1527 PMC3115693

[44] Talukder A, Saadat S, Li X, et al. EPIP: A novel approach for condition-specific enhancer-promoter interaction prediction. Bioinformatics. 2019;35(20):3877–83. 31410461 10.1093/bioinformatics/btz641 PMC7963088

[45] Schmidt F, Kern F, Schulz MH, Integrative prediction of gene expression with chromatin accessibility and conformation data. Epigenetics Chromatin. 2020;13:4. 32029002 10.1186/s13072-020-0327-0 PMC7003490

[46] Bonnet E, Calzone L, Michoel T Integrative multi-omics module network inference with Lemon-Tree. PLoS Comput Biol. 2015;11(2), doi:10.1371/journal.pcbi.1003983. PMC4332478 25679508

[47] Silva TC, Coetzee SG, Gull N, et al. ELmer v.2: An r/bioconductor package to reconstruct gene regulatory networks from DNA methylation and transcriptome profiles. Bioinformatics. 2019;35(11):1974–7. 30364927 10.1093/bioinformatics/bty902 PMC6546131

[48] Vaske CJ, Benz SC, Sanborn JZ, et al. Inference of patient-specific pathway activities from multi-dimensional cancer genomics data using PARADIGM. Bioinformatics. 2010;26(12):237–45. 10.1093/bioinformatics/btq182 PMC2881367 20529912

[49] Kirk P, Griffin JE, Savage RS, et al. Bayesian correlated clustering to integrate multiple datasets. Bioinformatics. 2012;28(24):3290–7. 23047558 10.1093/bioinformatics/bts595 PMC3519452

[50] Zhu J, Sova P, Xu Q, et al. Stitching together multiple data dimensions reveals interacting metabolomic and transcriptomic networks that modulate cell regulation. PLoS Biol. 2012;10(4), doi:10.1371/journal.pbio.1001301. PMC3317911 22509135

[51] Gao T, Qian J Eagle: An algorithm that utilizes a small number of genomic features to predict tissue/ cell type-specific enhancer-gene interactions. PLoS Comput Biol. 2019;15(10), doi:10.1371/journal.pcbi.1007436. PMC6821050 31665135

[52] Corradin O, Saiakhova A, Akhtar-Zaidi B, et al. Combinatorial effects of multiple enhancer variants in linkage disequilibrium dictate levels of gene expression to confer susceptibility to common traits. Genome Res. 2014;24(1), doi:10.1101/gr.164079.113. PMC3875850 24196873

[53] Koh HWL, Fermin D, Vogel C, et al. iOmicsPASS: Network-based integration of multiomics data for predictive subnetwork discovery. NPJ Syst Biol Appl. 2019;5(1), doi:10.1038/s41540-019-0099-y. PMC6616462 31312515

[54] Glass K, Huttenhower C, Quackenbush J, et al. Passing messages between biological networks to refine predicted interactions. PLoS One. 2013;8(5), doi:10.1371/journal.pone.0064832. PMC3669401 23741402

[55] He B, Chen C, Teng L, et al. Global view of enhancer-promoter interactome in human cells. Proc Natl Acad Sci U S A. 2014;111(21):E2191–9. 24821768 10.1073/pnas.1320308111 PMC4040567

[56] Cao Q, Anyansi C, Hu X, et al. Reconstruction of enhancer-target networks in 935 samples of human primary cells, tissues and cell lines. Nat Genet. 2017;49(10):1428–36. 28869592 10.1038/ng.3950

[57] Roy S, Siahpirani AF, Chasman D, et al. A predictive modeling approach for cell line-specific long-range regulatory interactions. Nucleic Acids Res. 2015;43(18):8694–712. 26338778 10.1093/nar/gkv865 PMC4605315

[58] Aran D, Sabato S, Hellman A, DNA methylation of distal regulatory sites characterizes dysregulation of cancer genes. Genome Biol. 2013;14(3), doi:10.1186/gb-2013-14-3-r21. PMC4053839 23497655

[59] Rhie SK, Guo Y, Tak YG, et al. Identification of activated enhancers and linked transcription factors in breast, prostate, and kidney tumors by tracing enhancer networks using epigenetic traits. Epigenetics Chromatin. 2016;9(1), doi:10.1186/s13072-016-0102-4. PMC5103450 27833659

[60] Dhingra P, Martinez-Fundichely A, Berger A, et al. Identification of novel prostate cancer drivers using RegNetDriver: A framework for integration of genetic and epigenetic alterations with tissue-specific regulatory network. Genome Biol. 2017;18(1), doi:10.1186/s13059-017-1266-3. PMC5530464 28750683

[61] Ho TK Random decision forests. In: ICDAR '95: Proceedings of the Third International Conference on Document Analysis and Recognition. Washington, DC: IEEE; 1995:278–82.

[62] Shen R, Olshen AB, Ladanyi M Integrative clustering of multiple genomic data types using a joint latent variable model with application to breast and lung cancer subtype analysis. Bioinformatics. 2009;25(22):2906–12. 19759197 10.1093/bioinformatics/btp543 PMC2800366

[63] Leppäaho E, Ammad-Ud-Din M, Kaski S GFA: Exploratory analysis of multiple data sources with group factor analysis. J Mach Learn Res. 2017;18:1–5.

[64] Argelaguet R, Arnol D, Bredikhin D, et al. MOFA+: A probabilistic framework for comprehensive integration of structured single-cell data. bioRxiv. 2019, doi:10.1101/837104. PMC7212577 32393329

[65] Zhang S, Liu CC, Li W, et al. Discovery of multi-dimensional modules by integrative analysis of cancer genomic data. Nucleic Acids Res. 2012;40(19):9379–91. 22879375 10.1093/nar/gks725 PMC3479191

[66] Yang Z, Michailidis G A non-negative matrix factorization method for detecting modules in heterogeneous omics multi-modal data. Bioinformatics. 2016;32(1):1–8. 26377073 10.1093/bioinformatics/btv544 PMC5006236

[67] Welch JD, Kozareva V, Ferreira A, et al. Single-cell multi-omic integration compares and contrasts features of brain cell identity. Cell. 2019;177(7):1873–87. 31178122 10.1016/j.cell.2019.05.006 PMC6716797

[68] Ching T, Himmelstein DS, Beaulieu-Jones BK, et al. Opportunities and obstacles for deep learning in biology and medicine. J R Soc Interface. 2018;15(141), doi:10.1098/rsif.2017.0387. PMC5938574 29618526

[69] Stuart T, Butler A, Hoffman P, et al. Comprehensive integration of single-cell data. Cell. 2019;177(7):1888–902. 31178118 10.1016/j.cell.2019.05.031 PMC6687398

[70] Chen L, Xu J, Li SC DeepMF: Deciphering the latent patterns in omics profiles with a deep learning method. BMC Bioinformatics. 2019;20(23):648. 31881818 10.1186/s12859-019-3291-6 PMC6933662

[71] Lock EF, Hoadley KA, Marron JS, et al. Joint and individual variation explained (JIVE) for integrated analysis of multiple data types. Ann Appl Stat. 2013;7(1):523–42. 23745156 10.1214/12-AOAS597 PMC3671601

[72] Tenenhaus A, Philippe C, Guillemot V, et al. Variable selection for generalized canonical correlation analysis. Biostatistics. 2014;15(3):569–83. 24550197 10.1093/biostatistics/kxu001

[73] Dimitrakopoulos C, Hindupur SK, Hafliger L, et al. Network-based integration of multi-omics data for prioritizing cancer genes. Bioinformatics. 2018;34(14):2441–8. 29547932 10.1093/bioinformatics/bty148 PMC6041755

[74] Argelaguet R, Velten B, Arnol D, et al. Multi–Omics Factor Analysis–a framework for unsupervised integration of multi–omics data sets. Mol Syst Biol. 2018;14(6):e8124. 29925568 10.15252/msb.20178124 PMC6010767

[75] Wang B, Mezlini AM, Demir F, et al. Similarity network fusion for aggregating data types on a genomic scale. Nat Methods. 2014;11(3):333–7. 24464287 10.1038/nmeth.2810

[76] Li W, Zhang S, Liu CC, et al. Identifying multi-layer gene regulatory modules from multi-dimensional genomic data. Bioinformatics. 2012;28(19):2458–66. 22863767 10.1093/bioinformatics/bts476 PMC3463121

[77] Leek JT, Scharpf RB, Bravo HC, et al. Tackling the widespread and critical impact of batch effects in high-throughput data. Nat Rev Genet. 2010;11(10):733–39. 20838408 10.1038/nrg2825 PMC3880143

[78] Haghverdi L, Lun ATL, Morgan MD, et al. Batch effects in single-cell RNA-sequencing data are corrected by matching mutual nearest neighbors. Nat Biotechnol. 2018;36(5):421–7. 29608177 10.1038/nbt.4091 PMC6152897

[79] Risso D, Ngai J, Speed TP, et al. Normalization of RNA-seq data using factor analysis of control genes or samples. Nat Biotechnol. 2014;32(9):896–902. 25150836 10.1038/nbt.2931 PMC4404308

[80] Ritchie ME, Phipson B, Wu D, et al. Limma powers differential expression analyses for RNA-sequencing and microarray studies. Nucleic Acids Res. 2015;43(7):e47. 25605792 10.1093/nar/gkv007 PMC4402510

[81] Mecham BH, Nelson PS, Storey JD Supervised normalization of microarrays. Bioinformatics. 2010;26(10):1308–15. 20363728 10.1093/bioinformatics/btq118 PMC2865860

[82] Rohart F, Eslami A, Matigian N, et al. MINT: A multivariate integrative method to identify reproducible molecular signatures across independent experiments and platforms. BMC Bioinformatics. 2017;18(1):128. 28241739 10.1186/s12859-017-1553-8 PMC5327533

[83] Hie B, Bryson B, Berger B Efficient integration of heterogeneous single-cell transcriptomes using Scanorama. Nat Biotechnol. 2019;37(6):685–91. 31061482 10.1038/s41587-019-0113-3 PMC6551256

[84] Wang M, Fischer J, Song YS Three-way clustering of multi-tissue multi-individual gene expression data using semi-nonnegative tensor decomposition. bioRxiv. 2017, doi:10.1101/229245. PMC7771883 33381253

[85] Altenbuchinger M, Schwarzfischer P, Rehberg T, et al. Molecular signatures that can be transferred across different omics platforms. Bioinformatics. 2017;33(14):i333–40. 28881975 10.1093/bioinformatics/btx241 PMC5870545

[86] Stunnenberg HG, Abrignani S, Adams D, et al. The International Human Epigenome Consortium: A blueprint for scientific collaboration and discovery. Cell. 2016;167(5):1145–9. 27863232 10.1016/j.cell.2016.11.007

[87] Tomczak K, Czerwińska P, Wiznerowicz M The Cancer Genome Atlas (TCGA): An immeasurable source of knowledge. Contemp Oncol (Pozn). 2015;1A:A68–A77. 10.5114/wo.2014.47136 PMC4322527 25691825

[88] Davis CA, Hitz BC, Sloan CA, et al. The Encyclopedia of DNA elements (ENCODE): Data portal update. Nucleic Acids Res. 2018;46(D1):D794–D801. 29126249 10.1093/nar/gkx1081 PMC5753278

[89] Bernstein BE, Stamatoyannopoulos JA, Costello JF, et al. The NIH roadmap epigenomics mapping consortium. Nat Biotechnol. 2010;28(10):1045–8. 20944595 10.1038/nbt1010-1045 PMC3607281

[90] Athar A, Füllgrabe A, George N, et al. ArrayExpress update - From bulk to single-cell expression data. Nucleic Acids Res. 2019;47(D1):D711–5. 30357387 10.1093/nar/gky964 PMC6323929

[91] Barrett T, Wilhite SE, Ledoux P, et al. NCBI GEO: Archive for functional genomics data sets - Update. Nucleic Acids Res. 2013;41(D1):991–5. 10.1093/nar/gks1193 PMC3531084 23193258

[92] Leinonen R, Sugawara H, Shumway M The Sequence Read Archive. Nucleic Acids Res. 2011;39(Suppl 1):2010–2. 10.1093/nar/gkq1019 PMC3013647 21062823

[93] Mashima J, Kodama Y, Fujisawa T, et al. DNA Data Bank of Japan. Nucleic Acids Res. 2017;45(D1):D25–D31. 27924010 10.1093/nar/gkw1001 PMC5210514

[94] Cook CE, Bergman MT, Cochrane G, et al. The European Bioinformatics Institute in 2017: Data coordination and integration. Nucleic Acids Res. 2018;46(D1):D21–D29. 29186510 10.1093/nar/gkx1154 PMC5753251

[95] Choi J, Pacheco CM, Mosbergen R, et al. Stemformatics: Visualize and download curated stem cell data. Nucleic Acids Res. 2019;47(D1):D841–D846. 30407577 10.1093/nar/gky1064 PMC6323943

[96] Li H, Durbin R Fast and accurate short read alignment with Burrows-Wheeler transform. Bioinformatics. 2009;25(14):1754–60. 19451168 10.1093/bioinformatics/btp324 PMC2705234

[97] Di Tommaso P, Moretti S, Xenarios I, et al. T-Coffee: A web server for the multiple sequence alignment of protein and RNA sequences using structural information and homology extension. Nucleic Acids Res. 2011;39(Suppl 2):13–7. 10.1093/nar/gkr245 PMC3125728 21558174

[98] Bankevich A, Nurk S, Antipov D, et al. SPAdes: A new genome assembly algorithm and its applications to single-cell sequencing. J Comput Biol. 2012;19(5):455–77. 22506599 10.1089/cmb.2012.0021 PMC3342519

[99] Papageorgiou L, Eleni P, Raftopoulou S, et al. Genomic big data hitting the storage bottleneck. EMBnet J. 2018;24:e910. 29782620 PMC5958914

[100] Jimenez RC, Kuzak M, Alhamdoosh M, et al. Four simple recommendations to encourage best practices in research software. F1000Res. 2017;6, doi:10.12688/f1000research.11407.1. PMC5490478 28751965

[101] R Core Team. R: A language and environment for statistical computing. Vienna, Austria: R Foundation for Statistical Computing; 2018 https://www.r-project.org/.Accessed on 23 December 2019

[102] Gentleman RC, Carey VJ, Bates DM, et al. Bioconductor: Open software development for computational biology and bioinformatics. Genome Biol. 2004;5(10):R80. 15461798 10.1186/gb-2004-5-10-r80 PMC545600

[103] Huber W, Carey VJ, Gentleman R, et al. Orchestrating high-throughput genomic analysis with Bioconductor. Nat Methods. 2015;12(2):115–21. 25633503 10.1038/nmeth.3252 PMC4509590

[104] Ushey K, McPherson J, Cheng J, et al. packrat: A dependency management system for projects and their R package dependencies. 2018 https://github.com/rstudio/packrat/.

[105] Anaconda Software Distribution. Anaconda. 2016 https://anaconda.com.

[106] Merkel D Docker: Lightweight Linux containers for consistent development and deployment. Linux J. 2014;239(2):1.

[107] Kurtzer GM, Sochat V, Bauer MW Singularity: Scientific containers for mobility of compute. PLoS One. 2017;12(5):e0177459. 28494014 10.1371/journal.pone.0177459 PMC5426675

[108] Bailey TL, Williams N, Misleh C, et al. MEME: Discovering and analyzing DNA and protein sequence motifs. Nucleic Acids Res. 2006;34(Web Server Issue):369–73. 10.1093/nar/gkl198 PMC1538909 16845028

[109] Xiong Y, Liu Y, Cao L, et al. Transcriptomic characteristics of bronchoalveolar lavage fluid and peripheral blood mononuclear cells in COVID-19 patients. Emerg Microbes Infect. 2020;9(1):761–70. 32228226 10.1080/22221751.2020.1747363 PMC7170362

[110] Wu F, Zhao S, Yu B, et al. A new coronavirus associated with human respiratory disease in China. Nature. 2020;579(7798):265–9. 32015508 10.1038/s41586-020-2008-3 PMC7094943

[111] Zhou P, Yang XL, Wang XG, et al. A pneumonia outbreak associated with a new coronavirus of probable bat origin. Nature. 2020;579:270–3. 32015507 10.1038/s41586-020-2012-7 PMC7095418

[112] Mussolino C, Alzubi J, Pennucci V, et al. Genome and epigenome editing to treat disorders of the hematopoietic system. Hum Gene Ther. 2017;28(11):1105–15. 28806883 10.1089/hum.2017.149

[113] Roberti A, Valdes AF, Torrecillas R, et al. Epigenetics in cancer therapy and nanomedicine. Clin Epigenetics. 2019;11(1):81. 31097014 10.1186/s13148-019-0675-4 PMC6524244

[114] Jinek M, Chylinski K, Fonfara I, et al. A programmable dual-RNA–guided DNA endonuclease in adaptive bacterial immunity. Science. 2012;337:816–22. 22745249 10.1126/science.1225829 PMC6286148

[115] Bardin P, Sonneville F, Corvol H, et al. Emerging microRNA therapeutic approaches for cystic fibrosis. Front Pharmacol. 2018;9:1113. 30349480 10.3389/fphar.2018.01113 PMC6186820

[116] Peter CJ, Saito A, Hasegawa Y, et al. In vivo epigenetic editing of Sema6a promoter reverses transcallosal dysconnectivity caused by C11orf46/Arl14ep risk gene. Nat Commun. 2019;10:4112. 31511512 10.1038/s41467-019-12013-y PMC6739341

