# Supplementary material for: Integrative computational epigenomics to build data-driven gene regulation hypotheses
Source: Gigascience. 2020 Jun 16;9(6):giaa064. doi: 10.1093/gigascience/giaa064 (PMC7297091; doi:10.1093/gigascience/giaa064)
Supplement: giaa064_GIGA-D-20-00089_Revision_1 [file giaa064_giga-d-20-00089_revision_1.pdf]

GigaScience

# Integrative Computational Epigenomics to build Data-driven Gene Regulation Hypotheses

--Manuscript Draft--

| | |
|---|---|
| **Manuscript Number:** | GIGA-D-20-00089R1 |
| **Full Title:** | Integrative Computational Epigenomics to build Data-driven Gene Regulation Hypotheses |
| **Article Type:** | Review |
| **Funding Information:** | Faculty of Science, Monash University — Mr Tyrone Chen<br>Dr Sonika Tyagi |
| **Abstract:** | Background: Diseases are complex phenotypes often arising as an emergent property of a non-linear network of genetic and epigenetic interactions. To translate this resulting state into a causal relationship with a subset of regulatory features, many experiments deploy an array of laboratory assays from multiple modalities. Often, each of these resulting datasets is large, heterogeneous and noisy. Thus, it is non-trivial to unify these complex datasets into an interpretable phenotype. Although recent methods address this problem with varying degrees of success, they are constrained by their scopes or limitations. Therefore, a significant gap in the field is the lack of a universal data harmoniser with the capability to arbitrarily integrate multi-modal datasets. Results : In this review, we perform a critical analysis of methods with the explicit aim of harmonising data, as opposed to case-specific integration. This revealed that matrix factorisation, latent variable analysis and deep learning are potent strategies. Finally, we describe the properties of an ideal universal data harmonisation framework. Conclusions : A sufficiently advanced universal harmoniser has major medical implications, such as 1). Identifying dysregulated biological pathways responsible for a disease as a powerful diagnostic tool 2) Investigating these pathways further allows the biological community to better understand a disease's mechanisms. 3) Precision medicine also benefits from developments in this area, particularly in the context of the growing field of selective epigenome editing, which can suppress or induce a desired phenotype. |
| **Corresponding Author:** | Sonika Tyagi<br>MONASH UNIVERSITY<br>Melbourne, VIC AUSTRALIA |
| **Corresponding Author Secondary Information:** | |
| **Corresponding Author's Institution:** | MONASH UNIVERSITY |
| **Corresponding Author's Secondary Institution:** | |
| **First Author:** | Tyrone Chen |
| **First Author Secondary Information:** | |
| **Order of Authors:** | Tyrone Chen |
| | Sonika Tyagi |
| **Order of Authors Secondary Information:** | |
| **Response to Reviewers:** | Dear Dr Nogoy<br>We are very thankful to the reviewers for their time and critical comments and supportive feedback on our article. We have addressed all the questions below and our responses are highlighted in blue. We have also made necessary changes in the manuscript and edits are highlighted in yellow.<br><br>Reviewer reports:<br>Reviewer #1: The authors review modern approaches for the integrative analysis of several datasets. I think that the authors provide a good overview of recent methods |

and the provided tables with classification of supported data types will be useful.
I have a few comments that the authors should address
Comments:
Fig. 1: I would not call it methylation marks. This may be confused with histone methylation, as these are often referred to as histone marks. Instead I would subtitle Methyl-Seq simply with DNA methylation.
Along those lines, it would be nice if the authors could cite a more recent paper about genome-wide methylation sequencing, not only the classical one from 1992, where the basic method was developed but not applied genome-wide.
Response: [Figure 1] To unambiguously refer to DNA methylation we changed "Methylation marks" to "DNA methylation".
Response: [line 26] To refer to genome wide methylation we now also cited Meissner et al 2005 on RRBS and the following 2008 Meissner et al application of the method to the whole genome.
Response: [Figure 1] Corrected a typo where we referred to RIP-seq as capturing DNA:RNA interactions instead of RNA:protein interactions.
Fig. 2: The distinct highlighting of measured regions for FAIRE and DNase-seq compared to ATAC-seq is misleading. In principle all of these assays will capture, nucleosome free regions, promoters and gene body. Of course each of these assays, depending on how the exact experimental setup is, will show enrichment in regions that other assays may miss (see a recent comparison we did for example: Unique and assay specific features of NOMe-, ATAC- and DNase I-seq data, NAR 2019).
Response: [Fig 2] Correctly aligned readout of FAIRE, DNaseI-seq, ATAC-seq on tracks.
Response: [Fig 2] In figure legends, we noted that each assay may have better performance depending on the experimental design and region targeted, with a reference.
I would recommend to refer to it as DNaseI-seq, to make clear which Dnase is being used.
Response: [Fig 2] Changed DNase-Seq to DNaseI-Seq to avoid ambiguity.
Also why do all three assays cover the complete gene body? In fact there is no reason to expect that these would cover anything except nucleosome-free regions in gene bodies, which would be intragenic enhancers most often in introns of genes. Other than that only at the promoter one normally sees strong signals.
Response: [Fig 2] We originally oversimplified this and have now partitioned gene regions for clarity.
Furthermore, I find the marking for methyl-seq also confusing. What is meant by this? Bisulfite sequencing for example covers the whole genome, but only Cytosine methylation is captured. In their current picture this is not clear.
Response: [Fig 2] For DNA methylation, we originally intended to indicate that this hypothetical genome had a methylation mark only at this location, which bisulfite sequencing captured. This explains the empty coverage at non-methylated regions. We now clarified this in figure legends, and explained that bisulfite sequencing normally covers the whole genome.
Fig. 3: it would be nice to indicate in the figure what the difference between inter and intra modality means. It may not be obvious to everyone as these terms are not often used, particular not concering the two different categories of inter-modality. They should also be clearly defined in the text.
Response: [193-204] Defined inter and intra modality in text (Existing data integration approaches).
Response: [Fig 3] Reworked figure layout and colour coded methods.
Why do they say that 4 approaches are common, when 5 are shown in the last row of Figure 3?
Response: [Fig 3] Now correctly stated that 5 approaches are shown in legends. We initially grouped latent and multivariate variables into the same superfamily of methods.
I don't like the arrangement of these 5 approaches in the figure. It is not clear if these are universal or belong to one of the types. I would include something like: Common approaches and then give them numbers from 1 to 5. Or maybe split them according to the use in restricted categories or generic categories?
Response: [Fig 3] To show approach membership, reworked figure layout and colour coded methods
In the figure caption they mention that the 5 approaches are used for inter modality approaches and only mention linear models for intra-modality. I cannot relate to this, in particular in the context of single cell analysis all of the 5 approaches have been

Powered by Editorial Manager® and ProduXion Manager® from Aries Systems Corporation

| | |
|---|---|
| | suggested for intra-modality analysis I believe. I think this distinction should be removed.<br>Response: [Fig 3] Removed distinction, merged conceptually similar approaches used by functionally different methods by colour coding<br>Also what does the black circle with ruler and pen mean?<br>Response: [Fig 3] Intended to represent custom workflows, replaced with text "Custom workflow".<br>-When the authors talk about Ensembl clustering (page 4 top, right column), I think they should really say ensembl learning. For example the first reference EPIP that they cite after their statement, does not use Random forest clustering, but another type of ensembl classifiers.<br>Response: [222-223] Corrected this statement to align with the definition of ensemble clustering (combine clusters) and ensemble learners (combine models).<br>Minor:<br>-Page 3. This sentence: "These include three-dimensional chromosome structure as measured with Hi-C [4], chromatin occu- pancy probed by ATAC-Seq [2] or ChIP-Seq [3], .." is misleading. Obviously ATAC-seq and ChIP-seq do no not measure the 3d-chromatin structure. If anything the 3d-chromatin structure may be inferred from those assays using additional information, but it seems far fetched in the way currently written.<br>Response: [120-123] Our original wording was unclear, now reworded to avoid the implication that ATAC-seq and ChIP-seq can be used to infer 3D structure.<br>I would like to point the authors to the zeroSum approach, which is a nice example of intra-modality correction for measurements on different platforms (Molecular signatures that can be transferred across different omics platforms, https://www.ncbi.nlm.nih.gov/pubmed/28881975).<br>Response: [Table 3] Reviewed zeroSum method and added it to the list, fixed a typo in our original table<br>Table 2: The caption is too small to be read without zooming in the pdf.<br>Response: [Table 1-3] Recoded small tables to be more human-readable.<br>Table 4/5: It would be good to include the citation to the methods in the table. For example the column "Chromatin occupancy and histone marks" could be called: "Epigenetic peak data" to shorten the text.<br>Response: [Table 4-5] Added citation to methods, renamed columns to optimise space.<br>In this context I would like to point out that sometimes a method may also include peaks from DNA methylation analysis, which is not entirely clear from the table as there is another column for DNA methylation. But I assume that the column DNA methylation refers to the fact that a method is able to handle the beta values explicitely?<br>Response: [Table 4-5] Clarified DNA methylation context in table legends<br>Following the above changes we have also simplified wordings in the conclusion section.<br><br>We hope that you find the changes satisfactory and we look forward to hearing from you soon.<br>With regards<br>Sonika Tyagi and Tyrone Chen |
| **Additional Information:** | |
| **Question** | **Response** |
| Are you submitting this manuscript to a special series or article collection? | No |
| **Experimental design and statistics**<br><br>Full details of the experimental design and statistical methods used should be given in the Methods section, as detailed in our Minimum Standards Reporting Checklist. Information essential to interpreting the | Yes |

Powered by Editorial Manager® and ProduXion Manager® from Aries Systems Corporation

| | |
|---|---|
| data presented should be made available in the figure legends.<br><br>Have you included all the information requested in your manuscript? | |
| **Resources**<br><br>A description of all resources used, including antibodies, cell lines, animals and software tools, with enough information to allow them to be uniquely identified, should be included in the Methods section. Authors are strongly encouraged to cite Research Resource Identifiers (RRIDs) for antibodies, model organisms and tools, where possible.<br><br>Have you included the information requested as detailed in our Minimum Standards Reporting Checklist? | Yes |
| **Availability of data and materials**<br><br>All datasets and code on which the conclusions of the paper rely must be either included in your submission or deposited in publicly available repositories (where available and ethically appropriate), referencing such data using a unique identifier in the references and in the “Availability of Data and Materials” section of your manuscript.<br><br>Have you have met the above requirement as detailed in our Minimum Standards Reporting Checklist? | Yes |

Revised Manuscript

Click here to access/download;Manuscript;manuscript_gigascience_revised.

*GigaScience*, 2020, 1–15

doi: xx.xxxx/xxxx
**Manuscript in Preparation**
**Paper**

PAPER

# Integrative Computational Epigenomics to build Data-driven Gene Regulation Hypotheses

Tyrone Chen[1] and Sonika Tyagi[1]

[1]School of Biological Sciences, Monash University, Clayton, VIC 3800, Australia

*sonika.tyagi@monash.edu

## Abstract

**Background**: Diseases are complex phenotypes often arising as an emergent property of a non-linear network of genetic and epigenetic interactions. To translate this resulting state into a causal relationship with a subset of regulatory features, many experiments deploy an array of laboratory assays from multiple modalities. Often, each of these resulting datasets is large, heterogeneous and noisy. Thus, it is non-trivial to unify these complex datasets into an interpretable phenotype. Although recent methods address this problem with varying degrees of success, they are constrained by their scopes or limitations. Therefore, a significant gap in the field is the lack of a universal data harmoniser with the capability to arbitrarily integrate multi-modal datasets. **Results**: In this review, we perform a critical analysis of methods with the explicit aim of harmonising data, as opposed to case-specific integration. This revealed that matrix factorisation, latent variable analysis and deep learning are potent strategies. Finally, we describe the properties of an ideal universal data harmonisation framework. **Conclusions**: A sufficiently advanced universal harmoniser has major medical implications, such as 1). Identifying dysregulated biological pathways responsible for a disease as a powerful diagnostic tool 2) Investigating these pathways further allows the biological community to better understand a disease's mechanisms. 3) Precision medicine also benefits from developments in this area, particularly in the context of the growing field of selective epigenome editing, which can suppress or induce a desired phenotype.

**Key words**: Bioinformatics, Computational Biology, Neural Networks, Data Integration, Deep learning, Epigenetics, Epigenomics, Gene Regulation, Genomics, High Throughput Sequencing, Machine Learning

## Background

### Importance of data harmonisation

Answers to gene regulation of disease and normal development lie encrypted in the epigenome. In this context, we define the epigenome as the chromatin state map of the genome and other gene expression-controlling factors [Figure 1]. To capture this state, we require genome-wide measurement of combinations of epigenetic marks occurring in different cell types under various conditions [Figure 2]. Epigenetic systems contributing to this resulting epigenomic state are highly complex, and are often the result of multi-layered and combinatorial interactions between different regulatory components of an epigenome [2]. In addition, these interactions are highly dynamic and can vary under different conditions. Therefore, any individual omics assay or data modality results in an incomplete view of a biological system. Recently, the community is moving towards adopting data-driven approaches to determine gene-specific regulatory pathways of complex phenotypes in cases such as disease progression. This is due to the increasing availability of large scale high throughput epigenomic datasets. Therefore, coherently integrating and identifying gene regulatory information across multiple datasets, especially across different types of omics experiments as well as data modalities (such as ATAC-Seq (assay for transposase-accessible chromatin) [3], ChIP-Seq (chromatin immunoprecipitation) [4], Hi-C (high throughput chromosome conformation capture) [5], Methyl-Seq [6, 7, 8], RIP-Seq (RNA immunoprecipitation) [9] as bulk or single cell sequencing data) is now an essential and chal-

**Compiled on:** May 25, 2020.
Draft manuscript prepared by the author.

## Key Points

- Many biological assays and computational models to study gene regulation focus on analysing individual data modalities in isolation.
- To understand the causal relationship between complex disease phenotypes and epigenomics, data harmonisation across different data modalities is necessary.
- **Data harmonisation** is defined as the unification of low-level features across different data modalities, distinct from the ambiguous definition of data integration.
- Machine learning, dimensionality reduction, matrix factorisation, latent variable approaches and mutual nearest-neighbour strategies are some emerging methods which address the problem of harmonising data.

**Figure 1.** Genomic features affecting gene regulation are shown, along with the corresponding assays used to infer the state of the regulatory feature. We note that this is not an exhaustive list of assays available to profile regulatory features.

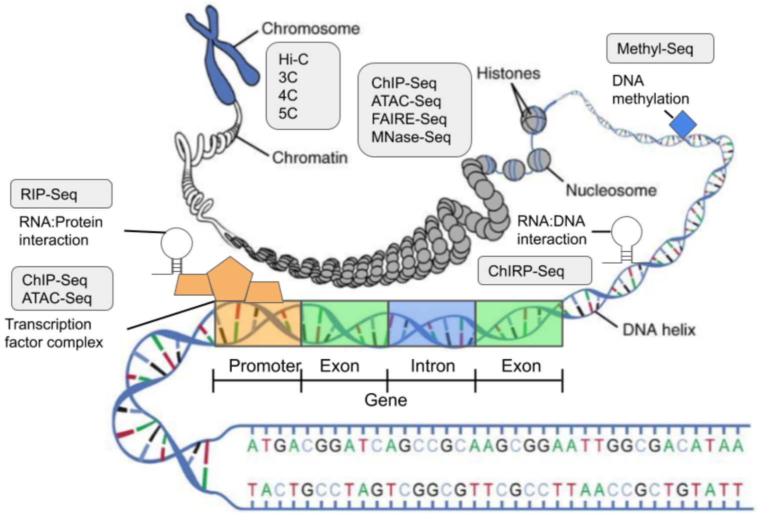


**Figure 2.** Information returned by types of functional assays targeting RNA-DNA interactions, RNA-protein interactions (including histones), quantifying RNA abundance, DNA-DNA interactions, DNA-protein interactions (including histones) and direct biochemical modifications to DNA. By probing the association of DNA with these regulatory factors, we can observe the activity fingerprint of a genome. Combined with functional outcome information such as gene expression, we can infer the flow of signals which result in a phenotype. (Methyl-Seq normally covers the entire genome, but for simplicity we show coverage at a single methylated site in the hypothetical genome. We note that the performance of some of these assays may vary depending on the experimental design and region targeted [1].)

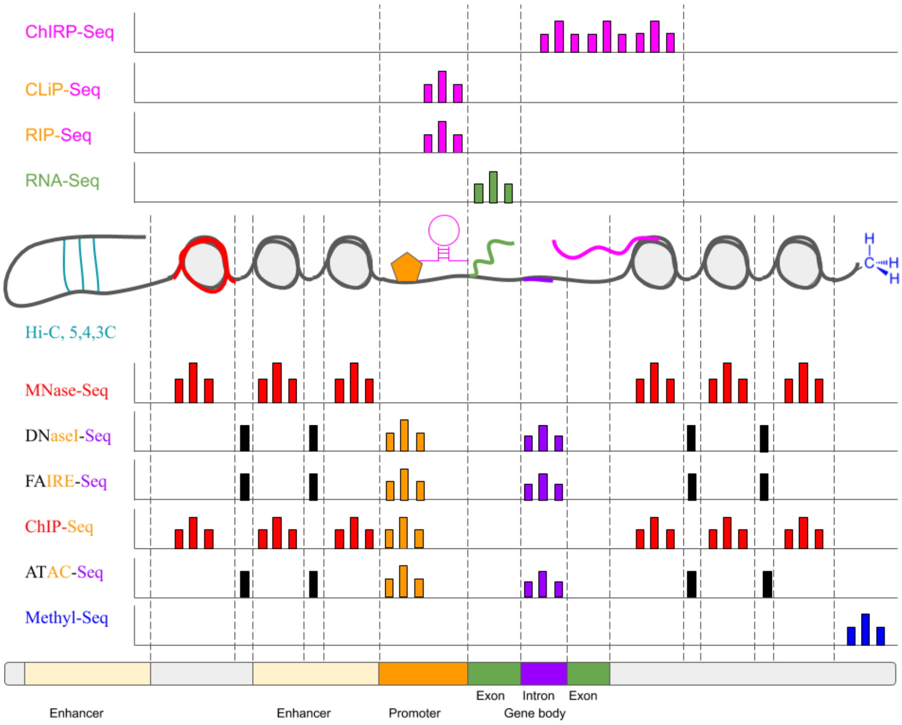

lenging task [Figure 1]. Current attempts are usually limited to a restricted set of modalities, and there are many methods which appear to be integrative at a first glance but upon closer investigation have different scopes [10, 11, 12, 13, 14]. Unfortunately, no method currently meets this need of capturing a complete cell or tissue state in an accurate and comprehensive way.

Later in this review, we will discuss in detail opportunities involved in harmonising different types of omics data within and across experiments to unlock deeper layers of information present within a biological process involved in disease or complex traits. This holistic genomics approach has been recently gaining momentum [15, 16, 17, 18, 19]. Simultaneously harmonising data, for example gene expression with chromatin accessibility provides an extra layer of validation for the obtained results and reduces false discovery rate while increasing reproducibility. The user will have a higher degree of confidence in the results due to their concordance on separate data categories. Together, these complementary methods provide a higher resolution view into the data, improving our understanding of epigenetic mechanisms of complex diseases as well as traits and subsequently enabling new treatment opportunities.

### *Challenges in data harmonisation*

Although multi-modal data integration appears very attractive, it comes with exponential technical, statistical and computational challenges. For example, 1) biological data is generated from a wide range of dimensions and from an equally large variety of sources. Dataset heterogeneity results in significant computational issues during analysis, as technical artefacts, dataset complexity and small sample sizes all contribute to noise in the data. 2) Furthermore, domain-specific knowledge is required to interpret the results of computational tools, and this requirement is particularly important when considering the specific assumptions that these tools usually make. 3) Analysing data from even a single modality, whether from a single experiment or multiple experiments is cumbersome, as thousands of genes can now be assayed in parallel, generating an equal number of hypotheses. In the case of analysing multimodal data, this challenge is amplified by the non-linear relationship between different omics datasets.

### *Strategies to harmonise data*

Integrating information from different resources in a single, unified view can be performed in a variety of ways. While many existing workflows do not explicitly model data modality harmonisation, they nevertheless use information from different layers of omics data. Integrating this data can happen at different stages of data processing: primary, intermediate states or fully processed data. Commonly, integration happens at the final step by repeatedly summarising primary data from each modality and collapsing them into gene lists, removing a significant amount of quantitative and other forms of valuable information.

This need for a greater level of biological understanding has given rise to methods which attempt to take a more holistic, inter-omics approach, in contrast to the reductionist approach where hypotheses are modular. These fall into two broad categories, 1) targeted data integration (inter-modality restricted), and 2) general data harmonisation (inter-modality free) [Figure 3]. Targeted data integration focuses on integrating two or three specific data modalities with clear correlations, for example, the relationship between chromatin occupancy and transcription. Recently, more agnostic data harmonisation methods aiming to unify information from an arbitrary number of categories are emerging [20, 21]. In the latter case, there are few such methods available due to the previously discussed challenges of unifying different data modalities and the relative novelty of this class of approaches. In the rest of the article we will look at different types of epigenomic regulatory features, epigenomic data available for regulatory feature detection and computational methods for building correlation-to-causal gene regulation hypothesis. A critical comprehensive review of these methods has not been previously attempted.

### *Epigenetic regulatory features driving gene activity*

Before reviewing these methods, it is first necessary to consider the breadth and mechanisms of epigenetic regulatory features present in the genome. We show that two common themes exist among these regulatory features. First, they induce a transcriptionally permissive or repressive environment by altering steric hindrance in DNA towards other regulatory elements. Second, regulatory features rarely act alone, and a signal transduction cascade mediated by multiple regulatory elements is necessary to shape a cell state [22] [see Additional file 1: Figure S1]. Each feature is highly nuanced, and are not always directly correlated, making direct comparisons difficult.

Gene expression is a tightly modulated process, and perturbations at any step can have negative consequences. Many human diseases are associated with dysregulation of gene expression, including many cancers [23]. Understanding this epigenomic regulatory machinery is therefore crucial to both understanding the biology of any system and applying this information to treat human diseases. Known epigenetic regulatory features are diverse, but can be classified into several general categories. At a high level, three-dimensional chromosome structure can be measured with Hi-C [5]. ATAC-Seq captures nucleosome occupancy to reveal the accessibility state of chromatin (open or closed) [3] whereas ChIP-Seq [4] and RIP-Seq [24] capture DNA-protein and RNA-protein binding sites, respectively. Both can probe the occupancy of nucleic acid by regulatory proteins.

It is also possible to capture interactions between as well as within nucleic acids and proteins by other immunoprecipitation techniques like RIP-Seq [9], along with direct biochemical modifications such as methylation to any of these [6, 7, 8]. A detailed list of epigenomic features and laboratory assays to study them are provided in [see Additional file 1: Table S1].

Many of these features alter the level of steric hindrance in DNA towards other regulatory features. Disruptive DNA loops, chromatin formation and DNA methylation prevent gene transcription by physically excluding facilitative protein complexes from binding. Conversely, permissive DNA loops, chromatin free regions and transcription factors boost the probability of regulatory element binding events by removing or circumventing this barrier.

Although some epigenomic regulatory features have a direct impact on gene expression, gene regulation is often achieved through a web of cause and effect, of which there are abundant examples. Protein biochemical modifications such as histone methylation stabilises associated chromatin to strengthen transcriptional silencing while histone deacetylation has the opposite effect [25, 26, 27]. Cis-acting long non-coding RNA can act as a targeted scaffold to bind DNA and proteins to modulate transcriptional permissiveness, and are often associated with enhancers [28, 29]. (These are distinct from enhancer RNAs which are shorter and unstable [29]). In some cases, long non-coding RNAs can even encode functional proteins [30]. Other forms of RNA regulation are co-transcribed circular RNA and microRNA (miRNA) which control mRNA levels [31, 32, 33, 34]. lncRNA can act as an miRNA sponge thus working in a feedback loop to release or sequester RNA or even protein [35]. This effect extends to transcription factors in the case of lncRNAs GAS5 [36] and RMST [37]. Meanwhile, transcription factors can work in combination with each other as well as with activator and repressor proteins to further tune

gene expression levels [38]. Metabolite abundance levels in or around the cell may trigger signal cascades through the activation or deactivation of receptor proteins which result in an upregulation or downregulation of transcription.

After transcription, it is still possible for a cell to selectively calibrate transcript quantity. Trans-acting long non-coding RNA Stabiliser proteins and functionally equivalent RNA may bind to transcripts to increase their half-life in the cell, or tag them for degradation by enzymes [29]. Extending this ability, trans-acting circular RNA can sequester or release transcripts, acting as a RNA battery within the cell [39, 40, 31].

Genome sequence is by definition not epigenomic, but it is relevant to note that changes in DNA sequence which alter steric hindrance or three dimensional chromosome structure can directly impact the epigenome. An initial dysregulated binding event may trigger a chain reaction with significant positive or negative effects. At the same time, this highlights the limitations of a reference genome [41]. Despite being a powerful resource, it remains incomplete and is affected by both biological [42] and technical [43] variation just as with any other epigenetic data modality. Thus, informative variation within organisms even of the same species can be masked, decreasing inference accuracy.

## Outline

In this article, we will critically review methods which aim to resolve the issue of multi-modal data integration, and classify them into categories based on their scope. In each category as well as across the field as a whole, we identify common features shared by these strategies, and highlight those that show the most potential. At the same time, we suggest a list of epigenomic databases containing the previously discussed gene regulatory features for use by researchers interested in developing or refining such methods, and note the properties of these databases which may positively or negatively affect this process. From this analysis of strategies and databases, we then envision the attributes of an ideal universal data harmonisation framework, and demonstrate some powerful applications.

# Main Text

## Existing data integration approaches

Many attempts have been made to address the challenges of epigenomic data harmonisation both across and within modalities. These methods are diverse, and we developed a functional taxonomy for the methods surveyed. We filtered out data aggregation methods which group data for viewing, for example as Venn diagrams of gene lists, and do not directly integrate data. We then classified integrative methods into two categories: intra-modality and inter-modality approaches. Intra modality approaches focus on removing technical variation caused by batch effects, while inter modality approaches aim to combine different data modalities which may not be directly comparable. Inter-modality data harmonisation can be further subclassified into two categories, modality-restricted and modality-free [Figure 3]. The former has a highly restricted scope and is configured to work with a specific experimental design, while the latter is theoretically generalisable to any experimental design.

### *Inter-modality restricted*

Modality restricted approaches target a specific set of modalities, often applying modality-specific constraints or assumptions to two or three data modalities only [Table 1,4]. Often, they exploit the strong correlation present between certain data modalities, for instance in the case of relating chromatin occupancy to transcript expression in EPIP [44] and TEPIC [45]. Furthermore, many methods including LemonTree [46], ELMER [47] and PARADIGM [48] are configured specifically for or tested only on a target category of biological systems, in particular human cancers, sometimes to the extent of being less generalisable to other systems.

Among these modality-restricted methods, a common theme is the usage of ensemble learning, such as random forests [61], on quantitative omics data. Examples of such methods are EPIP [44], EAGLE [51], IM-PET [55], JEME [56] and RIPPLE [57]. Their popularity and success may be attributable to the nature of the algorithm, where an ensemble of clusters representing independent biological signals results in convergence even in heterogeneous data. Notably, these methods appear to be more generalisable than the other methods reviewed [Table 1]. Another group of methods approach the problem from a different perspective by applying network analysis to leverage biomolecular interaction information instead of molecule abundance. These methods include LemonTree [46], PANDA [54] and PARADIGM [48]. Due to the unique angles of each method, formulations of the problem and applications of the strategy, the advantages and disadvantages of each method vary significantly. These are evaluated in detail in [Table 1]. One method worth highlighting is RIMBANET [50], which is interesting due to its ability to integrate data from six different modalities including proteomics and metabolomics data, but it is important to note that it was tailored to a specific experiment.

### *Inter-modality free*

In contrast to modality-restricted approaches, modality-free methods [Table 2,5] are omics-agnostic, to the point of accepting medical imaging data in a few cases. Many of these methods, such as DIABLO [20], iCluster [62], GFA [63] and MOFA [64] use latent variable analysis and others like NMF [65], iNMF [66] and LIGER [67] use non-negative matrix factorisation to harmonise multi-omics data [Fig 3, Table 2]. These categories of methods are particularly viable and flexible since any data can be ingested as long as they can be represented as a generic matrix of values [Figure 3]. DIABLO [20] in particular stands out as a method which was successfully applied to four categories of multimodal data, including proteomics and metabolomics. We compare and contrast these methods in more detail in [Table 2]. At least one recent method has successfully combined non-negative matrix factorisation with deep learning [21], and this trend of coupling of deep learning to conventional integration strategies is expected to continue given deep learning's applicability in deconvoluting non-linear relationships in large datasets [68]. We also note that two single-cell methods, LIGER [67] as well as seurat [69] are present, and observe that bulk RNA-Seq methods and single-cell methods are mutually exclusive.

### *Intra-modality*

A special subset of data harmonisation approaches is focused on unifying intra-modality data, and more of these details are available in [Table 3]. This arose due to the common problem in biology of handling unwanted technical variation in data, which can be caused easily by performing experiments on different instruments or on different days [77]. While this class of methods is not directly associated with the broader problem of inter-modality data harmonisation, mathematical approaches used to address this problem tend to overlap, such as mutual nearest neighbours in mnnCorrect [78] and seurat [69] [Fig S2]. It is highly likely that we can exploit properties of relevant strategies and methods to achieve a better model of data har-

**Figure 3. (a)** A functional taxonomy of data integration methods is shown in the top half of the figure. Inter-modality and intra-modality harmonisation methods exist, along with data aggregation tools which are not data integrators in the context of our review. With inter-modality restricted methods, custom strategies are common. For inter-modality generic methods, five approaches are common, which are shown in the bottom half of the figure. **(b)** Mutual nearest neighbours exploits common points between single cell datasets as references, matrix factorisation operates on abundance measures to categorise data and is agnostic to data type, multivariate models attempt to account for dependent and independent variable contribution to the output, latent variable models attempt to model an unobserved factor's contribution to the output and deep learning optimises a series of regressions to yield a categorical variable or generate an output. Intra-modality harmonisation methods share these strategies but apply them specifically to reduce unwanted technical variation. We note that current methods used data in a processed state and for a better harmonisation raw or intermediate data (as shown by the dotted lines) can be used.

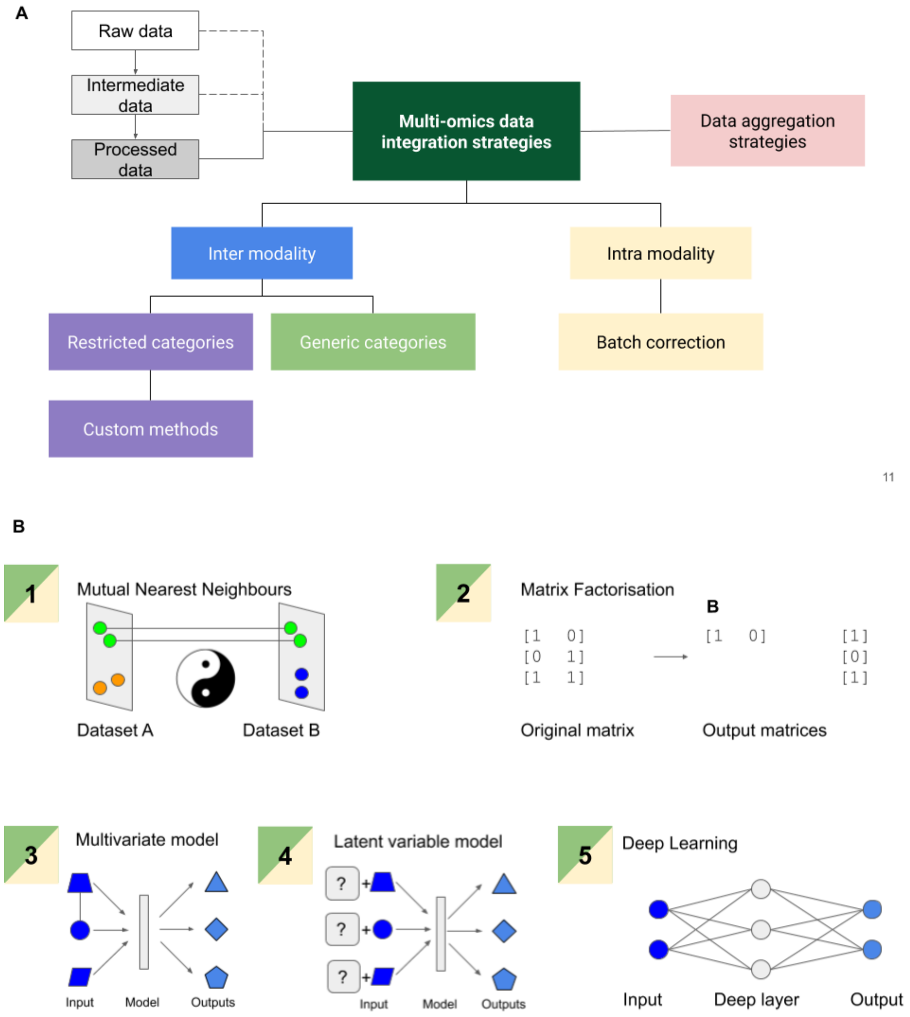

**Table 1.** Inter-modality data harmonisation approaches with a restricted modality scope. Names, strategies, advantages and limitations of each method is provided. Regarding advantages and limitations, a few major points were highlighted, and it is important to note that many of these methods are highly nuanced. A citation for reference to the original manuscript of each method is provided where full details can be obtained.

| Method name | Strategy | Main advantages | Main limitations | Citation |
|---|---|---|---|---|
| MDI | Bayesian Consensus Clustering | Identifies gene clusters across datasets with specific shared characteristics. Can model time series data | Limited to querying a small subset of genes. Trained only on array data | [49] |
| RIMBANET | Bayesian MCMC | Integrates many data types simultaneously | Requires large quantities of multimodal data. Method was specifically designed for experiment | [50] |
| EPIP | Ensemble boosting | Effective in unbalanced datasets | Limitations of training data reduces model effectiveness in small datasets | [44] |
| EAGLE | Ensemble boosting | Uses higher-level features to buffer against overfitting | Custom genome-specific features need to be calculated for classification | [51] |
| PreSTIGE | Information theory | Outputs different specificity thresholds | Biased to cell type | [52] |
| TEPIC | Machine learning | Feature space improves result interpretability | Limited performance in gene dense regions or with small sample sizes | [45] |
| iOmicsPASS | Network analysis | Produces a sparse set of easily interpretable biological interactions Effective in heterogeneous datasets | Important markers that are poorly represented in biological networks can be lost in the analysis | [53] |
| LemonTree | Network analysis. Gibbs sampler. Decision tree | Modular model parts for different cases | Trained on cancer data | [46] |
| PANDA | Network analysis. Message passing | Accounts for lack of direct regulatory element interaction | Choice of convergence parameter affects results. Results may be difficult to interpret | [54] |
| PARADIGM | Network analysis. Probabilistic Graph Model | Robust to false positives | Training was performed on microarray data. Effectiveness in seq data unknown. Trained on cancer data | [48] |
| IM-PET | Random Forest classifier | Expected to generalise to other species | Requires assembly of 4 manually derived scores | [55] |
| JEME | Random Forest classifier. Regression | Easily re-trainable on different systems if sufficient data is available | At least 4 input data types are required | [56] |
| RIPPLE | Random Forest classifier. Regression | Generalisable to other biological conditions and cell types | Assumes balanced data categories | [57] |
| SVM-MAP | Support Vector Machine | Expected to generalise to multiple cancer types | Limited enhancer coverage in training data | [58] |
| ELMER | Wilcoxon rank-sum test | Identifies upstream master regulators | Restricted to methylation arrays in cancer | [47] |
| TENET | Wilcoxon rank-sum test | Expected to generalise to other biological systems | Targets group expression differences only | [59] |
| RegNetDriver | Wilcoxon rank-sum test | Provides a framework to construct tissue-specific regulatory networks | Requires assembly of multiple manually derived scores from system-specific steps | [60] |

monisation, for example by adding mutual nearest neighbours as an intermediate or refining step to the existing combination of latent variable analysis, matrix factorisation and deep learning.

### *Common themes across all methods*

Five interesting patterns emerge from this aggregated comparison of methods [Table 4, 5]. First, gene expression data appears to be universal to all approaches, and is often assigned a bridging role across the data modalities. This is likely due to its direct correlation to gene activity, as well as its relative interpretability compared to other omics data, being a straightforward readout of gene activity. Second, quantitative omics data such as gene expression and miRNA are often easier to merge since all data involved is continuous and representable as matrices as opposed to qualitative omics data such as chromosome conformation or chromatin accessibility. Thus, methods merging quantitative omics data such as DIABLO [20] are more generalisable than those merging qualitative and quantitative omics data. Third, data harmonisation can be performed at any stage, but is commonly performed as a final step with fully processed data. Fourth, single-cell multi-omics harmonisation approaches are separate from bulk-cell multi-omics harmonisation approaches. This is mainly attributable to the distinct statistical properties between single-cell and bulk cell data, which is not straightforward to reconcile. Fifth, it is not

**Table 2.** Inter-modality data harmonisation approaches with a free modality scope. Note that seurat and LIGER are specific to single cell data and the others are intended for bulk data. Names, strategies, advantages and limitations of each method is provided. Regarding advantages and limitations, a few major points were highlighted. A citation for reference to the original manuscript of each method is provided where full details can be obtained.

| Method name | Strategy | Main advantages | Main limitations | Citation |
|---|---|---|---|---|
| DeepMF | Deep learning and Non-negative Matrix Factorization | Robust to noise and missing data | Manual parameter tuning and prior information may be required | [70] |
| JIVE | Dimensionality reduction | Identifies the global modes of variation that drive associations across and within data types | Not robust to outliers or missing values or class imbalance | [71] |
| GCCA | Generalized canonical correlation analysis | Identifies blocks of variables within datasets for correlation across datasets | Less effective if the number of observations is smaller than the number of variables or if multiple linear correlations are present between datasets. Biases towards strong variation in the data | [72] |
| NetICS | Graph diffusion | Robust to frequency of aberrant genes in sample | Can only examine effects of known genes present in a defined interaction network | [73] |
| DIABLO | Multivariate model and Latent variable model | Captures quantitative information. Visual outputs aid interpretation | Assumes a linear relationship between the selected omics features. Parameter tuning is required | [20] |
| iCluster | Latent variable model | Captures both concordant and unique alterations across data types | Sensitive to initial subset selection. Trained only on array data | [62] |
| GFA | Latent variable model | Accepts data with missing values | Manual parameter tuning. Prior information may be required | [63] |
| MOFA | Latent variable model and Probabilistic Bayesian | Leverages multiomics to impute missing values. Single cell version available | Assumes a linear relationship between the selected omics features Manual parameter tuning required | [74] |
| seurat | Mutual nearest neighbours | Effective in intra-modality as well as inter-modality integration. robust to parameter changes | Restricted to single cell Requires robust reference data | [69] |
| SNF | Network analysis | Effective in small heterogeneous samples. Captures quantitative information | Does not yield quantitative data Trained only on array data | [75] |
| NMF | Non-negative Matrix Factorization | Accounts for complex modular structures in multimodal data | Trained only on array data | [65] |
| iNMF | Non-negative Matrix Factorization | Stable even in heterogeneous conditions | Trained only on array data | [66] |
| LIGER | Non-negative Matrix Factorization | Effective in intra-modality as well as inter-modality integration effective in highly divergent datasets | Restricted to single cell | [67] |
| sMBPLS | Sparse Multi-Block Partial Least Squares regression | Derives weights for modalities indicating contributions to expression | Performance is reduced with lower data dimensions | [76] |

uncommon to observe combinations of strategies within a single method, for example the coupling of network analysis to decision trees or regression. The main reason for this is that the complementary nature of different methods working in tandem usually results in a higher resolution view of a dataset.

We also emphasise the fact that while some methods may appear to be integrative at first glance, this may not necessarily be the case depending on the application and nuances of the method. For example, methods may combine different data modalities during method development but apply it specifically to signal detection in unimodal data. Deep learning-based methods such as Deepbind [10], BP-Net [12], EP-DNN [11], RE-VAE [13], x-CNN [14] and others commonly fall into this category, as models are often trained on multi-modal data but with a restricted goal of identifying motifs in nucleic acids.

**Table 3.** Intra-modality data harmonisation approaches. Batch is a special case of intra-modality harmonisation and is included for completeness, as many underlying strategies used are applicable to broader data integration. All methods are restricted to a single data modality of transcriptomics. Names, strategies, advantages and limitations of each method is provided. Regarding advantages and limitations, a few major points were highlighted. A citation for reference to the original manuscript of each method is provided where full details can be obtained.

| Method name | Strategy | Main advantages | Main limitations | Citation |
|---|---|---|---|---|
| ComBat | Bayesian empirical | Removes batch effect in most cases | Removes biological signal in most cases | [4] |
| RUV | Linear model | Effective with spike-in controls | Individual variants make specific assumptions about the data | [79] |
| removeBatchEffect | Linear model | Generalisable to most transcriptomic data types | May be less effective in complex experimental designs | [80] |
| SVN | Linear model | Generalisable to many cases | Assumes that feature similarities between datasets are due to biology | [81] |
| mnnCorrect | Mutual nearest neighbours | Accounts for heterogeneity within sample groups | Restricted to single cell data | [78] |
| MINT | Multivariate model | Robust to overfitting and strong multidimensional technical variation | Minimum sample count requirement | [82] |
| Scanorama | Mutual nearest neighbours | Scales to very large sample sizes. Robust to overcorrection | Restricted to single cell | [83] |
| MultiCluster | Tensor decomposition | Accounts for multiple batch variables simultaneously | Restricted to three-way variable comparisons | [84] |
| zeroSum | Zero sum regression | Generalisable across different technologies and platforms | Weak or non-linear features may be masked by strong features | [85] |

**Table 4.** Type and number of data modalities **supported** by each inter-modality data harmonisation approach (restricted modality scope). Quantity of compatible modalities are listed in the "Modalities compatible" column. "DNA methylation" in this context refers specifically to the ratio of signal between methylated and unmethylated alleles. For simplicity, some modalities have been aggregated, for example transcriptomics data includes both gene expression and small rna data. Some methods are capable of handling proteomics, metabolomics or medical images, but these are excluded as they are not a focus of this review. A link to each method is provided for easy reference.

| Method name | Modalities compatible | 3D chromosome structure | DNA methylation | Epigenetic peak data | DNA-Protein binding | DNA-RNA interactions | RNA-Protein interactions | Protein-Protein interactions | Genomics | Transcriptomics | Citation |
|---|---|---|---|---|---|---|---|---|---|---|---|
| MDI | 3 | X | X | O | X | X | X | O | X | O | [49] |
| RIMBANET | 4 | X | X | X | O | X | X | O | O | O | [50] |
| EPIP | 4 | O | X | O | O | X | X | X | X | O | [44] |
| EAGLE | 2 | X | X | X | O | X | X | X | X | O | [51] |
| PreSTIGE | 2 | X | X | O | X | X | X | X | X | O | [52] |
| TEPIC | 3 | O | X | O | X | X | X | X | X | O | [45] |
| iOmicsPASS | 2 | X | X | X | X | X | X | X | O | O | [53] |
| LemonTree | 2 | X | X | X | X | X | X | X | O | O | [46] |
| PANDA | 3 | X | X | X | O | X | X | O | X | O | [54] |
| PARADIGM | 2 | X | X | X | X | X | X | X | O | O | [48] |
| IM-PET | 2 | X | X | O | X | X | X | X | X | O | [55] |
| JEME | 2 | X | X | O | X | X | X | X | X | O | [56] |
| RIPPLE | 3 | X | X | O | O | X | X | X | X | O | [57] |
| SVM-MAP | 2 | X | O | X | X | X | X | X | X | O | [58] |
| ELMER | 2 | X | O | X | X | X | X | X | X | O | [47] |
| TENET | 2 | X | O | X | X | X | X | X | X | O | [59] |
| RegNetDriver | 5 | X | O | O | O | X | X | X | O | O | [60] |

**Table 5.** Type and number of data modalities **tested** by each inter-modality data harmonisation approach (free modality scope). Note that GCCA [72], seurat [69] and LIGER [67] are specific to single cell data and the others are intended for bulk data. Quantity of trained modalities are listed in the "Modalities trained" column. "DNA methylation" in this context refers specifically to the ratio of signal between methylated and unmethylated alleles. In contrast to Table 4A, the quantity of modalities represents the quantity of modalities which the algorithm was tested on and does not reflect the modalities which the algorithm is compatible with. For simplicity, some modalities have been aggregated, for example transcriptomics data includes both gene expression and small RNA data, which gives the illusion that DeepMF [21] and JIVE [71] were trained on unimodal data. Some methods are capable of handling proteomics, metabolomics or medical images, but these are excluded as they are not a focus of this review. A link to each method is provided for easy reference.

| Method name | Modalities trained | 3D chromosome structure | DNA methylation | Epigenetic peak data | DNA-Protein binding | DNA-RNA interactions | RNA-Protein interactions | Protein-Protein interactions | Genomics | Transcriptomics | Citation |
|---|---|---|---|---|---|---|---|---|---|---|---|
| DeepMF | 1 | X | X | X | X | X | X | X | X | O | [70] |
| JIVE | 1 | X | X | X | X | X | X | X | X | O | [71] |
| GCCA | 2 | X | X | X | X | X | X | X | O | O | [72] |
| NetICS | 3 | X | O | X | X | X | X | X | O | O | [73] |
| DIABLO | 2 | X | O | X | X | X | X | X | X | O | [20] |
| iCluster | 3 | X | O | X | X | X | X | X | O | O | [62] |
| GFA | 2 | X | O | X | X | X | X | X | X | O | [63] |
| MOFA | 2 | X | O | X | X | X | X | X | X | O | [74] |
| seurat* | 2 | X | X | O | X | X | X | X | X | O | [69] |
| SNF | 2 | X | O | X | X | X | X | X | X | O | [75] |
| NMF | 2 | X | O | X | X | X | X | X | X | O | [65] |
| iNMF | 2 | X | O | X | X | X | X | X | X | O | [66] |
| LIGER* | 2 | X | O | X | X | X | X | X | X | O | [67] |
| sMBPLS | 3 | X | O | X | X | X | X | X | O | O | [76] |

## Epigenomic data resources

Therefore, we demonstrate the requirement for a universal data harmoniser. However, before proposing and describing a suitable framework, we first need to discuss suitable training and validation data, as this is the most important component in any biological framework, and will be the main factor in the resulting viability of a method.

### *Data standardisation*

To develop appropriate models or methods, it is necessary to have a well-curated set of high quality epigenomic data [see Additional file 1: Figure S4B]. Currently there is a wealth of biological data available, but not all publicly accessible biological data is standardised or curated to the extent needed by some types of experiments. Standardised laboratory protocols and standardised software pipelines are necessary to limit the ef-

fect of unwanted technical variation in the data, which can contribute to significant noise in the data or lead to unintentionally flawed conclusions [77].

***Data accessibility***

Furthermore, databases can have specific scopes or restrict access to data, especially pertaining to sensitive patient information [see Additional file 1: Table S4B]. IHEC (International Human Epigenome Consortium) [86] and TCGA (The Cancer Genome Atlas) [87] are the primary examples of this [see Additional file 1: Table S2]. In such cases, users may be limited to non-primary data sources or a restricted subset of samples, which may yield sufficient information depending on the purposes and design of the integrative experiment.

***Data modality***

There is a wide variety of epigenomics data modalities present in each database [see Additional file 1: Table S4A]. The choice of database from which to obtain training data for a method should be made based on their individual scopes while taking data standardisation and accessibility into account. For example, users interested in human development or disease can select the ENCODE (Encyclopedia of DNA Elements) [88] or Roadmap [89] databases, as they contain relevant, standardised and publicly accessible curated datasets [see Additional file 1: Table S2]. In contrast, users seeking less common datasets associated with rare diseases may not necessarily find the information in a standardised or accessible database, and can broaden their search to include ArrayExpress [90], GEO (Gene Expression Omnibus) [91] or INSDC (International Nucleotide Sequence Database Collaboration) [92, 93, 94], which stores the data of independent experiments. Quality of data in such cases is not guaranteed, and this is best illustrated with a recent example showing that among several hundred stem cell datasets from these databases, a third were irreproducible due to inappropriate experimental design or sample mislabellings [95]. Care should be taken to detect and account for unwanted technical variation in such cases.

## Towards a universal data harmoniser

With such suitable data, we propose and outline the ideal universal data harmoniser, which would be agnostic to input omics type and scale to an arbitrary cardinality of modalities. In addition, it should be easy to use, yield interpretable results and be robust to noise [see Additional file 1: Table S3].

***Functionality***

A universal data harmoniser has to resolve the previously discussed challenges of distinct omics types and arbitrary cardinality. We re-emphasise the necessity of accounting for non-linear relationships across multimodal datasets. While many existing methods yield results by exploiting strong correlations between specific omics data, for example between gene expression and chromatin accessibility [55, 57, 56], this does not hold true in all cases. One scenario where this is particularly visible is the relationship between DNA methylation and chromosome conformation, where simple linear correlation is unlikely to be effective in predicting a state given information about the other. Therefore, a universal data harmoniser will not be able to take advantage of linear correlations in all situations, and will have to be designed to be agnostic in this context.

To address the equally challenging problem of cardinality, the universal harmoniser needs to infer properties directly from the data instead of imposing broad conditions or constraints. Intuitively, it may first appear that flooding a method with multiple layers of information may allow easier signal detection. However, increasing the quantity of modalities surveyed may further amplify the non-linear relationship between omics datasets, adding noise to the data. Furthermore, different features may be either sparse or enriched in different omics datasets and combinations of datasets.

***Usability***

In the context of usability, data should have to undergo minimal preprocessing. This is advantageous for two reasons, preserving method input data in a state as close to primary data as possible, for example in the form of raw sequence data allows the user to promptly and easily analyse their data. More importantly, assumptions associated with preprocessing or intermediate data analyses are avoided. For the same reason, the method should require minimal parameters. This both lessens user confusion while allowing signals to rise organically from the data. Furthermore, the method should be reasonably generalisable or at least sufficiently flexible to account for unconventional cases. As it is unlikely that any single method is applicable to every possible combination of highly nuanced biological datasets, a method should be reconfigurable depending on a biological domain of interest to account for such cases. For example, deep learning models should be designed to be easily re-trainable on data as long as it is formatted correctly, with possible minor adjustments to model architecture.

To further maximise efficiency, the model should be as computationally efficient as possible. Many workflows can consume significant quantities of data storage, memory and compute time [96, 97, 98], to the extent where handling these issues can require a greater resource investment than the actual experiment [99]. In these scenarios, high performance computing clusters may be required to implement methods, which may not be easily accessible to all users.

For easy installation and reproducibility in line with FAIR data principles [100], the software should contain only required software libraries. A lower degree of portability forces a user to unnecessarily invest resources into managing multiple versions of potentially clashing dependencies and subdependencies of software. This is prevalent even among high quality and widely used programs, for example in the R [101] and Bioconductor [102, 103] ecosystem of biological software. Although virtual environment management libraries exist to address this problem [104], they may not be readily accessible or known to new users. Including this software in well-maintained biological software channels with mature dependency management systems like conda [105] or providing them as a virtual machine environment such as Docker [106] or singularity [107] removes a large barrier to user adoption of software.

***Interpretability***

Results of an ideal universal harmoniser should also be easily interpretable. For instance, it is more intuitive for a biologist to understand a method which highlights a gene pathway of interest, instead of a matrix of values which may require additional processing. Where practical, visualisations should be provided to assist interpretation and supplement other results, and presented in easily accessible and portable formats such as a html report or pdf file [108]. However, objective metrics are equally important to judge the performance of an algorithm on a dataset, such as a true and false positive rate. This protects the user from jumping to misleading conclusions.

***Robustness***

All methods are vulnerable to technical and biological noise, and a universal harmoniser should be robust against these. From a biological perspective, missing values may occur in omics datasets, which results in data that is difficult to com-

pare directly without imputation or other rescue steps.

Further complicating this are technical factors such as class imbalance and sample sizes. An imbalance in sample categories may limit the effectiveness of biological datasets. We take the case of a recent SARS-Cov-2 patient study as an example [109]. In this, two imbalanced sample classes were contrasted with a threefold difference in sample representation across classes. While this was likely unavoidable due to the disruptive effects of the ongoing COVID-19 (Coronavirus Disease 2019) pandemic, this may skew a fragile algorithm towards features in the overrepresented category. This is especially true in cases where two conditions may be closely related or may have a similar signal fingerprint to begin with, such as SARS (Severe Acute Respiratory Syndrome) and COVID-19 [110, 111]. Conclusions drawn from such studies can potentially have global impacts on diagnostic tests and health policies, with downstream effects on public health. Meanwhile, an insufficient sample size may reduce an algorithm's effectiveness or lead to false positive signals from its limited feature set. Finally, technical variation in the data has the potential to significantly contaminate results with non-biological noise, and should be avoided where possible [77]. These three technical problems can be avoided with careful experimental design, but in some cases this may not be possible, especially in situations where sample mass is limited or special biological conditions are under study (eg rare phenotypes, geographical, social, economical and political barriers). Intra-modality harmonisation methods can buffer some of this irrelevant variation [Table 3], but have their limitations.

### *Applications*

There is a wide range of potential applications for a sufficiently advanced universal harmoniser across all fields of biology. On a general level, identifying biological pathways contributing to a phenotype allows a user to establish a molecular fingerprint for an organism's phenotype or cell state. Given one piece of information, the user can then infer the state of the other. In medicine, applying this technique to a patient will improve the speed and accuracy of clinical diagnoses.

With this knowledge, an opportunity to achieve an intended phenotype by targeting the appropriate biomolecular switches exists. Epigenomic editing is still in its infancy, but an active area of research with profound clinical implications across all diseases [112, 113]. CRISPR-Cas9 [114] or small RNA-mediated methods have potential in treating complex diseases such as cystic fibrosis by suppressing the mucin production machinery or by inducing the production of functional CFTR variants [115]. A similar approach reversed an intellectual disability phenotype in mice [116]. Cancer research and treatment is likely to benefit from this as well, as it involves the dysregulation of many pathways and is challenging to treat with conventional therapies [113]. From the opposite perspective, it will be possible to also identify drug side effects by examining the biological pathways they will affect. Clinicians can then design mitigating strategies.

Overall, knowing the biological pathways involved in a complex phenotype at the very least highlights them for further investigation. A deeper understanding of biology will result, which feeds back positively into all possible applications.

## Conclusion

To minimise confusion around method scope, we developed a classification system for data integrative strategies. We define "data harmonisation" as the unification of low-level features across different data modalities, and distinguish this from the broader, inconsistent usage of data integration in the literature.

Although significant barriers to universal multimodal data harmonisation exist, we highlight several points and strategies of interest, which some existing harmonisation methods already account for and implement. To resolve the heterogeneity across different epigenomics datasets, transcriptomics data is used in all state of the art methods as a reference point, as its properties are relatively well understood and it is commonly used in experiments. A hypothetical universal harmoniser can take advantage of this property by making the reasonable assumption that transcriptomics data will be present and using this to anchor a method. This can further act as a bridge between epigenomic and functional omics data, allowing protein and metabolite information to be included. Combining these epigenomic and functional signatures allow a system-level view to be obtained.

A fundamental problem in data harmonisation is to consistently represent heterogeneous epigenomics datasets. A possible solution is reformulating data as generic matrices matched on samples, allowing the use of flexible techniques such as matrix factorisation. In addition, the rising field of deep learning is capable of resolving non-linear relationships in large complex datasets and is therefore well suited to this task. Significant advancements in multimodal data harmonisation are expected by applying a combination of these strategies, and applying this to unlock the full power of both existing and future biological datasets will remove a major bottleneck of systems biology, unlocking a new paradigm of medical applications.

## Availability of source code and requirements

Not applicable

## Availability of supporting data and materials

Not applicable

## Declarations

Not applicable

### List of abbreviations

- ATAC-Seq – assay for transposase-accessible chromatin
- Bulk-cell – describes experiments which target a population of cells at once
- Cardinality – number of elements in a set, in this case surveyed modalities or omics data
- ChIP-Seq – chromatin immunoprecipitation sequencing, assays for protein-DNA interactions
- Chromatin – histone-DNA complexes that package DNA and regulate gene expression
- ChIRP-Seq – Chromatin isolation by RNA purification sequencing, assays for DNA-RNA binding events
- CLiP-Seq – cross-linking immunoprecipitation sequencing, assays for protein-RNA binding events, also see RIP-Seq
- COVID-19 – coronavirus disease 2019, a viral respiratory disease
- CTFR – cystic fibrosis transmembrane conductance regulator, mutations in this transport protein can cause cystic fibrosis
- CRISPR-Cas9 – Clustered Regularly Interspaced Short Palindromic Repeats and CRISPR protein 9, an enzyme system which enables selective gene editing
- DDBJ – DNA Databank of Japan, INSDC collaborator

- DNase-Seq – DNase I hypersensitive sites sequencing, an assay which probes non-chromatin bound DNA
- EBI – European Bioinformatics Institute, INSDC collaborator
- Epigenome – overall state of all gene regulatory switches in the genome
- Epigenetic – gene regulatory mechanisms in the genome
- FAIRE-Seq – Formaldehyde assisted isolation of regulatory elements, an assay which identifies chromatin accessibility
- Harmonising – studying the association between low-level features in data
- Hi-C – high throughput chromosome conformation capture
- IHEC – International Human Epigenomic Consortium
- INSDC – International Nucleotide Sequence Database Collaboration
- Integrating – using multiple data modalities in an analysis, usually at a high level.
- lncRNA – long non coding RNA
- Methyl-Seq – methylation sequencing, an assay which determines the location of methylated bases
- MNase-Seq – Micrococcal nuclease sequencing, an assay which probes histone-free genomic DNA
- Modality – a category of data which is not directly comparable to another category, for example audio and text, used interchangeably with omics
- miRNA – microRNA, a short (23nt length) sequence which selectively represses transcript levels
- noise – variation which masks biological signals in data
- Omics – a category of data which is not directly comparable to another category, for example 3D chromosome structure and metabolic pathways, used interchangeably with modality
- RIP-Seq – RNA immunoprecipitation sequencing, an assay which identifies RNA-protein interactions, also see CLiP-Seq
- RNA-Seq – RNA sequencing, an assay which measures transcript abundance
- SARS - Severe Acute Respiratory Syndrome, a viral respiratory disease
- Single-cell – describes experiments which target a group of single cells
- SRA – Sequence Read Archive, INSDC collaborator
- Steric hindrance – physical blockage at a molecular level which prevents binding
- TAD – topologically associating domain
- TCGA – The Cancer Genome Atlas
- TF – transcription factor, a protein which triggers a chain of events leading to transcription
- TFBS – transcription factor binding site, sites on genomic DNA which can be bound by TF

## Ethical Approval

Not applicable

## Consent for publication

Not applicable

## Competing Interests

The authors declare that they have no competing interests.

## Funding

S. T acknowledges funding from the Faculty Initiative Fund and Australian Women Research Success Grant at Monash University. T. C received funding from the Australian Government Research Training Program Scholarship and Monash Faculty of Science Dean's Postgraduate Research Scholarship.

## Author's Contributions

Conceptualisation, S. T; Formal analysis, S. T, T. C; Funding Acquisition, S. T; Investigation, S. T, T. C; Resources, S. T; Supervision, S. T; Validation, S. T, T. C; Visualisation, S. T, T. C; Writing – original draft, T. C; Writing – review & editing, S. T, T. C.

## Acknowledgements

We thank Dianne Cook and Elizabeth Mason for helpful feedback. We acknowledge and pay respects to the Elders and Traditional Owners of the land on which our four Australian campuses stand.

## Authors' information

S. T is head of the computational biology research group and a research affiliate with the eResearch Centre at Monash University, Australia with over 15 years of experience in bioinformatics. T. C is a PhD candidate in computational biology with the computational biology research group at Monash University, Australia with over 5 years of experience in computational biology. All authors have an interest in developing tools to uncover knowledge from biological data.

## References

1. Nordström KJV, Schmidt F, Gasparoni N, Salhab A, Gasparoni G, Kattler K, et al. Unique and assay specific features of NOMe-, ATAC- and DNase I-seq data. Nucleic acids research 2019;47(20):10580–10596.
2. Stricker SH, Köferle A, Beck S. From profiles to function in epigenomics. Nature Reviews Genetics 2016;18(1):51–66.
3. Buenrostro JD, Giresi PG, Zaba LC, Chang HY, Greenleaf WJ. Transposition of native chromatin for fast and sensitive epigenomic profiling of open chromatin, DNA-binding proteins and nucleosome position. Nature Methods 2013;10(12):1213–1218.
4. Johnson DS, Mortazavi A, Myers RM, Wold B. Genome-wide mapping of in vivo protein-DNA interactions. Science 2007;316(5830):1497–1502.
5. Lieberman-Aiden E, Berkum NLV, Williams L, Imakaev M, Ragoczy T, Telling A, et al. Comprehensive Mapping of Long-Range Interactions Reveals Folding Principles of the Human Genome. Science 2009;33292(October):289–294.
6. Frommer M, McDonald LE, Millar DS, Collis CM, Watt F, Grigg GW, et al. A genomic sequencing protocol that yields a positive display of 5-methylcytosine residues in individual DNA strands. Proceedings of the National Academy of Sciences of the United States of America 1992;89(5):1827–1831.
7. Meissner A, Gnirke A, Bell GW, Ramsahoye B, Lander ES, Jaenisch R. Reduced representation bisulfite sequencing for comparative high-resolution DNA methylation analysis. Nucleic Acids Research 2005;33(18):5868–5877.
8. Meissner A, Mikkelsen TS, Gu H, Wernig M, Hanna J,

Sivachenko A, et al. Genome-scale DNA methylation maps of pluripotent and differentiated cells. Nature 2008;454(7205):766–770.
9. Chu C, Qu K, Zhong F, Artandi S, Chang H. Genomic Maps of Long Noncoding RNA Occupancy Reveal Principles of RNA-Chromatin Interactions. Molecular Cell 2011;44(4):667–678. http://dx.doi.org/10.1016/j.molcel.2011.08.027.
10. Alipanahi B, Delong A, Weirauch MT, Frey BJ. Predicting the sequence specificities of DNA- and RNA-binding proteins by deep learning. Nature Biotechnology 2015;33(8):831–838. http://dx.doi.org/10.1038/nbt.3300.
11. Kim SG, Harwani M, Grama A, Chaterji S. EP-DNN: A Deep Neural Network-Based Global Enhancer Prediction Algorithm. Scientific Reports 2016;6(August):1–13. http://dx.doi.org/10.1038/srep38433.
12. Avsec Ž, Weilert M, Shrikumar A, Alexandari A, Krueger S, Dalal K, et al. Deep learning at base-resolution reveals motif syntax of the cis-regulatory code; 2019. https://www.biorxiv.org/content/10.1101/737981v1.abstract.
13. Hu R, Pei G, Jia P, Zhao Z. Decoding regulatory structures and features from epigenomics profiles: A Roadmap-ENCODE Variational Auto-Encoder (RE-VAE) model. Methods 2019;(October):0–1. https://doi.org/10.1016/j.ymeth.2019.10.012.
14. Jaroszewicz A, Ernst J. An Integrative Approach for Fine-Mapping Chromatin Interactions. Bioinformatics 2019;(November):1–8.
15. Hussein SMI, Puri MC, Tonge PD, Benevento M, Corso AJ, Clancy JL, et al. Genome-wide characterization of the routes to pluripotency. Nature 2014;516(7530):198–206.
16. Moor AE, Golan M, Massasa EE, Lemze D, Weizman T, Shenhav R, et al. Global mRNA polarization regulates translation efficiency in the intestinal epithelium. Science 2017;357(6357):1299–1303.
17. Shah S, Takei Y, Zhou W, Lubeck E, Yun J, Eng CHL, et al. Dynamics and Spatial Genomics of the Nascent Transcriptome by Intron seqFISH. Cell 2018;174(2):363–376.e16. https://doi.org/10.1016/j.cell.2018.05.035.
18. Wan Y, Wei Z, Looger LL, Koyama M, Druckmann S, Keller PJ. Single-Cell Reconstruction of Emerging Population Activity in an Entire Developing Circuit. Cell 2019;179(2):355–372.e23. https://doi.org/10.1016/j.cell.2019.08.039.
19. Schier AF. Single-cell biology: beyond the sum of its parts. Nature Methods 2020;17(January):17–20.
20. Singh A, Shannon CP, Gautier B, Rohart F, Vacher M, Tebbutt SJ, et al. DIABLO: an integrative approach for identifying key molecular drivers from multi-omics assays. Bioinformatics (Oxford, England) 2019;35(17):3055–3062.
21. Chen L, Xu J, Li SC. DeepMF: Deciphering the Latent Patterns in Omics Profiles with a Deep Learning Method. BMC Bioinformatics 2019;20(Suppl 23):1–13. http://dx.doi.org/10.1101/744706.
22. Vogelstein B, Kinzler KW. Cancer genes and the pathways they control. Nature Medicine 2004;10(8):789–799.
23. Flavahan WA, Drier Y, Johnstone SE, Hemming ML, Tarjan DR, Hegazi E, et al. Altered chromosomal topology drives oncogenic programs in SDH-deficient GISTs. Nature 2019;575(7781):229–233. http://dx.doi.org/10.1038/s41586-019-1668-3.
24. Zhao J, Ohsumi TK, Kung JT, Ogawa Y, Grau DJ, Sarma K, et al. Genome-wide Identification of Polycomb-Associated RNAs by RIP-seq. Molecular Cell 2010;40(6):939–953. http://dx.doi.org/10.1016/j.molcel.2010.12.011.
25. Brower-Toland B, Wacker DA, Fulbright RM, Lis JT, Kraus WL, Wang MD. Specific contributions of histone tails and their acetylation to the mechanical stability of nucleosomes. Journal of Molecular Biology 2005;346(1):135–146.
26. Collings CK, Waddell PJ, Anderson JN. Effects of DNA methylation on nucleosome stability. Nucleic Acids Research 2013;41(5):2918–2931.
27. Lorch Y, Maier-Davis B, Kornberg RD. Histone Acetylation Inhibits RSC and Stabilizes the +1 Nucleosome. Molecular Cell 2018;72(3):594–600.e2. https://doi.org/10.1016/j.molcel.2018.09.030.
28. Qian Z, Zhurkin VB, Adhya S. DNA–RNA interactions are critical for chromosome condensation in Escherichia coli. Proceedings of the National Academy of Sciences of the United States of America 2017;114(46):12225–12230.
29. Gil N, Ulitsky I. Regulation of gene expression by cis-acting long non-coding RNAs. Nature Reviews Genetics 2020;21(2):102–117. http://dx.doi.org/10.1038/s41576-019-0184-5.
30. Stein CS, Jadiya P, Zhang X, McLendon JM, Abouassaly GM, Witmer NH, et al. Mitoregulin: A lncRNA-Encoded Microprotein that Supports Mitochondrial Supercomplexes and Respiratory Efficiency. Cell Reports 2018;23(13):3710–3720.e8. https://doi.org/10.1016/j.celrep.2018.06.002.
31. Mongelli A, Martelli F, Farsetti A, Gaetano C. The dark that matters: Long noncoding RNAs as master regulators of cellular metabolism in noncommunicable diseases. Frontiers in Physiology 2019;10(MAY):1–13.
32. Ashwal-Fluss R, Meyer M, Pamudurti NR, Ivanov A, Bartok O, Hanan M, et al. CircRNA Biogenesis competes with Pre-mRNA splicing. Molecular Cell 2014;56(1):55–66. http://dx.doi.org/10.1016/j.molcel.2014.08.019.
33. Fire A, Xu S, Montgomery MK, Kostas SA, Driver SE, Mello CC. Potent and specific genetic interference by double-stranded RNA in Caenorhabditis elegans. Nature 1998;391(February):806–811.
34. Waterhouse PM, Graham MW, Wang MB. Virus resistance and gene silencing in plants can be induced by simultaneous expression of sense and antisense RNA. Proceedings of the National Academy of Sciences of the United States of America 1998;95(23):13959–13964.
35. Zhang X, Zhou Y, Chen S, Li W, Chen W, Gu W. LncRNA MACC1-AS1 sponges multiple miRNAs and RNA-binding protein PTBP1. Oncogenesis 2019;8(12). http://dx.doi.org/10.1038/s41389-019-0182-7.
36. Hou XX, Cheng H. Long non-coding RNA RMST silencing protects against middle cerebral artery occlusion (MCAO)-induced ischemic stroke. Biochemical and Biophysical Research Communications 2018;495(4):2602–2608. https://doi.org/10.1016/j.bbrc.2017.12.087.
37. Schmitt AM, Garcia JT, Hung T, Flynn RA, Shen Y, Qu K, et al. An inducible long noncoding RNA amplifies DNA damage signaling. Nature Genetics 2016;48(11):1370–1376.
38. Jacob F, Monod J. Genetic regulatory mechanisms in the synthesis of proteins. Journal of Molecular Biology 1961;3(3):318–356.
39. Hansen TB, Jensen TI, Clausen BH, Bramsen JB, Finsen B, Damgaard CK, et al. Natural RNA circles function as efficient microRNA sponges. Nature 2013;495(7441):384–388. http://dx.doi.org/10.1038/nature11993.
40. Barrett SP, Salzman J. Circular RNAs: Analysis, expression and potential functions. Development (Cambridge) 2016;143(11):1838–1847.
41. Ballouz S, Dobin A, Gillis JA. Is it time to change the reference genome? Genome Biology 2019;20(1):1–9.
42. Cho YS, Kim H, Kim HM, Jho S, Jun J, Lee YJ, et al. An ethnically relevant consensus Korean reference genome is a step towards personal reference genomes. Nature Communications 2016;7.

43. Alkan C, Sajjadian S, Eichler EE. Limitations of next-generation genome sequence assembly. Nature Methods 2011;8(1):61–65.
44. Talukder A, Saadat S, Li X, Hu H, Berger B. EPIP: A novel approach for condition-specific enhancer-promoter interaction prediction. Bioinformatics 2019;35(20):3877–3883.
45. Schmidt F, Kern F, Schulz MH. Integrative prediction of gene expression with chromatin accessibility and conformation data. Epigenetics and Chromatin 2020;13(4):1–17. http://biorxiv.org/content/early/2019/07/16/704478.abstract.
46. Bonnet E, Calzone L, Michoel T. Integrative Multi-omics Module Network Inference with Lemon-Tree. PLoS Computational Biology 2015;11(2):1–16.
47. Silva TC, Coetzee SG, Gull N, Yao L, Hazelett DJ, Noushmehr H, et al. ELmer v.2: An r/bioconductor package to reconstruct gene regulatory networks from DNA methylation and transcriptome profiles. Bioinformatics 2019;35(11):1974–1977.
48. Vaske CJ, Benz SC, Sanborn JZ, Earl D, Szeto C, Zhu J, et al. Inference of patient-specific pathway activities from multi-dimensional cancer genomics data using PARADIGM. Bioinformatics 2010;26(12):237–245.
49. Kirk P, Griffin JE, Savage RS, Ghahramani Z, Wild DL. Bayesian correlated clustering to integrate multiple datasets. Bioinformatics 2012;28(24):3290–3297.
50. Zhu J, Sova P, Xu Q, Dombek KM, Xu EY, Vu H, et al. Stitching together multiple data dimensions reveals interacting metabolomic and transcriptomic networks that modulate cell regulation. PLoS Biology 2012;10(4).
51. Gao T, Qian J. Eagle: An algorithm that utilizes a small number of genomic features to predict tissue/ cell type-specific enhancer-gene interactions. PLoS Computational Biology 2019;15(10):1–22.
52. Corradin O, Saiakhova A, Akhtar-Zaidi B, Myeroff L, Willis J, Cowper-Sallari R, et al. Combinatorial effects of multiple enhancer variants in linkage disequilibrium dictate levels of gene expression to confer susceptibility to common traits. Genome Research 2014;24(1):1–13.
53. Koh HWL, Fermin D, Vogel C, Choi KP, Ewing RM, Choi H. iOmicsPASS: network-based integration of multiomics data for predictive subnetwork discovery. npj Systems Biology and Applications 2019;5(1). http://dx.doi.org/10.1038/s41540-019-0099-y.
54. Glass K, Huttenhower C, Quackenbush J, Yuan GC. Passing Messages between Biological Networks to Refine Predicted Interactions. PLoS ONE 2013;8(5).
55. He B, Chen C, Teng L, Tan K. Global view of enhancer-promoter interactome in human cells. Proceedings of the National Academy of Sciences of the United States of America 2014;111(21).
56. Cao Q, Anyansi C, Hu X, Xu L, Xiong L, Tang W, et al. Reconstruction of enhancer-target networks in 935 samples of human primary cells, tissues and cell lines. Nature Genetics 2017;49(10):1428–1436.
57. Roy S, Siahpirani AF, Chasman D, Knaack S, Ay F, Stewart R, et al. A predictive modeling approach for cell line-specific long-range regulatory interactions. Nucleic Acids Research 2015;43(18):8694–8712.
58. Aran D, Sabato S, Hellman A. DNA methylation of distal regulatory sites characterizes dysregulation of cancer genes. Genome Biology 2013;14(3).
59. Rhie SK, Guo Y, Tak YG, Yao L, Shen H, Coetzee GA, et al. Identification of activated enhancers and linked transcription factors in breast, prostate, and kidney tumors by tracing enhancer networks using epigenetic traits. Epigenetics and Chromatin 2016;9(1):1–17.
60. Dhingra P, Martinez-Fundichely A, Berger A, Huang FW, Forbes AN, Liu EM, et al. Identification of novel prostate cancer drivers using RegNetDriver: A framework for integration of genetic and epigenetic alterations with tissue-specific regulatory network. Genome Biology 2017;18(1):1–23.
61. Ho TK. Random decision forests. Proceedings of the International Conference on Document Analysis and Recognition, ICDAR 1995;1:278–282.
62. Shen R, Olshen AB, Ladanyi M. Integrative clustering of multiple genomic data types using a joint latent variable model with application to breast and lung cancer subtype analysis. Bioinformatics 2009;25(22):2906–2912.
63. Leppäaho E, Ammad-Ud-Din M, Kaski S. GFA: Exploratory analysis of multiple data sources with group factor analysis. Journal of Machine Learning Research 2017;18:1–5.
64. Argelaguet R, Arnol D, Bredikhin D, Deloro Y, Velten B, Marioni JC, et al. MOFA+: a probabilistic framework for comprehensive integration of structured single-cell data. bioRxiv 2019;p. 837104. https://www.biorxiv.org/content/10.1101/837104v1.
65. Zhang S, Liu CC, Li W, Shen H, Laird PW, Zhou XJ. Discovery of multi-dimensional modules by integrative analysis of cancer genomic data. Nucleic Acids Research 2012;40(19):9379–9391.
66. Yang Z, Michailidis G. A non-negative matrix factorization method for detecting modules in heterogeneous omics multi-modal data. Bioinformatics 2016;32(1):1–8.
67. Welch JD, Kozareva V, Ferreira A, Vanderburg C, Martin C, Macosko EZ. Single-Cell Multi-omic Integration Compares and Contrasts Features of Brain Cell Identity. Cell 2019;177(7):1873–1887.e17. https://doi.org/10.1016/j.cell.2019.05.006.
68. Ching T, Himmelstein DS, Beaulieu-Jones BK, Kalinin AA, Do BT, Way GP, et al. Opportunities and obstacles for deep learning in biology and medicine. Journal of the Royal Society Interface 2018;15(141).
69. Stuart T, Butler A, Hoffman P, Hafemeister C, Papalexi E, Mauck WM, et al. Comprehensive Integration of Single-Cell Data. Cell 2019;177(7):1888–1902.e21. https://linkinghub.elsevier.com/retrieve/pii/S0092867419305598.
70. Chen L, Xu J, Li SC. DeepMF: Deciphering the Latent Patterns in Omics Profiles with a Deep Learning Method. BMC Bioinformatics 2019;20(23). http://dx.doi.org/10.1101/744706.
71. Lock EF, Hoadley KA, Marron JS, Nobel AB. Joint and individual variation explained (JIVE) for integrated analysis of multiple data types. Annals of Applied Statistics 2013;7(1):523–542.
72. Tenenhaus A, Philippe C, Guillemot V, Le Cao KA, Grill J, Frouin V. Variable selection for generalized canonical correlation analysis. Biostatistics 2014;15(3):569–583.
73. Dimitrakopoulos C, Hindupur SK, Hafliger L, Behr J, Montazeri H, Hall MN, et al. Network-based integration of multi-omics data for prioritizing cancer genes. Bioinformatics 2018;34(14):2441–2448.
74. Argelaguet R, Velten B, Arnol D, Dietrich S, Zenz T, Marioni JC, et al. Multi-Omics Factor Analysis—a framework for unsupervised integration of multi-omics data sets. Molecular Systems Biology 2018;14(6):1–13.
75. Wang B, Mezlini AM, Demir F, Fiume M, Tu Z, Brudno M, et al. Similarity network fusion for aggregating data types on a genomic scale. Nature Methods 2014;11(3):333–337.
76. Li W, Zhang S, Liu CC, Zhou XJ. Identifying multi-layer gene regulatory modules from multi-dimensional genomic data. Bioinformatics 2012;28(19):2458–2466.

77. Leek JT, Scharpf RB, Bravo HC, Simcha D, Langmead B, Johnson WE, et al. Tackling the widespread and critical impact of batch effects in high-throughput data. Nature Reviews Genetics 2010;11(10):733–739.
78. Haghverdi L, Lun ATL, Morgan MD, Marioni JC. Batch effects in single-cell RNA-sequencing data are corrected by matching mutual nearest neighbors. Nature Biotechnology 2018;36(5):421–427.
79. Risso D, Ngai J, Speed TP, Dudoit S. Normalization of RNA-seq data using factor analysis of control genes or samples. Nature Biotechnology 2014;32(9):896–902.
80. Ritchie ME, Phipson B, Wu D, Hu Y, Law CW, Shi W, et al. Limma powers differential expression analyses for RNA-sequencing and microarray studies. Nucleic Acids Research 2015;43(7):e47.
81. Mecham BH, Nelson PS, Storey JD. Supervised normalization of microarrays. Bioinformatics 2010;26(10):1308–1315.
82. Rohart F, Eslami A, Matigian N, Bougeard S, Lê Cao KA. MINT: A multivariate integrative method to identify reproducible molecular signatures across independent experiments and platforms. BMC Bioinformatics 2017;18(1):1–13.
83. Hie B, Bryson B, Berger B. Efficient integration of heterogeneous single-cell transcriptomes using Scanorama. Nature Biotechnology 2019;37(6):685–691. http://dx.doi.org/10.1038/s41587-019-0113-3.
84. Wang M, Fischer J, Song YS. Three-way clustering of multi-tissue multi-individual gene expression data using semi-nonnegative tensor decomposition. bioRxiv 2017;.
85. Altenbuchinger M, Schwarzfischer P, Rehberg T, Reinders J, Kohler CW, Gronwald W, et al. Molecular signatures that can be transferred across different omics platforms. Bioinformatics 2017 07;33(14):i333–i340. https://doi.org/10.1093/bioinformatics/btx241.
86. Stunnenberg HG, Abrignani S, Adams D, de Almeida M, Altucci L, Amin V, et al. The International Human Epigenome Consortium: A Blueprint for Scientific Collaboration and Discovery. Cell 2016;167(5):1145–1149.
87. Tomczak K, Czerwińska P, Wiznerowicz M. The Cancer Genome Atlas (TCGA): An immeasurable source of knowledge. Wspolczesna Onkologia 2015;1A:A68–A77.
88. Davis CA, Hitz BC, Sloan CA, Chan ET, Davidson JM, Gabdank I, et al. The Encyclopedia of DNA elements (ENCODE): Data portal update. Nucleic Acids Research 2018;46(D1):D794–D801.
89. Bernstein BE, Stamatoyannopoulos JA, Costello JF, Ren B, Milosavljevic A, Meissner A, et al. The NIH roadmap epigenomics mapping consortium. Nature Biotechnology 2010;28(10):1045–1048. http://dx.doi.org/10.1038/nbt1010-1045.
90. Athar A, Füllgrabe A, George N, Iqbal H, Huerta L, Ali A, et al. ArrayExpress update - From bulk to single-cell expression data. Nucleic Acids Research 2019;47(D1):D711–D715.
91. Barrett T, Wilhite SE, Ledoux P, Evangelista C, Kim IF, Tomashevsky M, et al. NCBI GEO: Archive for functional genomics data sets - Update. Nucleic Acids Research 2013;41(D1):991–995.
92. Leinonen R, Sugawara H, Shumway M. The sequence read archive. Nucleic Acids Research 2011;39(SUPPL. 1):2010–2012.
93. Mashima J, Kodama Y, Fujisawa T, Katayama T, Okuda Y, Kaminuma E, et al. DNA Data Bank of Japan. Nucleic Acids Research 2017;45(D1):D25–D31.
94. Cook CE, Bergman MT, Cochrane G, Apweiler R, Birney E. The European Bioinformatics Institute in 2017: Data coordination and integration. Nucleic Acids Research 2018;46(D1):D21–D29.
95. Choi J, Pacheco CM, Mosbergen R, Korn O, Chen T, Nagpal I, et al. Stemformatics: Visualize and download curated stem cell data. Nucleic Acids Research 2019;47(D1):D841–D846.
96. Li H, Durbin R. Fast and accurate short read alignment with Burrows-Wheeler transform. Bioinformatics 2009;25(14):1754–1760.
97. Di Tommaso P, Moretti S, Xenarios I, Orobitg M, Montanyola A, Chang JM, et al. T-Coffee: A web server for the multiple sequence alignment of protein and RNA sequences using structural information and homology extension. Nucleic Acids Research 2011;39(SUPPL. 2):13–17.
98. Bankevich A, Nurk S, Antipov D, Gurevich AA, Dvorkin M, Kulikov AS, et al. SPAdes: A new genome assembly algorithm and its applications to single-cell sequencing. Journal of Computational Biology 2012;19(5):455–477.
99. Papageorgiou L, Eleni P, Raftopoulou S, Mantaiou M, Vlachakis D, Hospital CD, et al. Genomic big data hitting the storage bottleneck. EMBnetjournal 2018;24(e910).
100. Jimenez RC, Kuzak M, Alhamdoosh M, Barker M, Batut B, Borg M, et al. Four simple recommendations to encourage best practices in research software. F1000Research 2017;6:1–14.
101. R Core Team, R: A language and environment for statistical computing. Vienna, Austria: R Foundation for Statistical Computing; 2018. https://www.r-project.org/.
102. Gentleman RC, Carey VJ, Bates DM, Bolstad B, Dettling M, Dudoit S, et al. Bioconductor: open software development for computational biology and bioinformatics. Genome biology 2004;5(10).
103. Huber W, Carey VJ, Gentleman R, Anders S, Carlson M, Carvalho BS, et al. Orchestrating high-throughput genomic analysis with Bioconductor. Nature Methods 2015;12(2):115–121.
104. Ushey K, McPherson J, Cheng J, Atkins A, Allaire J, packrat: A Dependency Management System for Projects and their R Package Dependencies. The Comprehensive R Archive Network; 2018. https://github.com/rstudio/packrat/.
105. Anaconda Software Distribution, Anaconda. Anaconda Software Distribution; 2016. https://anaconda.com.
106. Merkel D. Docker: lightweight Linux containers for consistent development and deployment. Linux Journal 2014;239(2):1. https://dl.acm.org/doi/10.5555/2600239.2600241.
107. Kurtzer GM, Sochat V, Bauer MW. Singularity: Scientific containers for mobility of compute. PLoS ONE 2017;12(5):1–20.
108. Bailey TL, Williams N, Misleh C, Li WW. MEME: Discovering and analyzing DNA and protein sequence motifs. Nucleic Acids Research 2006;34(WEB. SERV. ISS.):369–373.
109. Xiong Y, Liu Y, Cao L, Wang D, Guo M, Guo D, et al. Transcriptomic characteristics of bronchoalveolar lavage fluid and peripheral blood mononuclear cells in COVID-19 patients. Social Science Research Network Preprint 2020;.
110. Wu F, Zhao S, Yu B, Chen YM, Wang W, Song ZG, et al. A new coronavirus associated with human respiratory disease in China. Nature 2020;579(March).
111. Zhou P, Yang XL, Wang XG, Hu B, Zhang L, Zhang W, et al. A pneumonia outbreak associated with a new coronavirus of probable bat origin. Nature 2020;579(March). http://dx.doi.org/10.1038/s41586-020-2012-7.
112. Mussolino C, Alzubi J, Pennucci V, Turchiano G, Cathomen T. Genome and Epigenome Editing to Treat Disorders of the Hematopoietic System. Human Gene Therapy 2017;28(11):1105–1115.
113. Roberti A, Valdes AF, Torrecillas R, Fraga MF, Fernandez AF. Epigenetics in cancer therapy and nanomedicine. Clin-

ical Epigenetics 2019;11(1):1–18.
114. Jinek M, Chylinski K, Fonfara I, Hauer M, Doudna JA, Charpentier E. A Programmable Dual-RNA–Guided DNA Endonuclease in Adaptive Bacterial Immunity. Science 2012;337(August):816–822.
115. Bardin P, Sonneville F, Corvol H, Tabary O. Emerging microRNA therapeutic approaches for cystic fibrosis. Frontiers in Pharmacology 2018;9(OCT):1–11.
116. Peter CJ, Saito A, Hasegawa Y, Tanaka Y, Nagpal M, Perez G, et al. In vivo epigenetic editing of Sema6a promoter reverses transcallosal dysconnectivity caused by C11orf46/Arl14ep risk gene. Nature Communications 2019;10(1). http://dx.doi.org/10.1038/s41467-019-12013-y.

 Click here to access/download;Personal Cover;Response to Reviewers comments.pdf

Dear Dr Nogoy

We are very thankful to the reviewers for their time and critical comments and supportive feedback on our article. We have addressed all the questions below and our responses are highlighted in blue. We have also made necessary changes in the manuscript and edits are highlighted in yellow.

Reviewer reports:

Reviewer #1: The authors review modern approaches for the integrative analysis of several datasets. I think that the authors provide a good overview of recent methods and the provided tables with classification of supported data types will be useful.

I have a few comments that the authors should address

Comments:

Fig. 1: I would not call it methylation marks. This may be confused with histone methylation, as these are often referred to as histone marks. Instead I would subtitle Methyl-Seq simply with DNA methylation.

Along those lines, it would be nice if the authors could cite a more recent paper about genome-wide methylation sequencing, not only the classical one from 1992, where the basic method was developed but not applied genome-wide.

- [Figure 1] To unambiguously refer to DNA methylation we changed "Methylation marks" to "DNA methylation".
- [line 26] To refer to genome wide methylation we now also cited Meissner et al 2005 on RRBS and the following 2008 Meissner et al application of the method to the whole genome.
- [Figure 1] Corrected a typo where we referred to RIP-seq as capturing DNA:RNA interactions instead of RNA:protein interactions.

Fig. 2: The distinct highlighting of measured regions for FAIRE and DNase-seq compared to ATAC-seq is misleading. In principle all of these assays will capture, nucleosome free regions, promoters and gene body. Of course each of these assays, depending on how the exact experimental setup is, will show enrichment in regions that other assays may miss (see a recent comparison we did for example: Unique and assay specific features of NOMe-, ATAC- and DNase I-seq data, NAR 2019).

- [Fig 2] Correctly aligned readout of FAIRE, DNaseI-seq, ATAC-seq on tracks.
- [Fig 2] In figure legends, we noted that each assay may have better performance depending on the experimental design and region targeted, with a reference.

I would recommend to refer to it as DNaseI-seq, to make clear which Dnase is being used.

- [Fig 2] Changed DNase-Seq to DNaseI-Seq to avoid ambiguity.

Also why do all three assays cover the complete gene body? In fact there is no reason to expect that these would cover anything except nucleosome-free regions in gene bodies, which would be intragenic enhancers most often in introns of genes. Other than that only at the promoter one normally sees strong signals.

- [Fig 2] We originally oversimplified this and have now partitioned gene regions for clarity.

Furthermore, I find the marking for methyl-seq also confusing. What is meant by this? Bisulfite sequencing for example covers the whole genome, but only Cytosine methylation is captured. In their current picture this is not clear.

- [Fig 2] For DNA methylation, we originally intended to indicate that this hypothetical genome had a methylation mark only at this location, which bisulfite sequencing captured. This explains the empty coverage at non-methylated regions. We now clarified this in figure legends, and explained that bisulfite sequencing normally covers the whole genome.

Fig. 3: it would be nice to indicate in the figure what the difference between inter and intra modality means. It may not be obvious to everyone as these terms are not often used, particular not concering the two different categories of inter-modality. They should also be clearly defined in the text.

- [193-204] Defined inter and intra modality in text (Existing data integration approaches).
- [Fig 3] Reworked figure layout and colour coded methods.

Why do they say that 4 approaches are common, when 5 are shown in the last row of Figure 3?

- [Fig 3] Now correctly stated that 5 approaches are shown in legends. We initially grouped latent and multivariate variables into the same superfamily of methods.

I don't like the arrangement of these 5 approaches in the figure. It is not clear if these are universal or belong to one of the types. I would include something like: Common approaches and then give them numbers from 1 to 5. Or maybe split them according to the use in restricted categories or generic categories?

- [Fig 3] To show approach membership, reworked figure layout and colour coded methods

In the figure caption they mention that the 5 approaches are used for inter modality approaches and only mention linear models for intra-modality. I cannot relate to this, in

particular in the context of single cell analysis all of the 5 approaches have been suggested for intra-modality analysis I believe. I think this distinction should be removed.

- [Fig 3] Removed distinction, merged conceptually similar approaches used by functionally different methods by colour coding

Also what does the black circle with ruler and pen mean?

- [Fig 3] Intended to represent custom workflows, replaced with text "Custom workflow".

-When the authors talk about Ensembl clustering (page 4 top, right column), I think they should really say ensembl learning. For example the first reference EPIP that they cite after their statement, does not use Random forest clustering, but another type of ensembl classifiers.

- [222-223] Corrected this statement to align with the definition of ensemble clustering (combine clusters) and ensemble learners (combine models).

Minor:

-Page 3. This sentence: "These include three-dimensional chromosome structure as measured with Hi-C [4], chromatin occu- pancy probed by ATAC-Seq [2] or ChIP-Seq [3], .." is misleading. Obviously ATAC-seq and ChIP-seq do no not measure the 3d-chromatin structure. If anything the 3d-chromatin structure may be inferred from those assays using additional information, but it seems far fetched in the way currently written.

- [120-123] Our original wording was unclear, now reworded to avoid the implication that ATAC-seq and ChIP-seq can be used to infer 3D structure.

I would like to point the authors to the zeroSum approach, which is a nice example of intra-modality correction for measurements on different platforms (Molecular signatures that can be transferred across different omics platforms, https://www.ncbi.nlm.nih.gov/pubmed/28881975).

- [Table 3] Reviewed zeroSum method and added it to the list, fixed a typo in our original table

Table 2: The caption is too small to be read without zooming in the pdf.

- [Table 1-3] Recoded small tables to be more human-readable.

Table 4/5: It would be good to include the citation to the methods in the table. For example the column "Chromatin occupancy and histone marks" could be called: "Epigenetic peak data" to shorten the text.

- [Table 4-5] Added citation to methods, renamed columns to optimise space.

In this context I would like to point out that sometimes a method may also include peaks from DNA methylation analysis, which is not entirely clear from the table as there is another column for DNA methylation. But I assume that the column DNA methylation refers to the fact that a method is able to handle the beta values explicitely?

- [Table 4-5] Clarified DNA methylation context in table legends

Following the above changes we have also simplified wordings in the conclusion section.

We hope that you find the changes satisfactory and we look forward to hearing from you soon.

With regards

Sonika Tyagi and Tyrone Chen
