# Supplementary material for: Integrative computational epigenomics to build data-driven gene regulation hypotheses
Source: Gigascience. 2020 Jun 16;9(6):giaa064. doi: 10.1093/gigascience/giaa064 (PMC7297091; doi:10.1093/gigascience/giaa064)
Supplement: giaa064_Supplemental_File [file giaa064_supplemental_file.pdf]

# Additional data

# Additional tables

| Epigenomic regulatory feature | Directly associated sub-features | Feature information | Assays | Assay information | Hybrid assays |
|---|---|---|---|---|---|
| 3D chromosome structure | Enhancers<br><br>Silencers<br><br>Insulators | Chromosomal looping can bring arrays of regulatory elements from distant parts of the genome into close contact to form TADs (Topologically Associating Domain).<br><br>Enhancers occur when arrays of permissive regulatory elements are grouped.<br><br>Silencers have the opposite effect, where looping disrupts such events from occurring.<br><br>Insulators can act similarly to silencers, but can also repel chromatin formation to maintain a permissive transcriptional environment. | 3C (Chromosome conformation capture) [1]<br><br>4C (Chromosome conformation capture on chip) [2]<br><br>5C (Chromosome conformation capture carbon copy) [3]<br><br>Hi-C (high throughput Chromosome conformation capture) [4] | Detects interactions between non-adjacent genomic regions<br><br>within the same chromosome<br><br>between different chromosomes | ChIP-loop (Chromatin immunoprecipitation loop) [5]: 3D chromosome structure + Chromatin occupancy<br><br>ChIA-PET (Chromatin Interaction Analysis by Paired-End Tag Sequencing) [6]: 3D chromosome structure + Chromatin occupancy<br><br>Hi-ChIRP (High throughput-Chromatin Isolation by RNA Purification sequencing) [7] |
| DNA methylation | DNA methylation | Steric hindrance or<br><br>active protein recruitment as a result of DNA biochemical modifications exerts a strong repressive effect on gene expression. | Methyl-Seq [8]<br><br>RRBS (Reduced Representation Bisulfite Sequencing) [9]<br><br>Methylation arrays | Selective chemical conversion of methylated nucleotides identifies these bases when comparing to a reference | Methyl-HiC: 3D chromosome structure + DNA methylation |
| Chromatin occupancy and histone marks | Chromatin accessibility<br><br>Histone methylation<br><br>Histone acetylation | Regions of DNA bound by chromatin are less readily accessible to the transcription machinery of a cell compared to unbound regions.<br><br>Core components of chromatin are known as histones, and methylation or acetylation of these proteins alter the stability of chromatin. | ATAC-Seq (Assay for Transposase-Accessible Chromatin) [10]<br><br>DNase-Seq (DNase I hypersensitive sites sequencing) [11] | These assays probe chromatin occupancy by filtering open chromatin regions from the data.<br><br>Most methods exploit enzymatic activity to extract this information, while FAIRE-Seq exploits the protein-DNA cross-linking | ChIP-loop (Chromatin immunoprecipitation loop) [5]: 3D chromosome structure + Chromatin occupancy<br><br>ChIA-PET (Chromatin Interaction Analysis by Paired-End Tag Sequencing) [6]: 3D |

| | | | | | |
|---|---|---|---|---|---|
| | | This increases or decreases the transiency of this DNA-histone complex. | FAIRE-Seq (Formaldehyde-Assisted Isolation of Regulatory Elements) [12,13]<br><br>MNase-Seq (Micrococcal Nuclease sequencing) [14,15] | properties of formaldehyde.<br><br>The remaining histone-bound regions of DNA are then identified through high throughput sequencing.<br><br>The effectiveness of each method varies in different regions of DNA. | chromosome structure + Chromatin occupancy<br><br>SNARE-Seq (droplet-based single nucleus chromatin accessibility and mRNA expression) [16]: Chromatin occupancy + transcriptomics (gene expression) |
| DNA-Protein binding | Transcription Factor Binding Sites (TFBS) | Proteins known as transcription factors bind to these special regions of DNA to modulate transcription.<br><br>Sites are known to exist in promoter as well as enhancer regions.<br><br>It is common for multiple transcription factors to work in tandem. | ATAC-Seq (Assay for Transposase-Accessible Chromatin) [10]<br><br>ChIP-Seq (Chromatin Immunoprecipitation sequencing) [17]<br><br>ChIP-chip (Chromatin immunoprecipitation on chip) [18] | Assays used to probe chromatin occupancy are also used to identify non-histone proteins. | |
| DNA-RNA interactions | enhancer bound RNA<br><br>fusion transcripts | DNA-RNA interactions affect gene silencing, chromatin regulation and may even form hybrid transcripts.<br><br>It is hypothesised that they selectively recruit other elements of the epigenetic regulatory machinery [19].<br><br>Other studies suggest that their binding to enhancers is sufficient to induce transcription [20], or even chromatin condensation [21].<br><br>Conversely, some RNA transcribed at enhancers (eRNA) has been associated with phenotype [22]<br><br>Little is known about their mechanism of action in all scenarios. | ChIRP-Seq (Chromatin Isolation by RNA Purification sequencing) [24] | This variant of ChIP-Seq applies the same concept of immunoprecipitation to target RNA bound to DNA, identifying DNA-RNA binding events.<br><br>It is primarily used to survey long non coding RNA. | Hi-ChIRP (High throughput-Chromatin Isolation by RNA Purification sequencing) [7] |

| | | | | | |
|---|---|---|---|---|---|
| RNA-Protein interactions | RNA binding proteins (RBP) | Non-coding RNA is capable of interacting with proteins to perform gene regulatory functions.<br><br>These proteins can mediate gene activity both during and after transcription by regulating transcript levels and localisation [23].<br><br>Additionally, long non-coding RNA is implicated in genome organisation by acting as a scaffold for DNA and proteins | CLiP-Seq (Cross-linking immunoprecipitation sequencing) [17,25,26]<br><br>PAR-CLiP (photoactivatable ribonucleoside-enhanced cross linking and immunoprecipitation) [27]<br><br>RIP-Seq (RNA immunoprecipitation sequencing) [28] | These variants of ChIP-Seq exploit immunoprecipitation to target RNA bound to protein, identifying these RNA sequences binding events. | |
| Protein-Protein interactions | Protein mediator complexes | Proteins and other regulatory elements rarely act in isolation.<br><br>Large regulatory protein complexes are frequently formed, and the resulting ensemble is often the main functional catalyst.<br><br>An example is the DICER complex in catalysing small RNA synthesis [29,30] | ChIP + MS (Chromatin immunoprecipitation with mass spectrometry) | Target proteins are extracted by immunoprecipitation<br><br>These proteins are identified through mass spectrometry | |
| Genomics | Single nucleotide polymorphisms (SNP)<br><br>Copy number variations (CNV)<br><br>chromosomal rearrangements | An organism's genome sequence provides a one-dimensional blueprint of genomic features.<br><br>Changes to sequences are inducible by many environmental factors, and such changes can have positive or negative effects, particularly if a regulatory element or active protein-coding sequence is disrupted. | Whole genome sequencing<br><br>SNP<br><br>Genome wide association studies | Sequencing a genome reveals the genetic code at a single base resolution.<br><br>Subsequent genome assemblies can be then compared to a reference or among each other. | |
| Transcriptomics | Quantitative gene expression<br><br>small non-coding RNA<br><br>long non-coding RNA | Transcript abundance represents a relatively direct readout of gene activity and is a global feature across most organisms.<br><br>At the same time, non-coding RNA levels can be measured. Small non-coding RNA generally have a silencing effect on their targets, while the scope of action for long non-coding RNAs is broader. | coding RNA-Seq<br><br>non-coding RNA-Seq (long and short)<br><br>Microarrays<br><br>CAGE (Cap analysis gene expression) [31] | Sequencing RNA allows us to quantitatively identify transcripts present in a genome.<br><br>This can apply to any form of RNA, which can correspond to coding or non-coding RNA.<br><br>Microarrays are functionally similar but are restricted to probing known | SNARE-Seq (droplet-based single nucleus chromatin accessibility and mRNA expression) [16]: Chromatin occupancy + transcriptomics (gene expression) |

| | | | | | |
|---|---|---|---|---|---|
| | | These are interesting due to their target-specificity to DNA. | | transcripts.<br><br>CAGE is a special assay which identifies alternative transcription start sites. | |
| Proteomics | Quantitative protein and isoform identification | Many proteins have direct or indirect regulatory roles in the cell.<br><br>While histones, transcription factors and other molecules are often interpreted in context of binding to another biological molecule, abundance levels of these proteins can be informative depending on the system under study. | LC-MS (Liquid Chromatography Mass Spectrometry)<br><br>GC-MS (Gas Chromatography Mass Spectrometry) | Proteins in a sample are identified by mass spectrometry.<br><br>While GC-MS workflows are robust and replicable, they are most effective on small molecules. | |
| Metabolomics | Metabolic pathway of interest | Metabolites are indicative of active or inactive metabolic pathways in the cell.<br><br>In most contexts, the relative abundances of metabolites within or across pathways yield information pertaining to the experiment, but abundance levels of these proteins can be informative depending on the system under study. | LC-MS (Liquid Chromatography<br><br>Mass Spectrometry)<br>GC-MS (Gas Chromatography Mass Spectrometry) | Metabolites in a sample are identified by mass spectrometry.<br><br>While GC-MS workflows are robust and replicable, they are most effective on small molecules. | |

Table S1: Epigenomic regulatory features and their corresponding assays. Common human epigenomic regulatory features are listed, along with their function. Assays used to extract information on each feature are also included. Single cell methods are not covered.

| Repository name | Primary experimental scope | DOI (Digital Object Identifier) |
|---|---|---|
| ArrayExpress | Generic | https://doi.org/10.1093/nar/gky964 [32] |
| ENCODE (Encyclopedia of DNA Elements) | Generic | https://doi.org/10.1038/nature11247 [33] |
| GEO (Gene Expression Omnibus) | Generic | https://doi.org/10.1093/nar/gks1193 [34] |
| IHEC (International Human Epigenome Consortium) | Development, Disease | https://doi.org/10.1016/j.cell.2016.11.007 [35] |
| INSDC* (International Nucleotide Sequence Database Collaboration) | Generic | https://doi.org/10.1093/nar/gkq1019;https://doi.org/10.1093/nar/gkw1001;https://doi.org/10.1093/nar/gkx1154 [36,37,38] |
| Roadmap | Development, Disease | https://doi.org/10.1038/nature14248 [39] |
| Stemformatics | Stem cells | https://doi.org/10.1093/nar/gky1064 [40] |
| TCGA (The Cancer Genome Atlas) | Cancer | https://doi.org/10.5114/wo.2014.47136 [41] |

Table S2: Epigenomic data resources and their scope. Note that data is shared among INSDC member organisations, which include SRA (Sequence Read Archive), EBI (European Bioinformatics Institute) and DDBJ (DNA Database of Japan). Single cell data is not included in this comparison.

| **Category** | **Property** |
|---|---|
| Functionality | Agnostic to omics type |
| | Scalable regardless of omics quantity |
| Usability | Minimal preprocessing |
| | Minimal parameters |
| | Resource usage |
| | Reusability |
| | Speed |
| | Portability |
| Interpretability | Intuitive |
| | Objective metrics |
| | Visual |
| Robustness | Class imbalance |
| | Data complexity |
| | Missing data |
| | Sample sizes |
| | Technical variation |
| Applications | Diagnosis |
| | Understanding |
| | Treatment |

Table S3: Properties of an ideal universal dataset harmoniser.

| **Repository name** | **Modalities available** | **3D chromosome structure** | **DNA methylation** | **Chromatin occupancy and histone marks** | **DNA-Protein binding** | **DNA-RNA interactions** | **RNA-Protein interactions** | **Protein-Protein interactions** | **Genomics** | **Transcriptomics** |
|---|---|---|---|---|---|---|---|---|---|---|
| ArrayExpress | 9 | ✓ | ✓ | ✓ | ✓ | ✓ | ✓ | ✓ | ✓ | ✓ |
| ENCODE (Encyclopedia of DNA Elements) | 7 | ✓ | ✓ | ✓ | ✓ | ✗ | ✓ | ✗ | ✓ | ✓ |
| GEO (Gene Expression Omnibus) | 9 | ✓ | ✓ | ✓ | ✓ | ✓ | ✓ | ✓ | ✓ | ✓ |
| IHEC (International Human Epigenome Consortium) | 5 | ✗ | ✓ | ✓ | ✓ | ✗ | ✗ | ✗ | ✓ | ✓ |
| INSDC* (International Nucleotide Sequence Database Collaboration) | 9 | ✓ | ✓ | ✓ | ✓ | ✓ | ✓ | ✓ | ✓ | ✓ |
| Roadmap | 3 | ✗ | ✓ | ✓ | ✗ | ✗ | ✗ | ✗ | ✗ | ✓ |
| Stemformatics | 4 | ✗ | ✓ | ✓ | ✓ | ✗ | ✗ | ✗ | ✗ | ✓ |
| TGCA | 4 | ✗ | ✓ | ✓ | ✗ | ✗ | ✗ | ✗ | ✓ | ✓ |

Table S4A: Data modalities present in epigenomic databases. Databases are displayed on the vertical axis and scores are displayed on the horizontal axis. Lighter colour indicates a greater quantity of modalities and binary yellow (True) or purple (False) were used to indicate presence of a data modality. For simplicity, some modalities have been aggregated, for example transcriptomics data includes both gene expression and small rna data. Note that data is shared among INSDC member organisations [36,37,38], which include SRA (Sequence Read Archive), EBI (European Bioinformatics Institute) and DDBJ (DNA Database of Japan). Single cell data is not included in this. The publication associated with each database is provided in Table S2 for easy reference.

| Repository name | Accessibility | Standardised laboratory protocols | Standardised software pipelines |
|---|---|---|---|
| ArrayExpress | ◐ | ○ | ○ |
| ENCODE (Encyclopedia of DNA Elements) | ● | ● | ● |
| GEO (Gene Expression Omnibus) | ◐ | ○ | ○ |
| IHEC (International Human Epigenome Consortium) | ◐ | ◐ | ◐ |
| INSDC* (International Nucleotide Sequence Database Collaboration) | ◐ | ○ | ○ |
| Roadmap | ● | ● | ● |
| Stemformatics | ◐ | ○ | ● |
| TGCA | ◐ | ○ | ● |

Table S4B: Data accessibility and standardisation in epigenomic databases. A black circle indicates full accessibility or standardisation, a half-filled circle indicates limited accessibility or limited standardisation, and a blank circle indicates no accessibility or standardisation. Note that data is shared among INSDC member organisations [36,37,38], which include SRA (Sequence Read Archive), EBI (European Bioinformatics Institute) and DDBJ (DNA Database of Japan). Single cell data is not included in this. The publication associated with each database is provided in Table S2 for easy reference.

**Additional figures**

Figure S1: Gene expression is the result of a combination of regulatory feature interactions. Clockwise from top: Insulators maintain an accessible chromatin state permitting transcription within a specific region of the genome, Protein-miRNA interactions modulating gene expression, DNA methylation silences a gene, Silencers alter chromosome conformation to prevent assembly of the transcription machinery, Chromatin accessibility for transcription is lowered by histone binding, DNA-lncRNA interactions regulating gene expression, DNA-Protein interactions transcription factor binds to DNA, triggering a signal cascade leading to transcription, Enhancers reconfigure chromosome structure to increase the likelihood of gene expression.

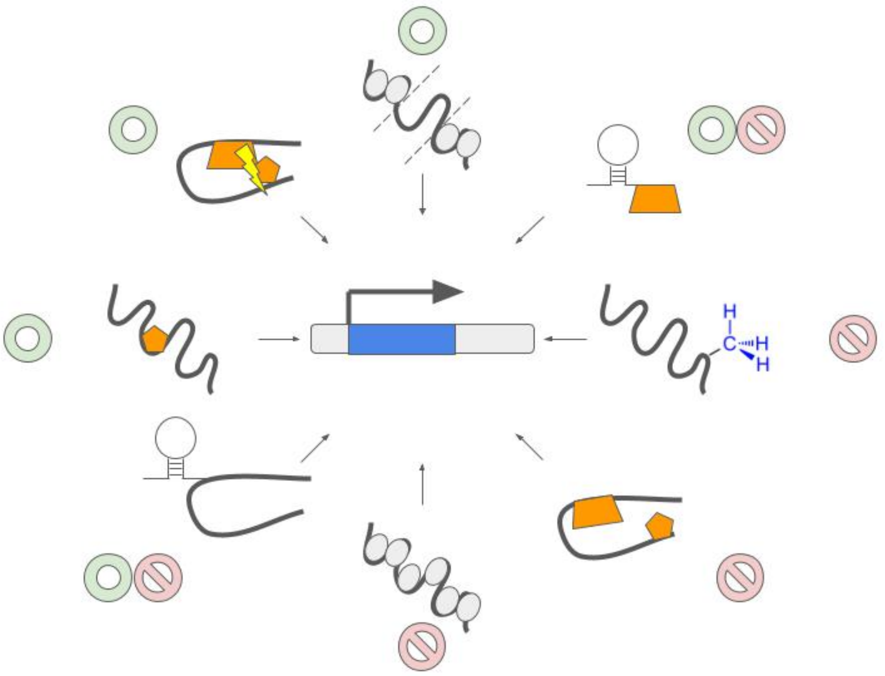

## Extended data references


1. Dekker, J., Rippe, K., Dekker, M. & Kleckner, N. Capturing chromosome conformation. *Science* **295**, 1306–1311 (2002).

2. Simonis, M. *et al.* Nuclear organization of active and inactive chromatin domains uncovered by chromosome conformation capture-on-chip (4C). *Nature Genetics* **38**, 1348–1354 (2006).

3. Dostie, J. *et al.* Chromosome Conformation Capture Carbon Copy (5C): A massively parallel solution for mapping interactions between genomic elements. *Genome Research* **16**, 1299–1309 (2006).

4. Lieberman-Aiden E, Berkum NLV, Williams L, Imakaev M, Ragoczy T, Telling A, et al. Comprehensive Mapping of Long-Range Interactions Reveals Folding Principles of the Human Genome. *Science* **33292**(October):289– 294. (2009)

5. Horike, S. I., Cai, S., Miyano, M., Cheng, J. F. & Kohwi-Shigematsu, T. Loss of silent-chromatin looping and impaired imprinting of DLX5 in Rett syndrome. *Nature Genetics* **37**, 31–40 (2005).

6. Fullwood, M. J. *et al.* An oestrogen-receptor-$\alpha$-bound human chromatin interactome. *Nature* **462**, 58–64 (2009).

7. Mumbach, M. R. *et al.* HiChIRP reveals RNA-associated chromosome conformation. *Nature Methods* **16** (2019). URL `http://www.nature.com/articles/s41592-019-0407-x`.

8. Frommer M, McDonald LE, Millar DS, Collis CM, Watt F, Grigg GW, et al. A genomic sequencing protocol that yields a positive display of 5-methylcytosine residues in individual DNA strands. *Proceedings of the National Academy of Sciences of the United States of America* **89**(5):1827–1831 (1992).

9. Meissner, A. *et al.* Reduced representation bisulfite sequencing for comparative high-resolution DNA methylation analysis. *Nucleic Acids Research* **33**, 5868–5877 (2005).

10. Buenrostro JD, Giresi PG, Zaba LC, Chang HY, Greenleaf WJ. Transposition of native chromatin for fast and sensitive epigenomic proling of open chromatin, DNA binding proteins and nucleosome position. *Nature Methods* **10**(12):1213–1218 (2013).

11. Boyle, A. P. *et al.* High-Resolution Mapping andCharacterization of Open Chromatin across the Genome. *Cell* **132**, 311–322 (2008).

12. Hogan, G. J., Lee, C. K. & Lieb, J. D. Cell cycle-specified fluctuation of nucleosome occupancy at gene promoters. *PLoS Genetics* **2**, 1433–1450(2006).

13. Giresi, P. G. & Lieb, J. D. Isolation of active regulatory elements from eukaryotic chromatin using FAIRE (Formaldehyde Assisted Isolation of

Regulatory Elements). *Methods* **48**, 233–239 (2009). URL http://dx.doi.org/10.1016/j.ymeth.2009.03.003.

14. Solomon, M. J., Larsen, P. L. & Varshavsky, A. Mapping protein-DNA interactions in vivo with formaldehyde: Evidence that histone H4 is retained on a highly transcribed gene. *Cell* **53**, 937–947 (1988).

15. Cusick, M. E., Herman, T. M., DePamphilis, M. L. & Wassarman, P. M. Structure of Chromatin at Deoxyribonucleic Acid Replication Forks: Prenucleosomal Deoxyribonucleic Acid Is Rapidly Excised from Replicating Simian Virus 40 Chromosomes by Micrococcal Nuclease. *Biochemistry* **20**, 6648–6658 (1981).

16. Bemer, M. & Baroux, C. l. B. *Plant Chromatin Dynamics: Methods and Protocols*, vol. 1675 (Humana Press, 2018).

17. Chen, S., Lake, B.B. & Zhang, K. High-throughput sequencing of the transcriptome and chromatin accessibility in the same cell. *Nat Biotechnol* **37,** 1452–1457 (2019). https://doi.org/10.1038/s41587-019-0290-0

18. Solomon, M. J., Larsen, P. L. & Varshavsky, A. Mapping protein-DNA interactions in vivo with formaldehyde: Evidence that histone H4 is retained on a highly transcribed gene. *Cell* **53**, 937–947 (1988).

19. Kuo, C. C. *et al.* Detection of RNA-DNA binding sites in long noncoding RNAs. *Nucleic acids research* **47**, e32 (2019).

20. Yang, L. *et al.* LncRNA-dependent mechanisms of androgen-receptor-regulated gene activation pro- grams. *Nature* **500**, 598–602 (2013). URL http://dx.doi.org/10.1038/nature12451.

21. Qian Z, Zhurkin VB, Adhya S. DNA–RNA interactions are critical for chromosome condensation in Escherichia coli. *Proceedings of the National Academy of Sciences of the United States of America* **114** (46):12225–12230 (2017).

22. Kim, T. K. *et al.* Widespread transcription at neuronal activity-regulated enhancers. *Nature* **465**, 182–187 (2010).

23. Glisovic, T., Bachorik, J. L., Yong, J. & Dreyfuss, G. RNA-binding proteins and post-transcriptional gene regulation. *FEBS Letters* **582**, 1977–1986(2008).

24. Chu, Ci *et al*. Genomic Maps of Long Noncoding RNA Occupancy Reveal Principles of RNA-Chromatin Interactions. *Molecular Cell* **44**, (4):667–678 (2011).

25. Chi, S. W., Zang, J. B., Mele, A. & Darnell, R. B. Argonaute HITS-CLIP decodes microRNA-mRNA interaction maps. *Nature* **460**, 479–486(2009).

26. Xue, Y. *et al.* Genome-wide Analysis of PTB-RNA Interactions Reveals a Strategy Used by the General Splicing Repressor to Modulate Exon Inclusion or

Skipping. *Molecular Cell* **36**, 996–1006 (2009). URL http://dx.doi.org/10.1016/j.molcel.2009.12.003.

27. Hafner, M. *et al.* Transcriptome-wide Identification of RNA-Binding Protein and MicroRNA Target Sites by PAR-CLIP. *Cell* **141**, 129–141 (2010).

28. Zhao, J. *et al.* Genome-wide Identification of Polycomb-Associated RNAs by RIP-seq. *Molecular Cell* **40**, 939–953 (2010). URL http://dx.doi.org/10.1016/j.molcel.2010.12.011.

29. Fire, A. *et al.* Potent and specific genetic interference by double-stranded RNA in Caenorhabditis elegans. *Nature* **391**, 806–811 (1998).

30. Waterhouse, P. M., Graham, M. W. & Wang, M. B. Virus resistance and gene silencing in plants can be induced by simultaneous expression of sense and antisense RNA. *Proceedings of the National Academy of Sciences of the United States of America* **95**, 13959–13964 (1998).

31. Takahashi, H., Lassmann, T., Murata, M. & Carninci, P. 5 End-Centered Expression Profiling Using Cap-Analysis Gene Expression and Next-Generation Sequencing. *Nature Protocols* **7**, 542–561 (2012).

32. Athar A, Füllgrabe A, George N, Iqbal H, Huerta L, Ali A, et al. ArrayExpress update - From bulk to single-cell expression data. *Nucleic Acids Research* **47**(D1):D711– D715 (2019).

33. Davis CA, Hitz BC, Sloan CA, Chan ET, Davidson JM, Gabdank I, et al. The Encyclopedia of DNA elements (ENCODE): Data portal update. *Nucleic Acids Research* **46**(D1):D794–D801 (2018).

34. Barrett T, Wilhite SE, Ledoux P, Evangelista C, Kim IF, Tomashevsky M, et al. NCBI GEO: Archive for functional genomics data sets - Update. *Nucleic Acids Research* **41**(D1):991–995 (2013).

35. Stunnenberg HG, Abrignani S, Adams D, de Almeida M, Altucci L, Amin V, et al. The International Human Epigenome Consortium: A Blueprint for Scientic Collaboration and Discovery. *Cell* **167**(5):1145–1149 (2016).

36. Leinonen R, Sugawara H, Shumway M. The sequence read archive. *Nucleic Acids Research* **39**(SUPPL. 1):2010– 2012. 83 (2011).

37. Mashima J, Kodama Y, Fujisawa T, Katayama T, Okuda Y, Kaminuma E, et al. DNA Data Bank of Japan. *Nucleic Acids Research* **45**(D1):D25–D31. 84 (2017).

38. Cook CE, Bergman MT, Cochrane G, Apweiler R, Birney E. The European Bioinformatics Institute in 2017: Data coordination and integration. *Nucleic Acids Research* **46**(D1):D21–D29 (2018).

39. Bernstein BE, Stamatoyannopoulos JA, Costello JF, Ren B, Milosavljevic A, Meissner A, et al. The NIH roadmap epigenomics mapping consortium. *Nature*

*Biotechnology* **28**(10):1045–1048 (2010). http://dx.doi.org/10.1038/ nbt1010-1045.

40. Choi J, Pacheco CM, Mosbergen R, Korn O, Chen T, Nagpal I, et al. Stemformatics: Visualize and download curated stem cell data. *Nucleic Acids Research* **47**(D1):D841– D846 (2019).

41. Tomczak K, Czerwinska P, Wiznerowicz M. The Cancer Genome Atlas (TCGA): An immeasurable source of knowledge. *Wspolczesna Onkologia* **1A**:A68–A77 (2015).
