# Supplementary material for: Integrative computational epigenomics to build data-driven gene regulation hypotheses
Source: Gigascience. 2020 Jun 16;9(6):giaa064. doi: 10.1093/gigascience/giaa064 (PMC7297091; doi:10.1093/gigascience/giaa064)
Supplement: giaa064_Response_to_Reviewer_Comments_Original_Submission [file giaa064_response_to_reviewer_comments_original_submission.pdf]

Author's Response To Reviewer Comments

Close

Dear Dr Nogoy
We are very thankful to the reviewers for their time and critical comments and supportive feedback on our article. We have addressed all the questions below and our responses are highlighted in blue. We have also made necessary changes in the manuscript and edits are highlighted in yellow.

Reviewer reports:
Reviewer #1: The authors review modern approaches for the integrative analysis of several datasets. I think that the authors provide a good overview of recent methods and the provided tables with classification of supported data types will be useful.
I have a few comments that the authors should address
Comments:
Fig. 1: I would not call it methylation marks. This may be confused with histone methylation, as these are often referred to as histone marks. Instead I would subtitle Methyl-Seq simply with DNA methylation.
Along those lines, it would be nice if the authors could cite a more recent paper about genome-wide methylation sequencing, not only the classical one from 1992, where the basic method was developed but not applied genome-wide.
Response: [Figure 1] To unambiguously refer to DNA methylation we changed "Methylation marks" to "DNA methylation".
Response: [line 26] To refer to genome wide methylation we now also cited Meissner et al 2005 on RRBS and the following 2008 Meissner et al application of the method to the whole genome.
Response: [Figure 1] Corrected a typo where we referred to RIP-seq as capturing DNA:RNA interactions instead of RNA:protein interactions.
Fig. 2: The distinct highlighting of measured regions for FAIRE and DNase-seq compared to ATAC-seq is misleading. In principle all of these assays will capture, nucleosome free regions, promoters and gene body. Of course each of these assays, depending on how the exact experimental setup is, will show enrichment in regions that other assays may miss (see a recent comparison we did for example: Unique and assay specific features of NOMe-, ATAC- and DNase I-seq data, NAR 2019).
Response: [Fig 2] Correctly aligned readout of FAIRE, DNaseI-seq, ATAC-seq on tracks.
Response: [Fig 2] In figure legends, we noted that each assay may have better performance depending on the experimental design and region targeted, with a reference.
I would recommend to refer to it as DNaseI-seq, to make clear which Dnase is being used.
Response: [Fig 2] Changed DNase-Seq to DNaseI-Seq to avoid ambiguity.
Also why do all three assays cover the complete gene body? In fact there is no reason to expect that these would cover anything except nucleosome-free regions in gene bodies, which would be intragenic enhancers most often in introns of genes. Other than that only at the promoter one normally sees strong signals.
Response: [Fig 2] We originally oversimplified this and have now partitioned gene regions for clarity.
Furthermore, I find the marking for methyl-seq also confusing. What is meant by this? Bisulfite sequencing for example covers the whole genome, but only Cytosine methylation is captured. In their current picture this is not clear.
Response: [Fig 2] For DNA methylation, we originally intended to indicate that this hypothetical genome had a methylation mark only at this location, which bisulfite sequencing captured. This explains the empty coverage at non-methylated regions. We now clarified this in figure legends, and explained that bisulfite sequencing normally covers the whole genome.
Fig. 3: it would be nice to indicate in the figure what the difference between inter and intra modality means. It may not be obvious to everyone as these terms are not often used, particular not concering the two different categories of inter-modality. They should also be clearly defined in the text.
Response: [193-204] Defined inter and intra modality in text (Existing data integration approaches).
Response: [Fig 3] Reworked figure layout and colour coded methods.
Why do they say that 4 approaches are common, when 5 are shown in the last row of Figure 3?
Response: [Fig 3] Now correctly stated that 5 approaches are shown in legends. We initially grouped latent and multivariate variables into the same superfamily of methods.

I don't like the arrangement of these 5 approaches in the figure. It is not clear if these are universal or belong to one of the types. I would include something like: Common approaches and then give them numbers from 1 to 5. Or maybe split them according to the use in restricted categories or generic categories?
Response: [Fig 3] To show approach membership, reworked figure layout and colour coded methods
In the figure caption they mention that the 5 approaches are used for inter modality approaches and only mention linear models for intra-modality. I cannot relate to this, in particular in the context of single cell analysis all of the 5 approaches have been suggested for intra-modality analysis I believe. I think this distinction should be removed.
Response: [Fig 3] Removed distinction, merged conceptually similar approaches used by functionally different methods by colour coding
Also what does the black circle with ruler and pen mean?
Response: [Fig 3] Intended to represent custom workflows, replaced with text "Custom workflow".
-When the authors talk about Ensembl clustering (page 4 top, right column), I think they should really say ensembl learning. For example the first reference EPIP that they cite after their statement, does not use Random forest clustering, but another type of ensembl classifiers.
Response: [222-223] Corrected this statement to align with the definition of ensemble clustering (combine clusters) and ensemble learners (combine models).
Minor:
-Page 3. This sentence: "These include three-dimensional chromosome structure as measured with Hi-C [4], chromatin occu- pancy probed by ATAC-Seq [2] or ChIP-Seq [3], .." is misleading. Obviously ATAC-seq and ChIP-seq do no not measure the 3d-chromatin structure. If anything the 3d-chromatin structure may be inferred from those assays using additional information, but it seems far fetched in the way currently written.
Response: [120-123] Our original wording was unclear, now reworded to avoid the implication that ATAC-seq and ChIP-seq can be used to infer 3D structure.
I would like to point the authors to the zeroSum approach, which is a nice example of intra-modality correction for measurements on different platforms (Molecular signatures that can be transferred across different omics platforms, https://www.ncbi.nlm.nih.gov/pubmed/28881975).
Response: [Table 3] Reviewed zeroSum method and added it to the list, fixed a typo in our original table
Table 2: The caption is too small to be read without zooming in the pdf.
Response: [Table 1-3] Recoded small tables to be more human-readable.
Table 4/5: It would be good to include the citation to the methods in the table. For example the column "Chromatin occupancy and histone marks" could be called: "Epigenetic peak data" to shorten the text.
Response: [Table 4-5] Added citation to methods, renamed columns to optimise space.
In this context I would like to point out that sometimes a method may also include peaks from DNA methylation analysis, which is not entirely clear from the table as there is another column for DNA methylation. But I assume that the column DNA methylation refers to the fact that a method is able to handle the beta values explicitely?
Response: [Table 4-5] Clarified DNA methylation context in table legends
Following the above changes we have also simplified wordings in the conclusion section.

We hope that you find the changes satisfactory and we look forward to hearing from you soon.
With regards
Sonika Tyagi and Tyrone Chen

Close
