# Supplementary material for: Integrative computational epigenomics to build data-driven gene regulation hypotheses
Source: Gigascience. 2020 Jun 16;9(6):giaa064. doi: 10.1093/gigascience/giaa064 (PMC7297091; doi:10.1093/gigascience/giaa064)
Supplement: giaa064_Reviewer_1_Report_Original_Submission — Marcel Holger Schulz -- 5/17/2020 Reviewed [file giaa064_reviewer_1_report_original_submission.pdf]

Reviewer Report

Title: Integrative Computational Epigenomics to build Data-driven Gene Regulation Hypotheses

Version: Original Submission **Date:** 5/17/2020

Reviewer name: Marcel Holger Schulz

Reviewer Comments to Author:

The authors review modern approaches for the integrative analysis of several datasets. I think that the authors provide a good overview of recent methods and the provided tables with classification of supported data types will be useful.
I have a few comments that the authors should address
Comments:
Fig. 1: I would not call it methylation marks. This may be confused with histone methylation, as these are often referred to as histone marks. Instead I would subtitle Methyl-Seq simply with DNA methylation.
Along those lines, it would be nice if the authors could cite a more recent paper about genome-wide methylation sequencing, not only the classical one from 1992, where the basic method was developed but not applied genome-wide.
Fig. 2: The distinct highlighting of measured regions for FAIRE and DNase-seq compared to ATAC-seq is misleading. In principle all of these assays will capture, nucleosome free regions, promoters and gene body. Of course each of these assays, depending on how the exact experimental setup is, will show enrichment in regions that other assays may miss (see a recent comparison we did for example: Unique and assay specific features of NOMe-, ATAC- and DNase I-seq data, NAR 2019).
I would recommend to refer to it as DNaseI-seq, to make clear which Dnase is being used.
Also why do all three assays cover the complete gene body? In fact there is no reason to expect that these would cover anything except nucleosome-free regions in gene bodies, which would be intragenic enhancers most often in introns of genes. Other than that only at the promoter one normally sees strong signals.
Furthermore, I find the marking for methyl-seq also confusing. What is meant by this? Bisulfite sequencing for example covers the whole genome, but only Cytosine methylation is captured. In their current picture this is not clear.
Fig. 3: it would be nice to indicate in the figure what the difference between inter and intra modality means. It may not be obvious to everyone as these terms are not often used, particular not concering the two different categories of inter-modality. They should also be clearly defined in the text. Why do they say that 4 approaches are common, when 5 are shown in the last row of Figure 3? I don't like the arrangement of these 5 approaches in the figure. It is not clear if these are universal or belong to one of the types. I would include something like: Common approaches and then give them numbers from 1 to 5. Or maybe split them according to the use in restricted categories or generic categories? In the figure caption they mention that the 5 approaches are used for inter modality approaches and only mention linear models for intra-modality. I cannot relate to this, in particular in the context of single cell analysis

all of the 5 approaches have been suggested for intra-modality analysis I believe. I think this distinction should be removed. Also what does the black circle with ruler and pen mean?
-When the authors talk about Ensembl clustering (page 4 top, right column), I think they should really say ensembl learning. For example the first reference EPIP that they cite after their statement, does not use Random forest clustering, but another type of ensembl classifiers.
Minor:
-Page 3. This sentence: "These include three-dimensional chromosome structure as measured with Hi-C [4], chromatin occu- pancy probed by ATAC-Seq [2] or ChIP-Seq [3], .." is misleading. Obviously ATAC-seq and ChIP-seq do no not measure the 3d-chromatin structure. If anything the 3d-chromatin structure may be inferred from those assays using additional information, but it seems far fetched in the way currently written.
-I would like to point the authors to the zeroSum approach, which is a nice example of intra-modality correction for measurements on different platforms (Molecular signatures that can be transferred across different omics platforms, https://www.ncbi.nlm.nih.gov/pubmed/28881975).
Table 2: The caption is too small to be read without zooming in the pdf.
Table 4/5: It would be good to include the citation to the methods in the table. For example the column "Chromatin occupancy and histone marks" could be called: "Epigenetic peak data" to shorten the text. In this context I would like to point out that sometimes a method may also include peaks from DNA methylation analysis, which is not entirely clear from the table as there is another column for DNA methylation. But I assume that the column DNA methylation refers to the fact that a method is able to handle the beta values explicitely?
Marcel Schulz

Methods

Are the methods appropriate to the aims of the study, are they well described, and are necessary controls included? Choose an item.

Conclusions

Are the conclusions adequately supported by the data shown? Choose an item.

Reporting Standards

Does the manuscript adhere to the journal’s guidelines on minimum standards of reporting? Choose an item.

Choose an item.

Statistics

Are you able to assess all statistics in the manuscript, including the appropriateness of statistical tests used? Choose an item.

Quality of Written English

Please indicate the quality of language in the manuscript: Choose an item.

Declaration of Competing Interests

Please complete a declaration of competing interests, considering the following questions:

- Have you in the past five years received reimbursements, fees, funding, or salary from an organisation that may in any way gain or lose financially from the publication of this manuscript, either now or in the future?
- Do you hold any stocks or shares in an organisation that may in any way gain or lose financially from the publication of this manuscript, either now or in the future?
- Do you hold or are you currently applying for any patents relating to the content of the manuscript?
- Have you received reimbursements, fees, funding, or salary from an organization that holds or has applied for patents relating to the content of the manuscript?
- Do you have any other financial competing interests?
- Do you have any non-financial competing interests in relation to this paper?

If you can answer no to all of the above, write 'I declare that I have no competing interests' below. If your reply is yes to any, please give details below.

I declare that I have no competing interests

I agree to the open peer review policy of the journal. I understand that my name will be included on my report to the authors and, if the manuscript is accepted for publication, my named report including any attachments I upload will be posted on the website along with the authors' responses. I agree for my report to be made available under an Open Access Creative Commons CC-BY license (http://creativecommons.org/licenses/by/4.0/). I understand that any comments which I do not wish to be included in my named report can be included as confidential comments to the editors, which will not be published.

Choose an item.

To further support our reviewers, we have joined with Publons, where you can gain additional credit to further highlight your hard work (see: https://publons.com/journal/530/gigascience). On publication of this paper, your review will be automatically added to Publons, you can then choose whether or not to claim your Publons credit. I understand this statement.

Yes Choose an item.
